# Synthesis and Antifungal Evaluation Against *Candida* spp. of 5-Arylfuran-2-Carboxamide Derivatives

**DOI:** 10.3390/microorganisms13081835

**Published:** 2025-08-06

**Authors:** Salvatore Mirabile, Giovanna Ginestra, Rosamaria Pennisi, Davide Barreca, Giuseppina Mandalari, Rosaria Gitto

**Affiliations:** Department of Chemical, Biological, Pharmaceutical and Environmental Sciences, University of Messina, Viale F. Stagno D’Alcontres 31, 98166 Messina, Italy; giovanna.ginestra@unime.it (G.G.); rosamaria.pennisi@unime.it (R.P.); davide.barreca@unime.it (D.B.); rosaria.gitto@unime.it (R.G.)

**Keywords:** 5-arylfuran-2-carboxamide derivatives, synthesis, structure–activity relationship (SAR), antifungal activity, cytotoxicity

## Abstract

Candidiasis arises from the proliferation of *Candida* species in the human body, especially in individuals with compromised immune systems. Efficient therapeutic management of candidiasis is often hampered by the limited availability of potent antifungal drugs and the emergence of drug-resistant strains. We have previously identified the *N*-[(4-sulfamoylphenyl)methyl][1,1′-biphenyl]-4-carboxamide to have fungistatic and fungicidal properties, likely due to the hydrophobic biphenyl–chemical features affecting the structural organization of *Candida* spp. cell membrane. Here, we designed and synthesized a novel series of twelve 5-arylfuran-2-carboxamide derivatives bearing a new hydrophobic tail as bioisosteric replacement of the diphenyl fragment. Its antifungal effectiveness against *C. albicans*, *C. glabrata*, and *C. parapsilosis*, including ATCC and clinically isolated strains, was assessed for all compounds. The most active compound was *N*-benzyl-5-(3,4-dichlorophenyl)furan-2-carboxamide (**6**), with fungistatic and fungicidal effects against *C. glabrata* and *C. parapsilosis* strains (MIC = 0.062–0.125 and 0.125–0.250 mg/mL, respectively). No synergistic effects were observed when combined with fluconazole. Interestingly, fluorescent microscopy analysis after staining with SYTO 9 and propidium iodide revealed that compound **6** affected the cell membrane integrity in *C. albicans* strain 16. Finally, carboxamide **6** exhibited a dose-dependent cytotoxicity on erythrocytes, based on assessing the LDH release.

## 1. Introduction

In recent decades, candidiasis has come to represent the most prevalent fungal disease affecting humans; it is one of the major causes of morbidity and/or mortality for the global population [1]. Candidiasis can be caused by various *Candida* species, including resistant strains, which are clinical isolates. The fungal infection includes mucosal/superficial disease as well as systemic invasive pathological events [2]. Moreover, candidiasis is a particularly prevalent emerging risk factor among hospitalized patients [3,4]. Specifically, *C. albicans* is considered responsible for mucosal infections, leading to invasive candidiasis in immunocompromised patients [5]. *Candida parapsilosis* represents a cause of sepsis and infections [6], whereas *Candida glabrata* is relevant in nosocomial infections related to biofilm production and its resistance to antifungal agents [7,8]. The fungal cell wall represents the ideal target for antifungal drug discovery due to its role in cell survival, proliferation, and immune evasion [9]. Currently, three main chemotypes of anti-*Candida* agents are available for clinical applications: azoles [10], polyenes [11], and echinocandins [12,13]; they exert antifungal effects interfering with the plasma membrane or cell wall structural organization via the inhibition of lanosterol-14α-demethylase or β-1,3-d-glucan synthase and by binding with ergosterol [14,15]. Due to the limited antifungal therapeutics available and significant drug resistance phenomena, different efforts are being carried out to identify novel chemotypes to fight various *Candida* spp., including strains isolated in care units [16].

In this context, we sought to explore novel chemical entities based on previously reported hit molecules, which have been serendipitously identified among a class of arylsulfonamides synthesized in our laboratory [17]. Among this class of compounds, several derivatives proved to exert fungistatic and fungicidal effects [17]. For one of the active compounds, i.e., *N*-[(4-sulfamoylphenyl)methyl][1,1′-biphenyl]-4-carboxamide (**I**), we can hypothesize that its antifungal effect might be related to the diphenyl moiety as the crucial lipophilic tail is able to induce the alteration of the lipid components and/or the structural organization of *Candida* spp. cell membrane.

**Figure 1:**
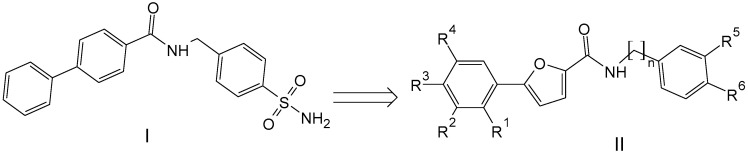
Chemical structures of previously reported *N*-[(4-sulfamoylphenyl)methyl][1,1′-biphenyl]-4-carboxamide (**I**) and designed 5-arylfuran-2-carboxamide derivatives (**II**).

In the present study, we described the synthesis and biological evaluation of a small series of 5-arylfuran-2-carboxamide-based compounds (**II**), which were inspired by prototype molecule **I** (Figure 1). Specifically, the new chemotypes were mainly designed via the structural modification of prototype **I** through the replacement of the “diphenyl fragment” with a chloro-substituted-5-arylfuranic hydrophobic tail; further modification was focused on the secondary aryl moiety bearing distinct polar/nonpolar functional groups. Moreover, additional changes were introduced on the amide linker by removing the methylene bridge or introducing a two-carbon tether as the linking moiety.

The antifungal effectiveness of the new chemotypes was assessed against *C. albicans*, *C. glabrata*, and *C. parapsilosis*, exploring their ability to affect membrane integrity.

## 2. Materials and Methods

### 2.1. Chemistry

Chemicals and solvents were purchased from “Merck Sigma Aldrich” (Milano, Italy) and “TermoFisher Scientific-Alfa Aesar” (Segrate, Italy). Thin-layer chromatography (TLC) was performed using pre-coated silica gel plates (glass sheets) with fluorescent indicator F254 (Merck, 60, F254). Nuclear magnetic resonance (NMR) spectra (^1^H e ^13^C NMR) were recorded on a Varian Gemini 500 (Palo Alto, CA, USA) in CDCl_3_ or DMSO-*d*_6_. NMR chemical shifts (*δ*) were reported in parts per million (ppm) and coupling constants (*J*) in Hertz (Hz). Melting points were recorded on Buchi B-545 (BUCHI Labortechnik AG, Flawil, Svitzerland) and are uncorrected. The purity of compounds was observed to exceed ≥95% in the elemental analyses (C, H, N) recorded with a “Carlo Erba 1106 Analyzer” (Milano, Italy) instrument, so that the found values were within ±0.4% of the calculated values.

#### General Procedure to Synthesize the 5-Arylfuran-2-Carboxamide Derivatives **3**–**14**

The coupling reagent HBTU (1 mmol) was added to a solution of suitable 5-arylfurancarboxylic acid derivative (1 mmol) in *N*,*N*-dimethylformamide (DMF, 4 mL) and the reaction mixture was stirred at room temperature for 1 h. Subsequently, the suitable amine derivative (1 mmol) and DIPEA (1 mmol) were added and the resulting mixture was initially refluxed for 5 h at 100 °C and then maintained at room temperature for 15 h. The progress of the reaction was monitored via TLC with a mixture of hexane and ethyl acetate as the eluant (70:30). After reaction completion, the mixture was diluted with water and extracted with ethyl acetate (three times, 30 mL). The organic phases were collected, washed with saturated brine solution, dried over anhydrous sodium sulfate and finally concentrated in vacuo to give the desired compounds **3**–**14**. The title compounds **3**–**14** were crystallized using ethanol.

**5-(3,4-Dichlorophenyl)-*N*-(4-sulfamoylphenyl)furan-2-carboxamide (3)**

The title compound was prepared according to the general procedure using 5-(3,4-dichlorophenyl)-2-furoic acid (257 mg, 1 mmol) and sulfanilamide (172 mg, 1 mmol) as a yellow powder in a 47% yield (193 mg). M.p. 302–303 °C. ^1^H NMR (500 MHz, DMSO-*d*_6_) δ ppm 7.27 (s, 2 H), 7.36 (d, *J* = 4.0 Hz, 1 H), 7.45 (d, *J* = 4.0 Hz, 1 H), 7.76 (d, *J* = 8.8 Hz, 1 H), 7.82 (d, *J* = 8.8 Hz, 2 H), 7.92–7.95 (m, 3 H), 8.29 (d, *J* = 1.5 Hz, 1 H), 10.50 (s, 1 H). ^13^C NMR (126 MHz, DMSO-*d*_6_) δ ppm 110.0 and 118.3 (2 CH furan ring), 120.7, 125.1, 126.6, 127.0, 130.1, 131.6, 131.7, 132.5, 139.5, 141.7, 147.3 and 153.4 (C-furan ring), 156.4 (C=O). Calculated for (C_17_H_12_Cl_2_N_2_O_4_S): C 49.65, H 2.94, N 6.81. Found: C 49.69, H 3.03, N 6.85.

**5-(3,4-Dichlorophenyl)-*N*-(4-sulfamoylphenylmethyl)furan-2-carboxamide (4)**

The title compound was prepared according to the general procedure using 5-(3,4-dichlorophenyl)-2-furoic acid (257 mg, 1 mmol) and 4-aminomethylbenzenesulfonamide (186 mg, 1 mmol) as white powder in 40% yield (170 mg). M.p.288–290 °C. ^1^H NMR (500 MHz, DMSO-*d*_6_) δ ppm 4.54 (d, *J* = 5.9 Hz, 2 H), 7.20 (d, *J* = 4.0 Hz, 1 H), 7.28 (d, *J* = 3.4 Hz, 1 H), 7.31 (s, 2 H), 7.49 (d, *J* = 8.3 Hz, 2 H), 7.74 (d, *J* = 8.3 Hz, 1 H), 7.77 (d, *J* = 8.3 Hz, 2 H), 7.90–7.92 (m, 1 H), 8.24 (d, *J* = 1.9 Hz, 1 H), 9.27 (t, *J* = 6.1 Hz, 1 H). ^13^C NMR (126 MHz, DMSO-*d*_6_) δ ppm 42.1 (CH_2_), 110.0 and 116.5 (CH, furan ring), 124.9, 126.2, 126.3, 128.0, 130.3, 131.2, 131.6, 132.4, 143.2, 143.9, 147.9, 152.5 (C, furan ring), 158.0 (C=O). Calculated for (C_18_H_14_Cl_2_N_2_O_4_S): C 50.83, H 3.32, N 6.59. Found: C 50.87, H 3.30, N 6.64.

**5-(3,4-Dichlorophenyl)-*N*-(4-sulfamoylphenethyl)furan-2-carboxamide (5)**

The title compound was prepared according to the general procedure using 5-(3,4-dichlorophenyl)-2-furoic acid (257 mg, 1 mmol) and 4-(2-aminoethyl)benzenesulfonamide (200 mg, 1 mmol) as white powder in a 66% yield (290 mg). M.p.238–240 °C. ^1^H NMR (500 MHz, DMSO-*d*_6_) δ ppm 2.93 (t, *J* = 7.3 Hz, 2 H), 3.49–3.53 (m, 2 H), 7.14 (d, *J* = 3.4 Hz, 1 H), 7.24 (d, *J* = 3.9 Hz, 1 H), 7.27 (s, 2 H), 7.44 (d, *J* = 8.3 Hz, 2 H), 7.72–7.75 (m, 3 H), 7.87 (dd, *J* = 8.3, 1.9 Hz, 1 H), 8.20 (d, *J* = 2.4 Hz, 1 H), 8.73 (t, *J* = 5.6 Hz, 1 H). ^13^C NMR (126 MHz, DMSO-*d*_6_) δ ppm 35.4 (CH_2_), 40.4 (CH_2_), 110.0 and 116.1 (CH furan ring), 124.8, 126.2, 126.24, 130.4, 131.2, 131.6, 132.4, 142.6, 144.0, 148.2 and 152.2 (C, furan ring), 157.8 (C=O). Calculated for(C_19_H_16_Cl_2_N_2_O_4_S): C 51.95, H 3.67; N 6.38. Found: C 52.04, H 3.70, N 6.42.

***N*-Benzyl-5-(3,4-dichlorophenyl)furan-2-carboxamide (6)**

The title compound was prepared according to the general procedure using 5-(3,4-dichlorophenyl)-2-furoic acid (257 mg, 1 mmol) and benzylamine (107 mg, 1 mmol) as off-white powder in 66% yield (228 mg). M.p.153–155 °C. ^1^H NMR (500 MHz, DMSO-*d*_6_) δ ppm 4.48 (d, *J* = 6.4 Hz, 2 H), 7.19 (d, *J* = 3.4 Hz, 1 H), 7.21–7.34 (m, 6 H), 7.71 (d, *J* = 8.8 Hz, 1 H), 7.90 (d, *J* = 8.8 Hz, 1 H), 8.23 (d, *J* = 1.9 Hz, 1 H), 9.17 (t, *J* = 6.1 Hz, 1 H). ^13^C NMR (126 MHz, DMSO-*d*_6_) δ ppm 42.3 (CH_2_), 110.0 and 116.3 (furan ring), 124.9, 126.3, 127.3, 127.7, 128.8, 130.4, 131.2, 131.6, 132.4, 139.8, 148.1 and 152.4 (C, furan ring), 157.9 (C=O). Calculated for(C_18_H_13_Cl_2_NO_2_): C 62.45, H 3.79; N 4.05. Found: C 62.48, H 3.72, N 4.10.

**5-(3,4-Dichlorophenyl)-*N*-(2-phenylethyl)furan-2-carboxamide** (**7**)

The title compound was prepared according to the general procedure using 5-(3,4-dichlorophenyl)-2-furoic acid (257 mg, 1 mmol) and 2-phenethylamine (121 mg, 1 mmol) as a white powder in a 70% yield (252 mg). M.p.119–121 °C. ^1^H NMR (500 MHz, DMSO-*d*_6_) δ ppm 2.83–2.86 (m, 2 H), 3.44–3.50 (m, 2 H), 7.14 (d, *J* = 3.4 Hz, 1 H), 7.17–7.31 (m, 6 H), 7.71 (d, *J* = 8.2 Hz, 1 H), 7.82–7.91 (m, 1 H), 8.16–8.23 (m, 1 H), 8.71 (t, *J* = 5.6 Hz, 1 H). ^13^C NMR (126 MHz, DMSO-*d*_6_) δ ppm 35.75 (CH_2_), 40.6 (CH_2_) 109.9 and 116.0 (furan ring), 124.8, 126.2, 126.6, 128.8, 129.1, 130.4, 131.2, 131.6, 132.4, 139.7, 148.2, 152.2 (furan ring) 157.8 (C=O). Calculated for(C_19_H_15_Cl_2_NO_2_): C 63.35; H 4.20; N 3.89. Found: 63.41, H 4.16, N 3.93.

***N*-Benzyl-5-(3-chlorophenyl)furan-2-carboxamide (8)**

The title compound was prepared according to the general procedure using 5-(3-chlorophenyl)-2-furoic acid (222 mg, 1 mmol) and benzylamine (107 mg, 1 mmol) as a white powder in a 78% yield (243 mg). M.p.157–158 °C. ^1^H NMR (500 MHz, CDCl_3_) δ ppm 4.68 (d, *J* = 5.9 Hz, 2 H), 6.72 (br s, 1 H), 6.77 (d, *J* = 3.9 Hz, 1 H), 7.24 (d, *J* = 3.9 Hz, 1 H), 7.29 -7.39 (m, 7 H), 7,55 (dt, *J* = 7.6, 1.4 Hz, 1 H), 7.67 (t, *J* = 1.8 Hz, 1 H). ^13^C NMR (126 MHz, CDCl_3_) δ ppm 43.2 (CH_2_), 108.3 and 116.6 (furan ring), 122.5, 124.4, 127.7, 128.0, 128.6, 128.8, 130.1, 131.2, 134.9, 138.0, 147.3, 153.9 (furan ring), 158.1 (C=O). Calculated for(C_18_H_14_ClNO_2_): C 69.35; H 4.53; N 4.49. Found: 69.40, H 4.57, N 4.52.

***N*-Benzyl-5-(2,3-dichlorophenyl)furan-2-carboxamide (9)**

The title compound was prepared according to the general procedure using 5-(2,3-dichlorophenyl)-2-furoic acid (mg 257, 1 mmol) and benzylamine (107 mg, 1 mmol) as a yellow powder in a 40% yield (138 mg). M.p.120–122 °C. ^1^H NMR (500 MHz, CDCl_3_) δ ppm 4.66 (d, *J* = 6.4 Hz, 2 H), 6.68 (br s, 1 H), 7.17 (d, *J* = 3.4 Hz, 1 H), 7.26–7.40 (m, 7 H), 7.47 (dd, *J* = 8.0, 1,6 Hz, 1 H), 7.68 (dd, *J* = 7.8, 1.5 Hz, 1 H). ^13^C NMR (126 MHz, CDCl_3_) δ ppm 43.2 (CH_2_), 113.6 and 116.2 (furan ring), 126.9, 127.3, 127.7, 127.9, 128.8, 130.2, 130.3, 134.6, 137.9, 147.1, 151.1 (furan ring), 158.0 (C=O). Calculated for(C_18_H_13_Cl_2_NO_2_): C 62.45; H 3.79; N 4.05. Found: 62.42, H 3.75, N 4.09.

***N*-Benzyl-5-(2,5-dichlorophenyl)furan-2-carboxamide (10)**

The title compound was prepared according to the general procedure using 5-(2,5-dichlorophenyl)-2-furoic acid (257 mg, 1 mmol) and benzylamine (107 mg, 1 mmol) as a white powder in a 78% yield (270 mg). M.p.140–141 °C. ^1^H NMR (500 MHz, CDCl_3_) δ ppm 4.67 (d, *J* = 5.9 Hz, 2 H), 6.76 (br s, 1 H), 7.20–7.40 (m, 9 H), 7.77 (d, *J* = 2.4 Hz, 1 H). ^13^C NMR (126 MHz, CDCl_3_) δ ppm 43.2 (CH_2_) 113.8 and 116.4 (furan ring), 127.7, 127.9, 128.8, 129.4, 132.1, 133.0. 137.9, 147.2 and 150.3 (furan ring), 158.0 (C=O). Calculated for(C_18_H_13_Cl_2_NO_2_): C 62.45; H 3.79; N 4.05. Found: 62.48, H 3.82, N 4.00.

**5-(3,4-Dichlorophenyl)-*N*-(3-methoxyphenyl)furan-2-carboxamide** (**11**)

The title compound was prepared according to the general procedure using 5-(3,4-dichlorophenyl)-2-furoic acid (257 mg, 1 mmol) and 3-methoxybenzylamine (137 mg, 1 mmol) as a white powder in a 35% yield (132 mg). M.p. 117–118 °C. ^1^H NMR (500 MHz, CDCl_3_) δ ppm 3.81 (s, 3 H), 4.64 (d, *J* = 5.9 Hz, 2 H), 6.66 (br s, 1 H), 6.77 (d, *J* = 3.4 Hz, 1 H), 6.86 (dd, *J* = 8.3, 2.4 Hz, 1 H), 6.92 (m, 1 H), 6.97 (d, *J* = 7.8 Hz, 1 H), 7.23 (d, *J* = 3.4 Hz, 1 H), 7.29 (t, *J* = 7.9 Hz, 1 H), 7.48–7.51 (m, 2 H), 7.76 (d, *J* = 1.9 Hz, 1 H). ^13^C NMR (126 MHz, CDCl_3_) δ ppm 43.2 (CH_2_), 55.3 (CH_3_), 108.7 (CH furan ring), 113.1, 113.6, 116.7 (CH furan ring), 120.2, 123.6, 126.1, 129.4, 129.9, 130.9, 132.5, 133.3, 139.5, 147.5 and 153.0 (furan ring), 157.9 (C=O), 159.9. Calculated for(C_19_H_15_Cl_2_NO_3_): C 60.66; H 4.02; N 3.72. Found: C 60.70, H 4.08, N 3.75.

**5-(3,4-Dichlorophenyl)-*N*-(4-methoxybenzyl)furan-2-carboxamide** (**12**)

The title compound was prepared according to the general procedure using 5-(3,4-dichlorophenyl)-2-furoic acid (257 mg, 1 mmol) and 4-methoxybenzylamine (137 mg, 1 mmol) as a white powder in a 30% yield (113 mg). M.p. 150–152 °C. ^1^H NMR (500 MHz, CDCl_3_) δ ppm 3.81 (s, 3 H), 4.60 (d, *J* = 5.9 Hz, 2 H), 6.62 (br s, 1 H), 6.76 (d, *J* = 3.4 Hz, 1 H), 6.90 (d, *J* = 8.3 Hz, 2 H), 7.22 (d, *J* = 3.7 Hz, 1 H), 7.32 (d, *J* = 8.3 Hz, 2 H), 7.45–7.50 (m, 2 H), 7.75 (d, *J* = 1.9 Hz, 1 H). ^13^C NMR (126 MHz, CDCl_3_) δ ppm 42.7 (CH_2_), 55.3 (CH_3_) 108.6 and 116.6 (furan ring), 114.2, 123.5, 126.0, 129.4, 129.5, 130.9, 132.5, 133.2, 147.6 and 152.9 (furan ring), 157.9 (C=O), 159.2. Calculated for(C_19_H_15_Cl_2_NO_3_): C 60.66, H 4.02; N 3.72. Found: C 60.61, H 3.98, N 3.77.

**5-(3,4-Dichlorophenyl)-*N*-(3-methylbenzyl)furan-2-carboxamide** (**13**)

The title compound was prepared according to the general procedure using 5-(3,4-dichlorophenyl)-2-furoic acid (mg 257, 1 mmol) and 3-methylbenzylamine (121 mg, 1 mmol) as an off-white powder in a 49% yield (180 mg). M.p. 113–115 °C. ^1^H NMR (500 MHz, CDCl_3_) 2.37 (s, 3 H), 4.63 (d, *J* = 5.9 Hz, 2 H), 6.68 (br s, 1 H), 6.77 (d, *J* = 3.9 Hz, 1 H), 7.15 (d, *J* = 7.3 Hz, 1 H), 7.18–7.21 (m, 1 H), 7.24 (d, *J* = 3.4 Hz, 1 H), 7.25–7.28 (m, 2 H), 7.46–7.51 (m, 2 H), 7.76 (d, *J* = 1.9 Hz, 1 H). ^13^C NMR (126 MHz, CDCl_3_) δ ppm 21.4 (CH_3_) 43.2 (CH_2_), 108.6 and 116.6 (furan ring), 123.5, 125.0, 126.1, 128.5, 128.7, 128.8, 129.5, 130.9, 132.5, 133.2, 137.8, 138.6, 147.6 and 152.9 (furan ring), 157.9 (C=O). Calculated for(C_19_H_15_Cl_2_NO_2_): C 63.35, H 4.20; N 3.89. Found: C 63.40, H 4.24, N 3.85.

**5-(3,4-Dichlorophenyl)-*N*-(4-methylbenzyl)furan-2-carboxamide** (**14**)

The title compound was prepared according to the general procedure using 5-(3,4-dichlorophenyl)-2-furoic acid (257 mg, 1 mmol) and 4-methylbenzylamine (121 mg, 1 mmol) as a white powder in a 25% yield (90 mg). M.p. 144–146 °C. ^1^H NMR (500 MHz, CDCl_3_) δ ppm 2.36 (s, 3 H), 4.62 (d, *J* = 5.9 Hz, 2 H), 6.64 (br s, 1 H), 6.75 (d, *J* = 3.4 Hz, 1 H), 7.19 (d, *J* = 7.8 Hz, 2 H), 7.23 (d, *J* = 3.9 Hz, 1 H), 7.27–7.29 (*J* = 7.8 Hz, 2 H), 7.45–7.50 (m, 2 H), 7.75 (d, *J* = 1.9 Hz, 1 H). ^13^C NMR (126 MHz, CDCl_3_) δ ppm 21.1 (CH_3_) 43.0 (CH_2_), 108.6 and 116.6 (furan ring), 123.5, 126.0, 128.0, 129.46, 129.47, 130.9, 132.5, 133.2, 134.9, 137.5, 147.6 and 152.9 (furan ring), 157.9 (C=O). Calculated for(C_19_H_15_Cl_2_NO_2_): C 63.35; H4.20; N 3.89. Found: 63.38, H 4.28, N 3.95.

### 2.2. Lipophilicity and Chemical Stability

The experimental relative lipophilicity was determined using reversed-phase thin-layer chromatography (RPTLC) on 20 × 20 cm plates coated with C18 silica (HPTLC silica gel RP18 F254 from E. Merck, Darmstadt, Germany). Binary eluent was prepared by mixing a 2/1 (*v*/*v*) mixture of acetone and water. The studied compounds were dissolved in methanol in order to obtain the concentration of 2.0 mg/mL. The plates were dried at 105 °C for 1 h before use. From these working solutions, volumes (0.2 μL) were spotted onto the plates. The experiments were repeated 3 times with different dispositions of the compounds on the plate, so that Rf values were expressed as mean values obtained in triplicate. Chromatograms were developed to the distance of 10 cm from the origin in ascending TLC chambers at room temperature. After developing, the spots were visualized by means of a UV lamp at 254 nm. Based on the retardation coefficients (*R_F_*) obtained from the chromatograms, the relative lipophilicity *R_M_* values (Table 1) were calculated from the experimental *R_F_* values according to the formula *R_M_* = log[(1/*R_F_* − 1]. Higher *R_M_* values indicated higher lipophilicity.

The chemical stability of the tested compounds **3**–**6** was examined in acetate buffer (pH = 5.0) and phosphate buffer solution (PBS, pH = 7.4). Stock solutions at a concentration of 20 mM prepared in DMSO were stored and protected from light at room temperature for four days. Afterwards, the stock solutions of compounds **3**–**6** were diluted 1-fold with PBS or acetate buffer in order to obtain working solutions, which were incubated for specified time intervals at 37 °C. Sample aliquots were taken at 0, 60, and 120 mi. Then, we conducted preliminary evaluations of the stability by using a reverse-phase TLC method on pre-coated TLC-plates RP18 modified silica gel 60 F254 (5 cm × 10 cm, E. Merck, Darmstadt-Germany). Linear ascending development was performed in a chromatographic tank previously saturated with acetone/water (2:1 *v*/*v*). The developed plates were air dried and scanned at 254 nm for UV detection through visual inspection.

### 2.3. Microbial Strains and Culture Conditions

The antimicrobial activity was investigated using the following strains: *Candida albicans* ATCC 10231, 3 clinical strains of *C. albicans* (12, 13, 16), *Candida parapsilosis* ATCC 22019, 2 clinical strains of *C. parapsilosis* (30, 34), *Candida glabrata* DSZM 70614, and 2 clinical strains of *C. glabrata* (9, 33). Strains were grown on Sabouraud Dextrose Broth and Agar (SAB) (Oxoid) at 30 °C for 24 h and tests were performed in RPMI 1640 (Sigma, Milano, Italy) [18].

### 2.4. Susceptibility Studies

All strains were grown on SAB at 30 °C overnight. The initial concentration of the suspension used was 5 × 10^6^ CFU/mL, and it was then diluted with RPMI1640 to reach a final concentration of 5 × 10^4^ CFU/mL. All tested compounds **3**–**14** were dissolved in DMSO at a concentration of 10 mg/mL. The minimum inhibitory concentration (MIC) was determined according to the broth microdilution method following the CLSI guidelines [19]. The microplates were incubated for 24 h at 30 °C and the MIC, expressed in mg/mL, was defined as the lowest concentration of the derivative required to completely inhibit the growth of microorganisms. The minimum fungicidal concentration (MFC) was defined as the lowest concentration of the compound that killed 99.9% of the final inocula after 24–48 h of incubation. MFC was determined by transferring an aliquot (20 μL) from the clear sample onto an agar plate, which was incubated at 30 °C for 48 h. All assays were performed in triplicate on three different days. In order to test the efficacy of the combination of compound **6** and the antifungal compound fluconazole (Sigma Aldrich, Italy) against *C. albicans* strain 16 and *C. glabrata* strain 33, the ‘checkerboard’ procedure was performed [20].

### 2.5. Differential Staining via LIVE/DEAD BacLight Kit (Molecular Probes)

To study the mechanism of action of the most active derivative **6**, a fluorescence microscope analysis was carried out using the Live Dead kit, which provides a two-colour fluorescence assay of cell viability, thus allowing us to quantitatively distinguish live and dead cells in minutes, even in a mixed population. A suspension of either *C. albicans* strain 16, *C. albicans* strain 16 after treatment with compound **6**, or *C. albicans* strain 16 after treatment with compound **6** and fluconazole (1 mL) was centrifuged at 9000× *g* for 10 min and the pellet resuspended with saline (0.85% NaCl, 1 mL). Staining solution (3 µL), prepared by combining equal volumes (1.5 µL) SYTO 9 and propidium iodide, was added and the suspension was incubated for 15 min at RT in the dark. The sample was then filtered through a black polycarbonate filter (0.22 µm size). The observation was performed under a Leica DMRE fluorescence microscope with an I3 filter (450–490 nm/515 nm) (marked as 3: FITC), acquiring images of at least 5 fields of view.

### 2.6. Erythrocytes Isolation

According to the Declaration of Helsinki for research protocols approved by the institutional review boards of the National Institutes of Health (NIH), donor volunteers agreed to participate in the study after being informed of all risks, discomforts, and benefits relating to the research. This study was approved by the ethics committee of the University of Messina (Prot. n°71–23 of 5 April 2023). Blood was obtained via venepuncture from healthy male volunteers and collected in heparinized tubes. The plasma was separated to be further used as solvent for real sample experiments and erythrocytes were washed three times with l0 volume of 0.9% and centrifuged at 2500 rpm for 5 min. During the last washing, the packed cells were resuspended in the incubation buffer at pH 7.4 and utilized for subsequent experiments.

### 2.7. Cellular Internalization and Cytotoxic Effects

Erythrocytes were incubated for 24 hr at 37 °C in the absence or in the presence of 1.000, 0.500, 0.250, 0.125, 0.062, 0.031, 0.015 mg/mL final concentrations of the tested compound **6**. At the end of incubation time, cells were centrifuged at 2500 rpm for 5 min. The supernatant was analysed to detect the release of lactate dehydrogenase (LDH), while the pocked cells were resuspended and washed three times in an incubation buffer and finally used for fluorescence and visible microscopy. The supernatant was utilized to check cytotoxicity by measuring LDH release from damaged cells into the culture medium. LDH activity in the medium was determined using a commercially available kit from BioSystems S.A (Barcelona, Spain). The texted compounds, at the concentrations utilized in the experiments, did not interfere with LDH determination.

### 2.8. Statistical Analysis

Data are presented as means ± standard deviation (S.D.). Data were analysed using one-way analysis of variance (ANOVA). The significance of the difference from the respective controls for each experimental test condition was assayed by using Turkey’s for each paired experiment. A *p* < 0.05 was regarded as indicating a significant difference.

## 3. Results and Discussion

### 3.1. Chemistry

**Scheme 1:**
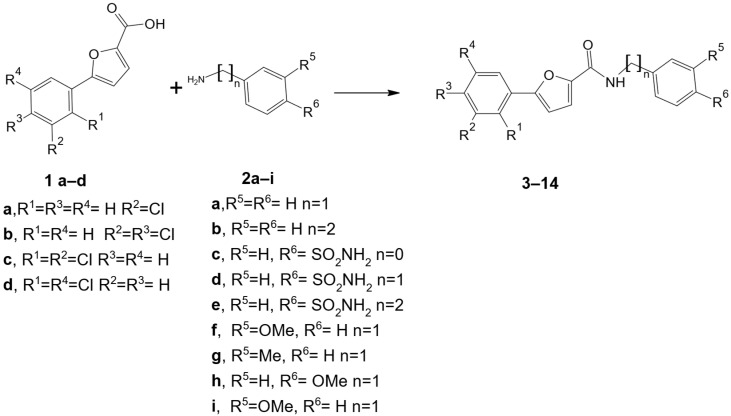
Synthesis of target compounds 5-arylfuran-2-caboxamide derivatives **3**–**14**. Reagents and conditions: HBTU, DIPEA, DMF, 100 °C for 5 h, and then room temperature for 15 h, 25–78%.

As shown in Scheme 1, twelve 5-arylfuran-2-carboxamide derivatives **3**–**14** were prepared via a one-step synthetic route in low to good yields that appeared to mainly be affected by the nature of R^5^/R^6^ substituent. By using commercially available reagents **1a**–**d** and **2a**–**i**, we used feasible routes to obtain this series of compounds for screening purposes and gained information about preliminary structure–activity relationships. First, we retained the sulfonamide moiety and used the 5-arylfuran-2-yl moiety to replace the hydrophobic diphenyl tail of prototype **I** in order to design new amides possessing a furane-ring bearing a phenyl-moiety polysubstituted at the 5-position with chlorine atoms (R^1^, R^2^, R^3^, and R^4^). Second, we introduced hydrophobic substituents (R^5^ and R^6^) in place of the 4′-sulfonamide function to investigate the role of this polar function in generating antifungal effects. Finally, we modified the flexibility of the linking moiety through the selection of distinct amines (**2**) with diverse connecting groups (*n* = 0, 1, and 2).

Starting from 5-aryllfurancarboxylic acid derivatives **1a**–**d**, the coupling reaction with suitable amine derivatives **2a**–**i** produced the target compounds **3**–**14**, for which the pattern of substituents was collected in Table 2. The amidation was performed using hexafluorophosphate benzotriazole tetramethyl uronium (HBTU), commonly used for the activation of free carboxylic acids for peptide synthesis. The reaction was carried out under basic conditions for *N,N*-diisopropylethylamine (DIPEA) in *N*,*N*-dimethylformamide, as previously reported for analog piperazine-based compounds [21]. The structural confirmation and the purity of all the synthesized compounds **3**–**14** were achieved via NMR spectroscopy (^1^H NMR and ^13^C NMR) and elemental analyses, as detailed in Section 2 and reported in the Supplementary Materials.

The predicted drug-like properties of the designed compounds **3**–**14** were evaluated using the SwissADME (https://www.swissadme.ch/ (accessed on 6 March 2024)) web platform (Table 3). Compounds **3**–**14** displayed moderate water solubility according to the ESOL value (Estimated SOLubility); an appropriate lipophilicity (logP < 5) was predicted for the target molecules; as expected, the presence of the sulfonamide moiety improved the values of topological polar surface area (TPSA) in compounds **3**–**5**. All compounds satisfied the filter according to the Lipinski rule, estimated not to provide PAINS alerts and predicted to possess high gastrointestinal (GI) absorbance, as well as comparable bioavailability scores (0.55). Overall, the criteria of the in silico drug-likeness assessment were satisfied.

### 3.2. Antifungal Effects

All the new synthesized 5-arylfuran-2-caboxamide derivatives **3**–**14** were evaluated for their antifungal activity against standard and clinical isolates of *Candida* spp. following the CLSI guidelines (ISBN 1-56238-666-2) [19]. The results are shown in Table 4 and Table 5 and compared with the effects measured for parent compound **I** [17]. All negative controls indicated the absence of inhibition of all the tested strains. A fungistatic activity was recorded for 5-(3,4-dichlorophenyl)-*N*-(4-sulfamoylphenyl)furan-2-carboxamide (**3**) and 5-(3,4-dichlorophenyl)-*N*-(4-sulfamoylphenylmethyl)furan-2-carboxamide (**4**) against *C. parapsilosis* strain 30, whereas 5-(3,4-dichlorophenyl)-*N*-(4-sulfamoylphenylethyl)furan-2-carboxamide (**5**) was active against 50% of the tested strains at concentrations ranging between 0.125 and 1 mg/mL. Interestingly, *N*-benzyl-5-(3,4-dichlorophenyl)furan-2-carboxamide (**6**) was active against all tested strains (MIC values ranging between 0.062 and 1.000 mg/mL), with the exception of *C. glabrata* strain 9. These data indicated that only compound **6** demonstrated similar antifungal effects when compared to compound **I**. A comparative evaluation of the antifungal effects of compounds **4** and **6** revealed that the absence of the sulfonamide moiety significantly enhanced fungistatic activity against all tested strains. As a result, the sulfonamide moiety decorating the four amides **I**, **3**–**5** did not appear to be the crucial chemical feature for active agents. As reported in Table 4, all the other compounds **7**–**14** were not active (MIC values > 1.000 mg/mL), thus implying that the length of amide linker, as well as the chlorine atom positioning, significantly affected the antifungal effects of compounds **7**–**10**. Loss of efficacy was also reported for compounds **11**–**14** containing methyl or methoxy R^5^/R^6^ substituents as electron-donating groups (EDG) on the phenyl ring in place of the sulfonamide as electron withdrawing group (EDW) or hydrogen atom that decorated active compounds **I** and **6**, respectively.

Notably, the fungicidal effect of compound **6** was detected against *C. glabrata* strain 33, *C. albicans* strain 16 and *C. parapsilosis* strain 30 (MFC of 1.000 mg/mL). Furthermore, for compound **6**, a MFC value of 0.500 mg/mL was detected against *C. parapsilosis* strain 34.

The FIC indices calculated for the most effective compound **6** and fluconazole against *C. albicans* strain 16 and *C. glabrata* strain 33 were 1.12 and 2.01, respectively. Since the interpretation of the FIC index depends on which definition is applied, here, we assume the value to be synergistic if the FIC index is ≤0.5, additive or indifferent if it is >0.5 but ≤4, and antagonistic if it is >4 [22]. All the combinations showed an indifferent effect; therefore, no antagonistic or synergistic interaction was observed, as previously found for prototype compound **I** [17].

**Figure 2:**
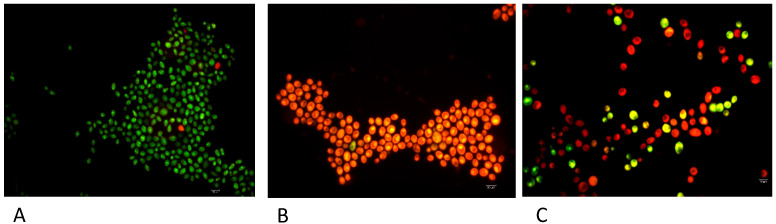
Micrographs showing differential staining of *C. albicans* strain 16 (**A**), *C. albicans* strain 16 after treatment with compound **6** (**B**) and *C. albicans* strain 16 after treatment with compound **6** and fluconazole (**C**). Scale bar = 10 μm.

To acquire information about the mechanism of action of the most active derivative **6**, a fluorescence microscope analysis was carried out using the Live Dead kit, which provides a two-color fluorescence assay of cell viability, thus allowing us to quantitatively distinguish live and dead cells in minutes, even in a mixed population. In more detail, the Live/Dead BacLight Kit uses a mixture of the fluorescent nucleic acid dyes SYTO 9 (green) and propidium iodide (red) [23,24]. These dyes differ in their spectral characteristics and in their ability to penetrate viable microbial cells. Typically, SYTO 9 stains all cells in a population, both those with intact and damaged membranes. On the other hand, propidium iodide only penetrates microbial cells with damaged membranes, resulting in reduced fluorescence of SYTO 9 in the presence of both dyes. Therefore, when using a mixture of SYTO 9 and propidium iodide, fungal cells with intact cell membranes fluoresce green, while cells with damaged membranes fluoresce red. The excitation/emission maxima are 480/500 nm and 490/635 nm for SYTO 9 and propidium iodide, respectively. In agreement with the MIC/MFC values, compound **6** exhibited membrane damage against *C. albicans* strain 16 (Figure 2). As observed by the “checkerboard” procedure, an indifferent effect was recorded with the association of compound **6** with fluconazole against *C. albicans* strain 16.

Considering that the lipophilicity of a bioactive molecule is relevant due to its impact on penetration processes on the cell membrane, we chose to experimentally estimate the relative lipophilicity of synthesized compounds that proved to be anti-candida agents with highly divergent antimicrobial activity. We employed the reverse-phase thin layer chromatography (RP-TLC) method, which has advantages in terms of simple equipment, low mobile phase usage, a high transfer rate, and cheaper cost of analyses. Based on retardation coefficients (*R_F_*) measured from the chromatograms, the *R_M_* values were calculated, obtaining a parameter that displays the relative lipophilicity among the homogenous series of studied compounds **3**–**14**. The Experimental section reports a detailed description of the applied method. The *R_M_* calculation revealed that the studied compounds **3**–**14** possessed *R_M_* values ranging from −0.36 to 0.03, with high values obtained for 3- or 4-methylsubstituted compounds **13**–**14**, whereas the 4-sulfamoyl-substituted compounds **4**–**5** possessed lower lipophilicity with respect to the corresponding unsubstituted compound **6**–**7**; these data were consistent with the predicted values reported in Table 2. However, there was not a clear correlation between the anti-candida effect of compound **6** and its lipophilic properties. Since the tested compounds contained an amide bond, we chose to preliminarily determine the chemical stability of the best active compounds **3**–**6**. Specifically,, we investigated the behavior of compounds **3**–**6** in buffered solutions at acidic and neutral pH. The RP-TLC chromatographic method revealed that the tested compounds possessed good aqueous stability after incubation for various times at 37 °C.

### 3.3. Cytotoxicity Assay

**Figure 3:**
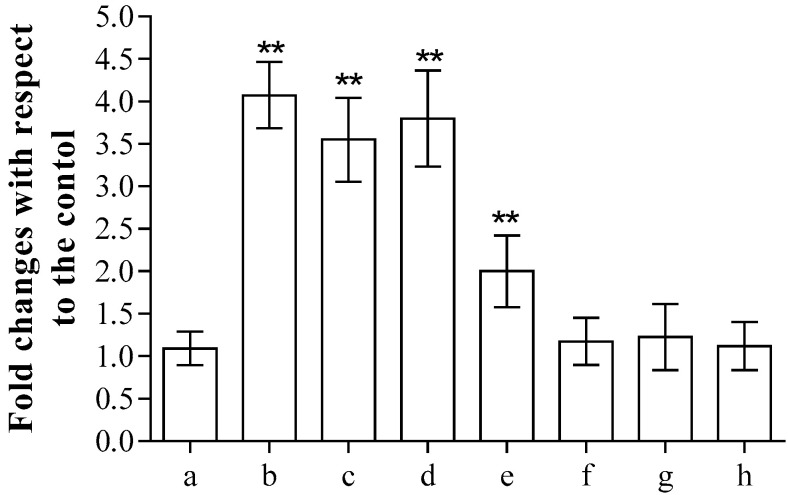
Spectroscopic determination of the release of LDH after incubation in the absence (a) or in the presence of 1.000 (b), 0.500 (c), 0.250 (d), 0.125 (e), 0.062 (f), 0.031 (g) or 0.015 (h) mg/mL of compound **6**. Samples were examined using one-way ANOVA and Turkey’s test. Asterisks (**) are used to indicate a considerable difference from controls (*p* < 0.05).

Finally, as far as antimicrobial potential was concerned, the most promising compound **6** was analysed to test its potential cytotoxicity, as previously reported [17]. After 24 h incubation, the release of lactate dehydrogenase (LDH) from the erythrocytes into the medium, commonly used as a marker of membrane integrity, clearly showed that concentrations above 0.062 mg/mL produced cell damage, while lower concentrations did not induce significant alterations in the cell structure compared to the control (Figure 3). The cytotoxicity of the tested compound is high in the range between 0.250 and 1.000 mg/mL.

## 4. Conclusions

In conclusion, a series of 5-arylfuran-2-carboxamide derivatives was designed to develop antifungal agents that affect the membrane cell integrity through their specific lipophilic structural chemical fragments. Compound **6** exhibited promising antifungal effects against selected *Candida* spp., likely due to the loss of fungal cell membrane integrity. Interestingly, the introduced structural modifications exerted a fine tuning of fungistatic and fungicidal properties for this class of compounds, thus suggesting a specific mode of action in generating membrane damage. Additionally, the sulfonamide substituent was a relevant, though not crucial, chemical feature in the inhibition of *Candida* spp. growth. Overall, compound **6** might represent a novel potential hit molecule for further structural optimization to improve antifungal effects and reduce its cytotoxicity.

## Figures and Tables

**Table 1:** Relative lipophilicity R*_M_* values for compounds **3**–**14**.

Compound	R_M_	Compound	R_M_	Compound	R_M_
**3**	−0.30	**7**	0.01	**11**	−0.07
**4**	−0.35	**8**	−0.17	**12**	−0.09
**5**	−0.36	**9**	−0.07	**13**	0.03
**6**	−0.04	**10**	−0.04	**14**	0.03

**Table 2:**
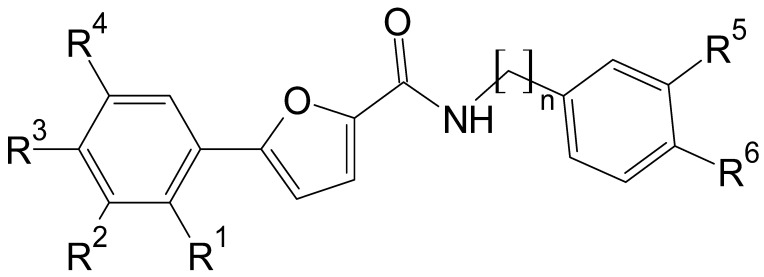
Chemical structures with n, R^1^, R^2^, R^3^, R^4^, R^5^, and R^6^ labels of synthesized compounds **3**–**14**.

Compound	n	R^1^	R^2^	R^3^	R^4^	R^5^	R^6^
**3**	0	H	Cl	Cl	H	H	SO_2_NH_2_
**4**	1	H	Cl	Cl	H	H	SO_2_NH_2_
**5**	2	H	Cl	Cl	H	H	SO_2_NH_2_
**6**	1	H	Cl	Cl	H	H	H
**7**	2	H	Cl	Cl	H	H	H
**8**	1	H	Cl	H	H	H	H
**9**	1	Cl	Cl	H	H	H	H
**10**	1	Cl	H	H	Cl	H	H
**11**	1	H	Cl	Cl	H	OMe	H
**12**	1	H	Cl	Cl	H	H	OMe
**13**	1	H	Cl	Cl	H	Me	H
**14**	1	H	Cl	Cl	H	H	Me

**Table 3:** Predicted drug-like properties of compounds **3**–**14** using SwissADME webtool (https://www.swissadme.ch/ (accessed on 6 March 2024)).

Compound	TPSA ^a^	Consensus Log P	ESOL Class ^b^	GI Absorption	Lipinski Violations	Bioavailability Score	PAINS Alerts
**3**	110.78	3.21	Moderately soluble	High	0	0.55	0
**4**	110.78	3.17	Moderately soluble	High	0	0.55	0
**5**	110.78	3.46	Moderately soluble	High	0	0.55	0
**6**	42.24	4.47	Moderately soluble	High	0	0.55	0
**7**	42.24	4.69	Moderately soluble	High	0	0.55	0
**8**	42.24	3.94	Moderately soluble	High	0	0.55	0
**9**	42.24	4.46	Moderately soluble	High	0	0.55	0
**10**	42.24	4.48	Moderately soluble	High	0	0.55	0
**11**	51.47	4.40	Moderately soluble	High	0	0.55	0
**12**	51.47	4.47	Moderately soluble	High	0	0.55	0
**13**	42.24	4.79	Moderately soluble	High	0	0.55	0
**14**	42.24	4.82	Moderately soluble	High	0	0.55	0

^a^ Topological polar surface area; ^b^ Estimated solubility parameter.

**Table 4:** MICs of compounds **3**–**6**, parent compound **I** and fluconazole (expressed as mg/mL) against *Candida* spp.

STRAIN	Fluconazole	I ^a,b^	3 ^b^	4 ^b^	5 ^b^	6 ^b^
*Candida glabrata* strain *9*	0.031–0.015	>1.000	>1.000	>1.000	>1.000	>1.000
*Candida glabrata* strain *33*	0.500	0.125–0.250	>1.000	>1.000	0.500–1000	0.062–0.125
*Candida glabrata* DSZM 70614	0.062	>1.000	>1.000	>1.000	>1.000	1.000
*Candida parapsilosis* strain *30*	0.031	0.250	0.500–1000	0.500–1000	0.125–0.250	0.125–0.250
*Candida parapsilosis* strain *34*	0.031	0.500	>1.000	>1.000	0.500–1000	0.250
*Candida parapsilosis* ATCC 22019	0.062–0.031	0.125–0.250	>1.000	>1.000	>1.000	0.500–1000
*Candida albicans* strain *12*	0.031–0.015	0.250	>1.000	>1.000	1.000	0.500–1000
*Candida albicans* strain *13*	0.500	0.250	>1.000	>1.000	>1.000	0.500–1000
*Candida albicans* strain *16*	0.500	0.125–0.250	>1.000	>1.000	>1.000	0.500
*Candida albicans ATCC 10231*	0.500	0.250	>1.000	>1.000	1.000	0.500–1000

^a^ Data taken from reference [17]. ^b^ Results represent the means of three independent assays.

**Table 5:** MICs of compounds **7**–**14** and fluconazole against *Candida* spp.

STRAIN	Fluconazole	7 ^b^	8 ^b^	9 ^b^	10 ^b^	11 ^b^	12 ^b^	13 ^b^	14 ^b^
*Candida glabrata* strain *9*	0.031–0.015	>1.000	>1.000	>1.000	>1.000	>1.000	>1.000	>1.000	>1.000
*Candida glabrata* strain *33*	0.500	>1.000	>1.000	>1.000	>1.000	>1.000	>1.000	>1.000	>1.000
*Candida glabrata* DSZM 70614	0.062	>1.000	>1.000	>1.000	>1.000	>1.000	>1.000	>1.000	>1.000
*Candida parapsilosis* strain *30*	0.031	>1.000	>1.000	>1.000	>1.000	>1.000	>1.000	>1.000	>1.000
*Candida parapsilosis* strain *34*	0.031	>1.000	>1.000	>1.000	>1.000	>1.000	>1.000	>1.000	>1.000
*Candida parapsilosis* ATCC 22019	0.062–0.031	>1.000	>1.000	>1.000	>1.000	>1.000	>1.000	>1.000	>1.000
*Candida albicans* strain *12*	0.031–0.015	>1.000	>1.000	>1.000	>1.000	>1.000	>1.000	>1.000	>1.000
*Candida albicans* strain *13*	0.500	>1.000	>1.000	>1.000	>1.000	>1.000	>1.000	>1.000	>1.000
*Candida albicans* strain *16*	0.500	>1.000	>1.000	>1.000	>1.000	>1.000	>1.000	>1.000	>1.000
*Candida albicans ATCC 10231*	0.500	>1.000	>1.000	>1.000	>1.000	>1.000	>1.000	>1.000	>1.000

^b^ Results represent the means of triplicate independent assays.

## Data Availability

The original contributions presented in this study are included in the article/supplementary material. Further inquiries can be directed to the corresponding authors.

## Supplementary Materials

The following supporting information can be downloaded at: https://www.mdpi.com/article/10.3390/microorganisms13081835/s1, Figures S1–S48: ^1^H and ^13^C NMR spectra for synthesized 5-arylfuran-2-carboxamide derivatives **3**–**14**.

## Abbreviations

The following abbreviations are used in this manuscript:

ATCC	American-Type Culture Collection
CDCl_3_	Deuterated chloroform
CLSI	Clinical and Laboratory Standards Institute
ESOL	Estimated solubility
DIPEA	*N*,*N-*Diisopropylethylamine
DMF	*N*,*N-*Dimethylformamide
DMSO-d_6_	Deuterated dimethyl sulfoxide
FIC	Fractional inhibitory concentration
HBTU	Hexafluorophosphate benzotriazole tetramethyl uronium
HPTLC	High-performance thin-layer chromatography
LDH	Lactate dehydrogenase
MFC	Minimum fungicidal concentration
MIC	Minimum inhibitory concentration
NMR	Nuclear magnetic resonance
PBS	Phosphate buffer solution
RP-TLC	Reversed-phase thin-layer chromatography
TPSA	Topological polar surface area
