# Supplementary material for: Synthesis and Antifungal Evaluation Against Candida spp. of 5-Arylfuran-2-Carboxamide Derivatives
Source: Microorganisms. 2025 Aug 6;13(8):1835. doi: 10.3390/microorganisms13081835 (PMC12388132; doi:10.3390/microorganisms13081835)
Supplement: Supplementary file 1 [file microorganisms-13-01835-s001.zip › microorganisms-3787911-supplementary.pdf]

Supplementary data

Synthesis and antifungal evaluation against *Candida spp.* of 5-arylfuran-2-carboxamide derivatives

Salvatore Mirabile [1,*], Giovanna Ginestra [1], Rosamaria Pennisi [1], Davide Barreca [1], Giuseppina Mandalari [1,*] and Rosaria Gitto [1]

[1] *Department of Chemical, Biological, Pharmaceutical, and Environmental Sciences, University of Messina, Viale F. Stagno D'Alcontres 31, 98166 Messina, Italy*

*** Correspondence:** salvatore.mirabile@unime.it; giuseppina.mandalari@unime.it

Contents

Figures S1-48: ^{1}H and ^{13}C NMR spectra for synthesized 5-arylfuran-2-carboxamide derivatives **3-14**

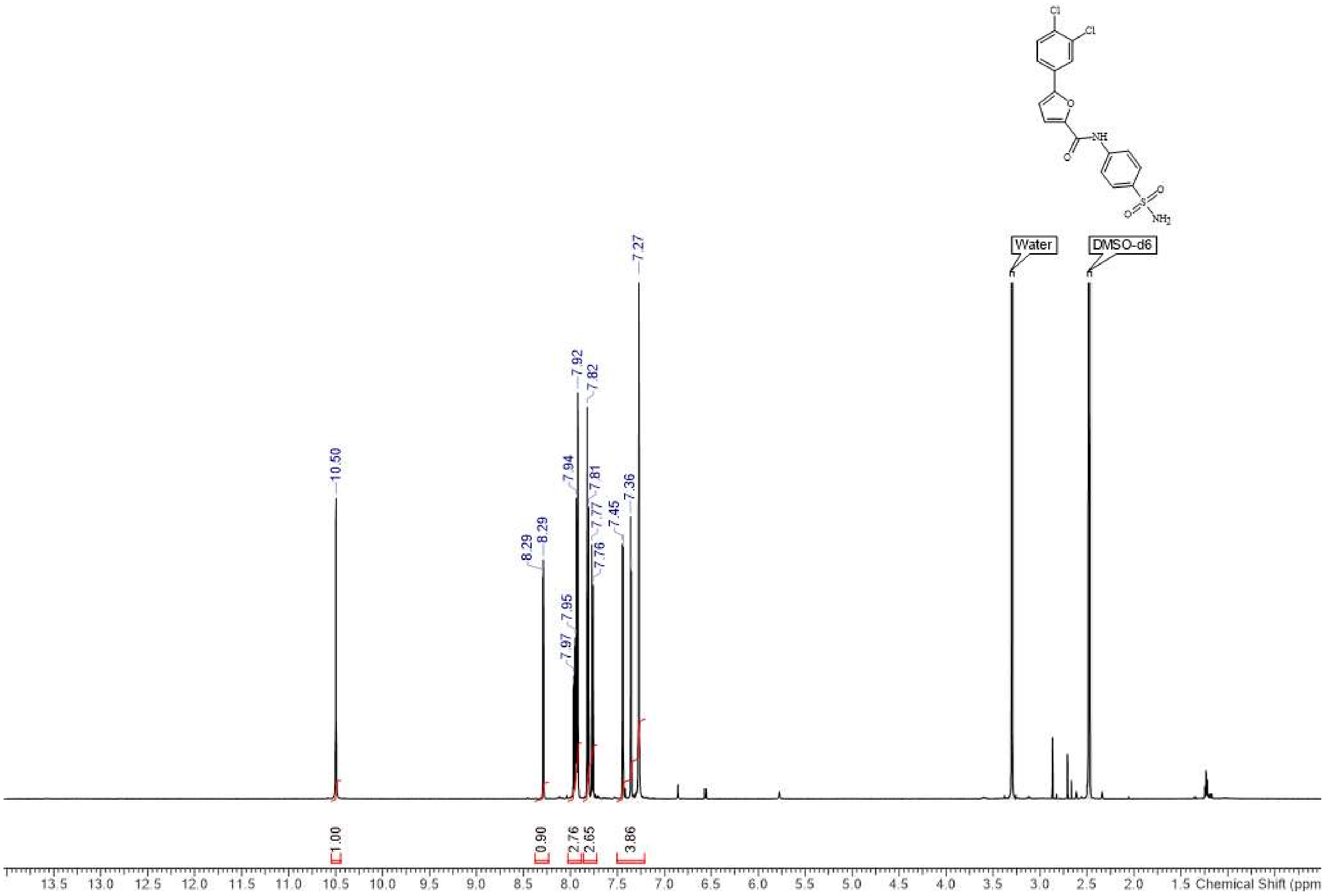

Figure S1: ^{1}H-NMR (500 MHz, DMSO-d_6) spectrum of 5-(3,4-Dichlorophenyl)-N-(4-sulfamoylphenyl)furan-2-carboxamide (3)

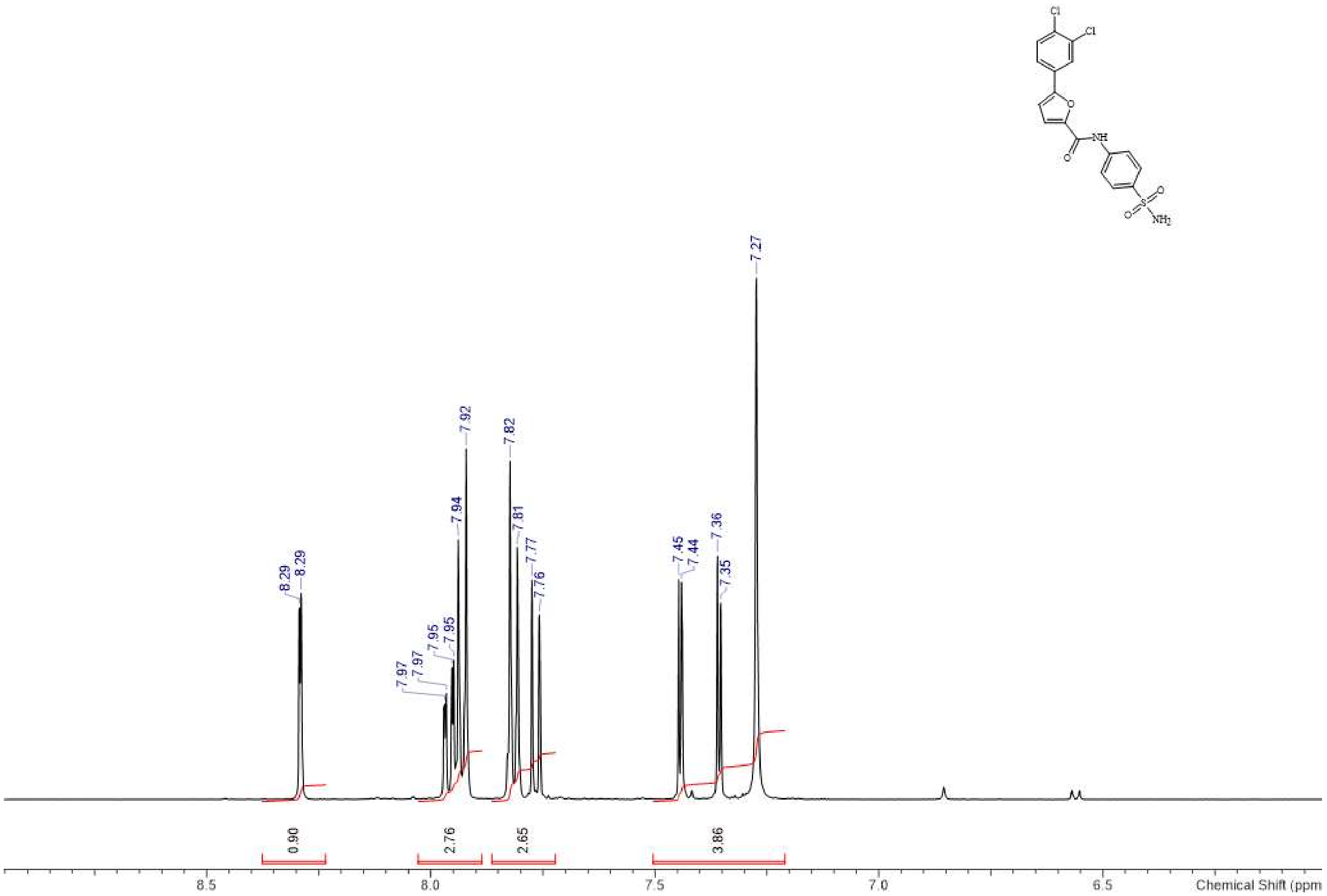

Figure S2: ^{1}H-NMR (500 MHz, DMSO-d_6) zoom of spectrum of 5-(3,4-Dichlorophenyl)-N-(4-sulfamoylphenyl)furan-2-carboxamide (3)

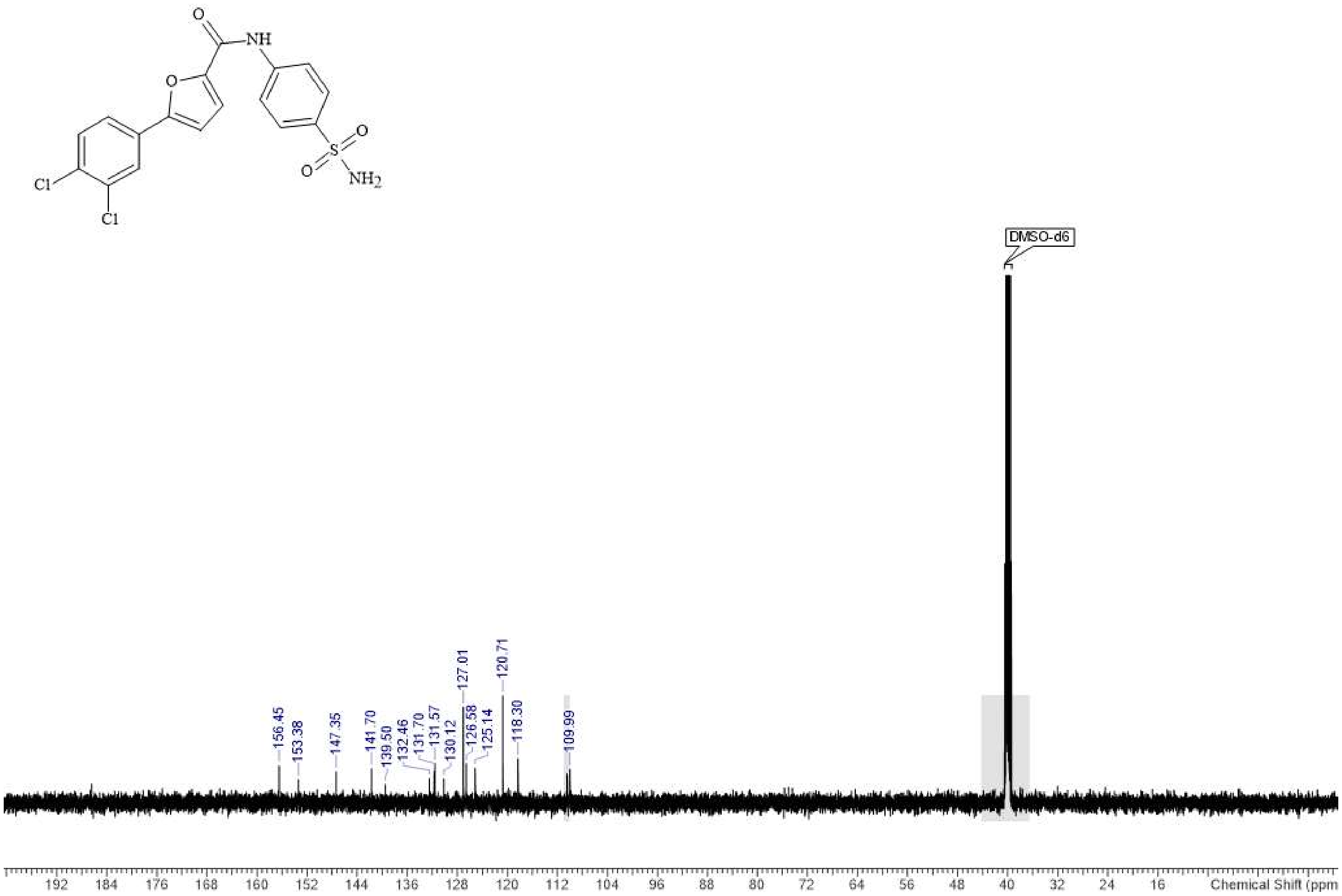

Figure S3: ^{13}C-NMR (126 MHz, DMSO-d_6) spectrum of 5-(3,4-dichlorophenyl)-*N*-(4-sulfamoylphenyl)furan-2-carboxamide (3)

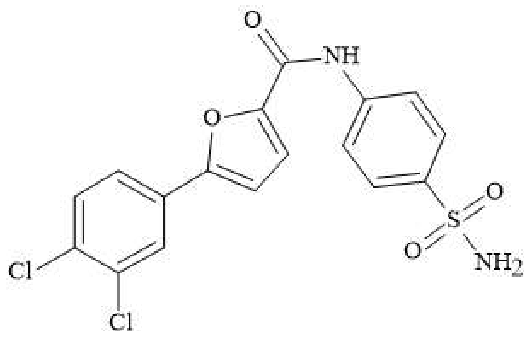

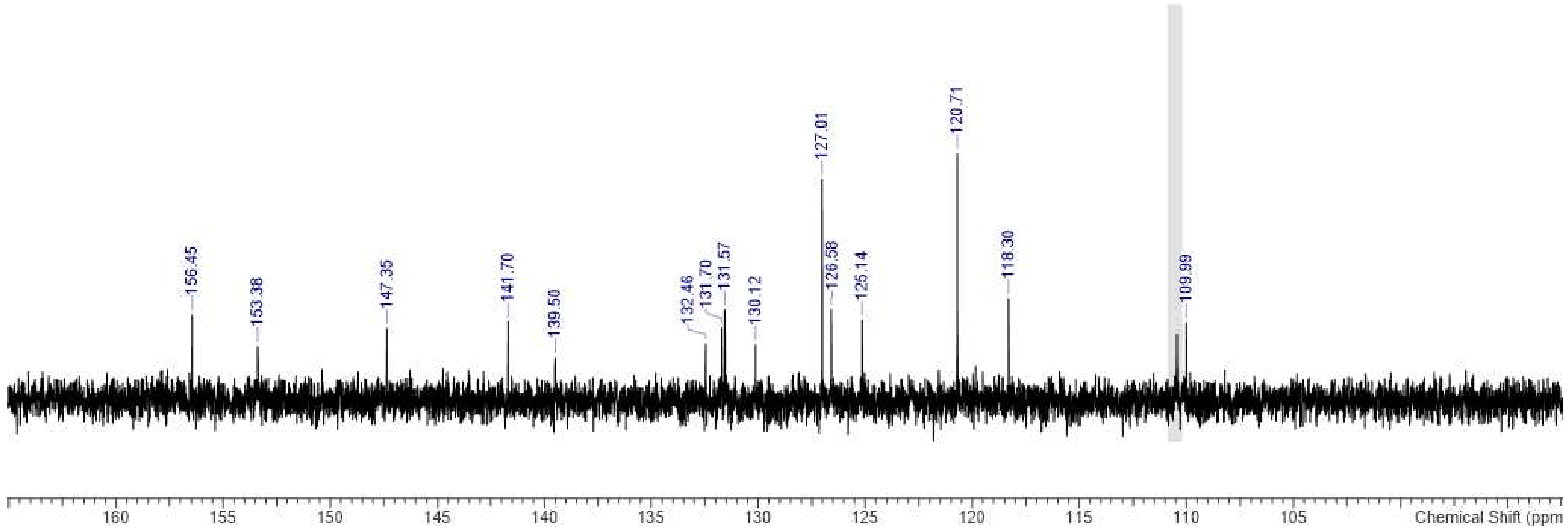

Figure S4: ^{13}C-NMR (126 MHz, DMSO-d_6) zoom of spectrum of 5-(3,4-dichlorophenyl)-*N*-(4-sulfamoylphenyl)furan-2-carboxamide (3)

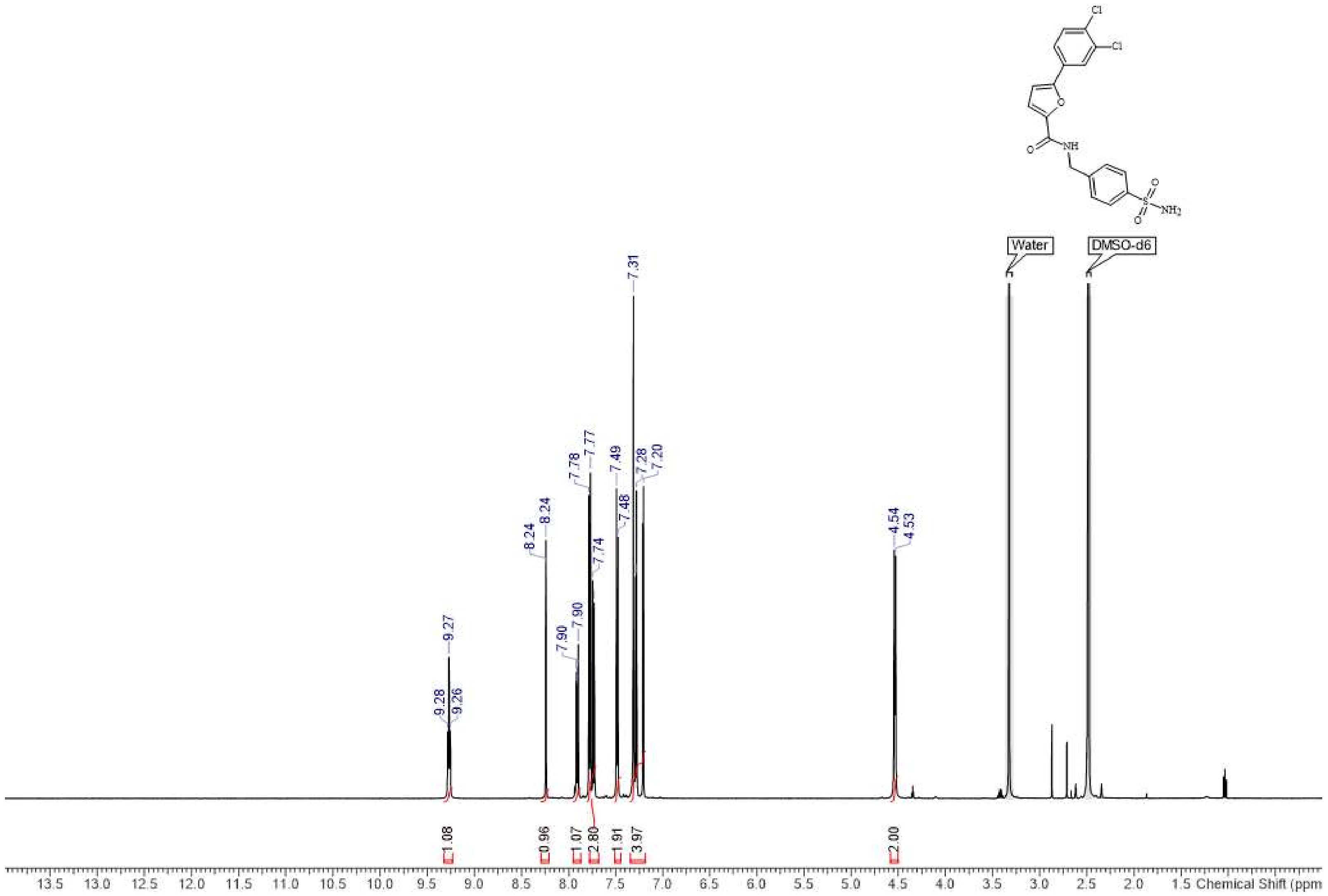

Figure S5: ^{1}H-NMR (500 MHz, DMSO-d_6) spectrum of 5-(3,4-dichlorophenyl)-*N*-(4-sulfamoylphenylmethyl)furan-2-carboxamide (4)

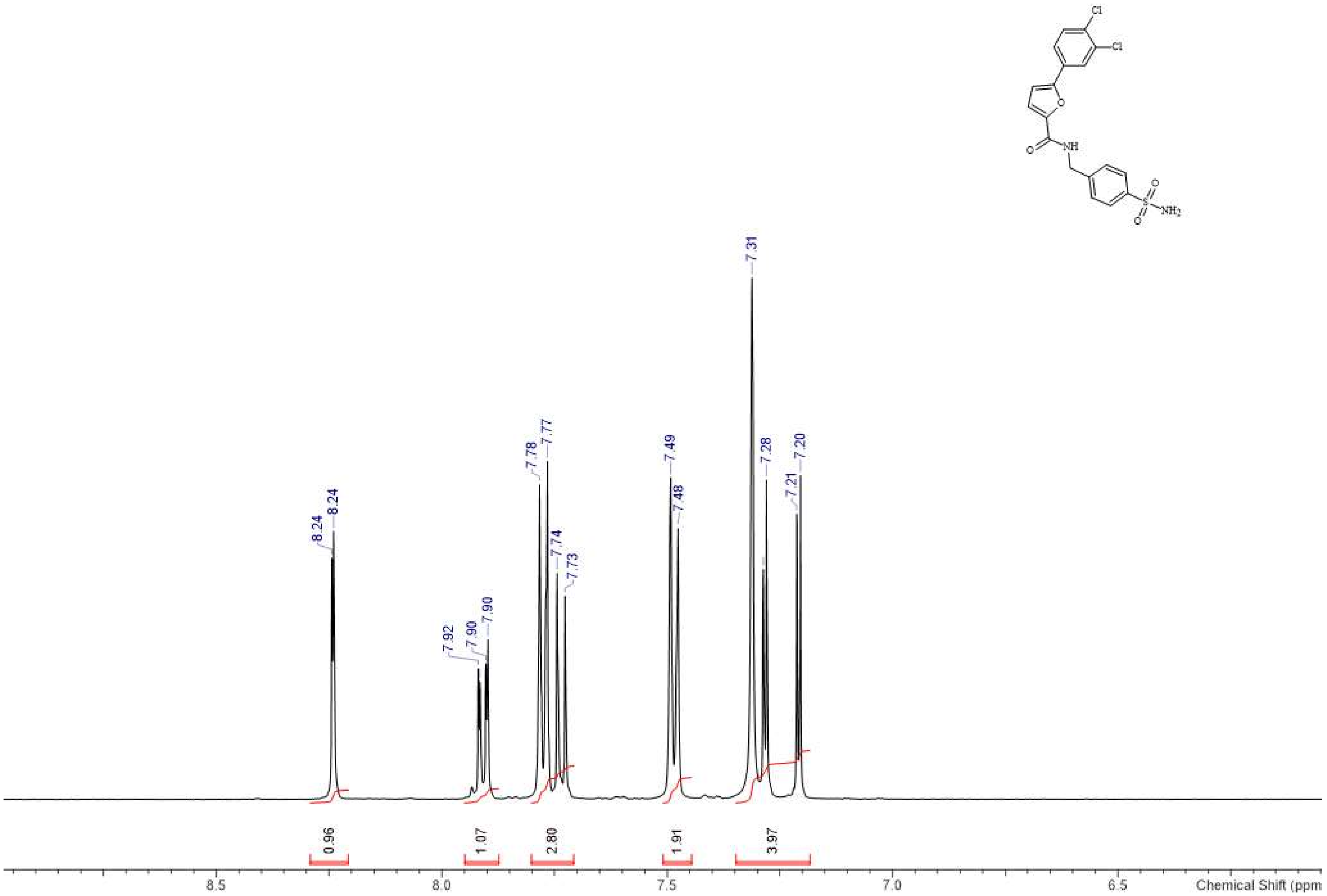

Figure S6: ^{1}H-NMR (500 MHz, DMSO-d_6) zoom of spectrum of 5-(3,4-dichlorophenyl)-*N*-(4-sulfamoylphenylmethyl)furan-2-carboxamide (4)

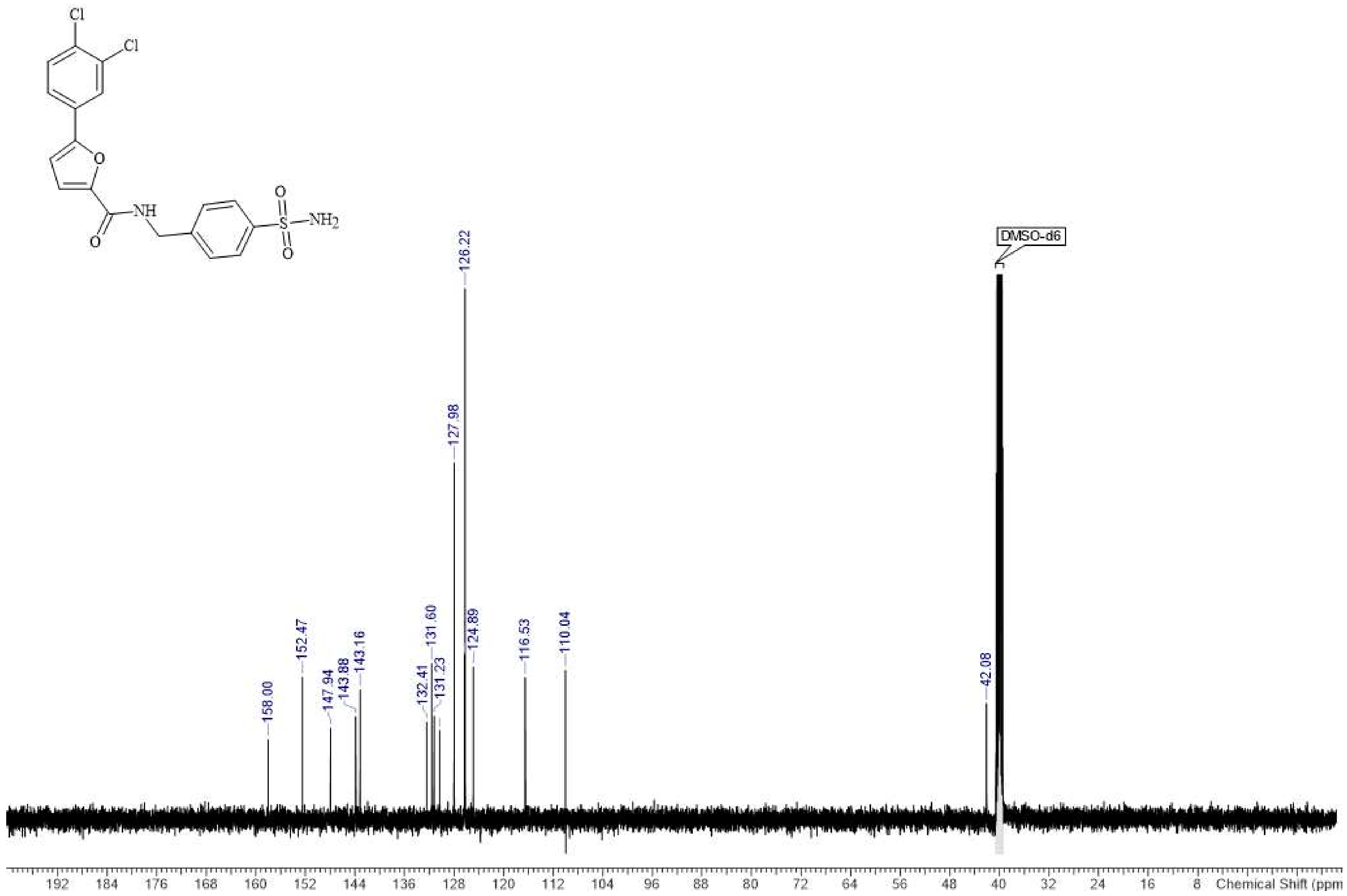

Figure S7: ^{13}C-NMR (126 MHz, DMSO-d_6) spectrum of 5-(3,4-dichlorophenyl)-*N*-(4-sulfamoylphenylmethyl)furan-2-carboxamide (4)

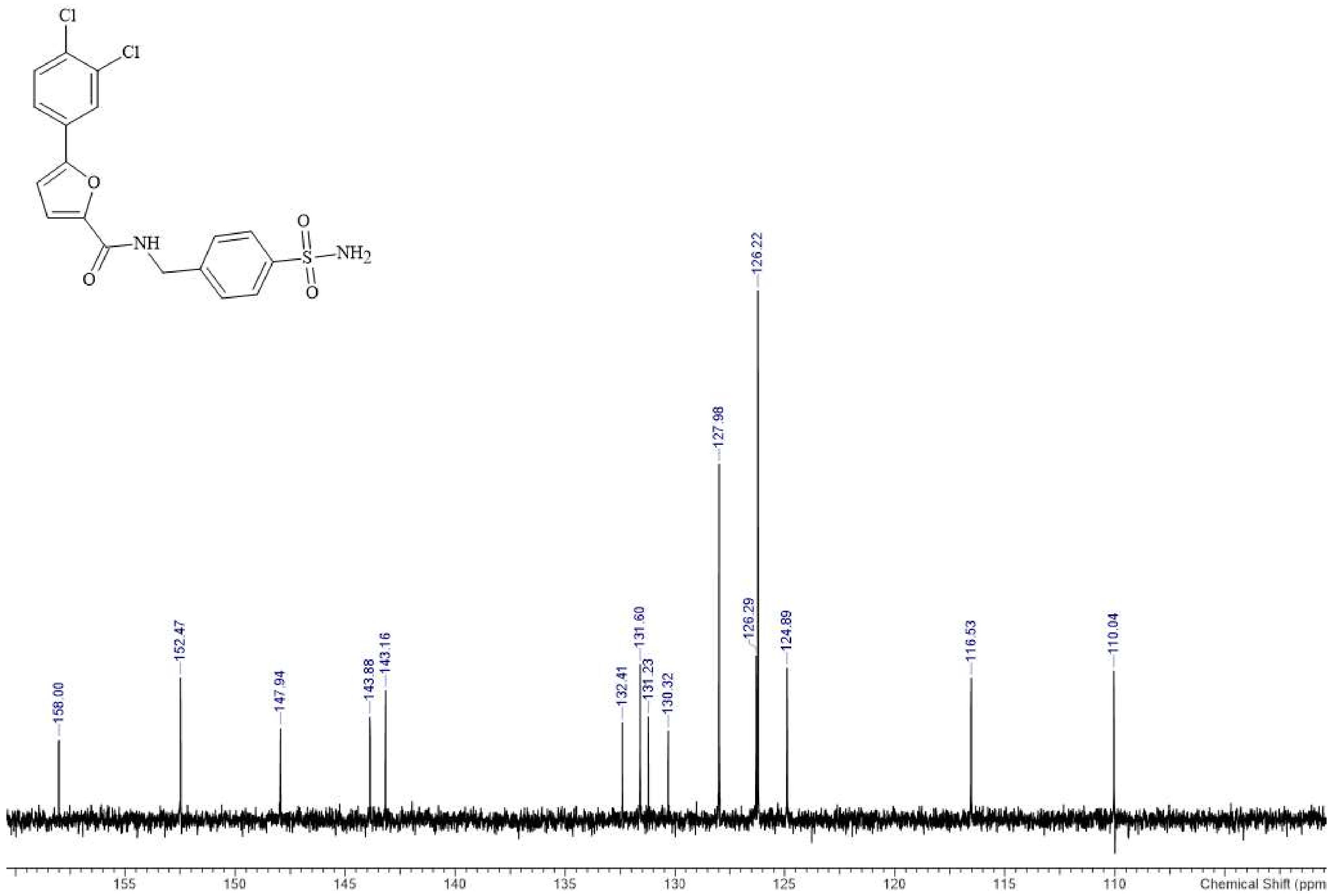

Figure S8: ^{13}C-NMR (126 MHz, DMSO-d_6) zoom of spectrum of 5-(3,4-dichlorophenyl)-*N*-(4-sulfamoylphenylmethyl)furan-2-carboxamide (4)

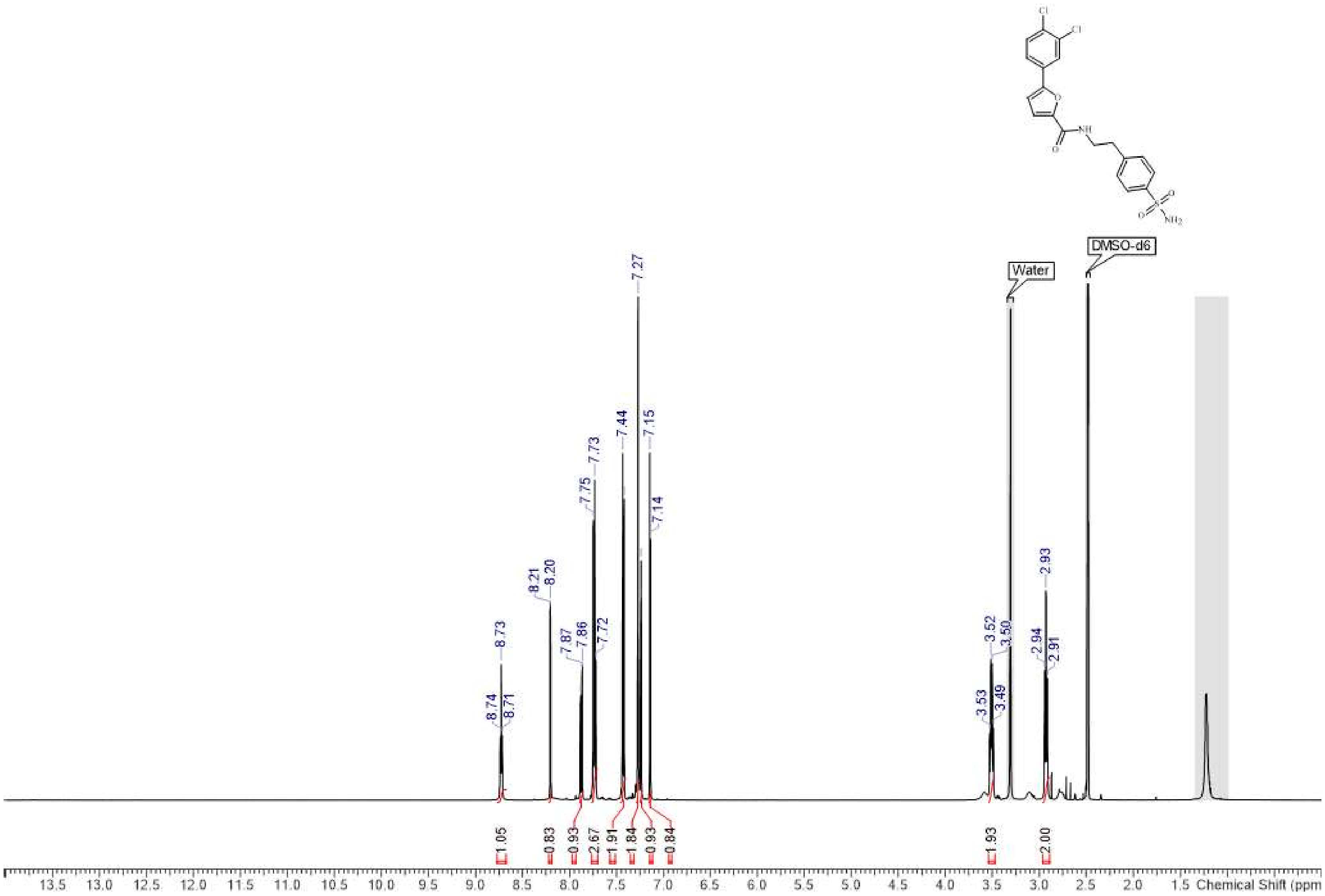

Figure S9: ^{1}H-NMR (500 MHz, DMSO-d_6) spectrum of 5-(3,4-dichlorophenyl)-*N*-(4-sulfamoylphenethyl)furan-2-carboxamide (5)

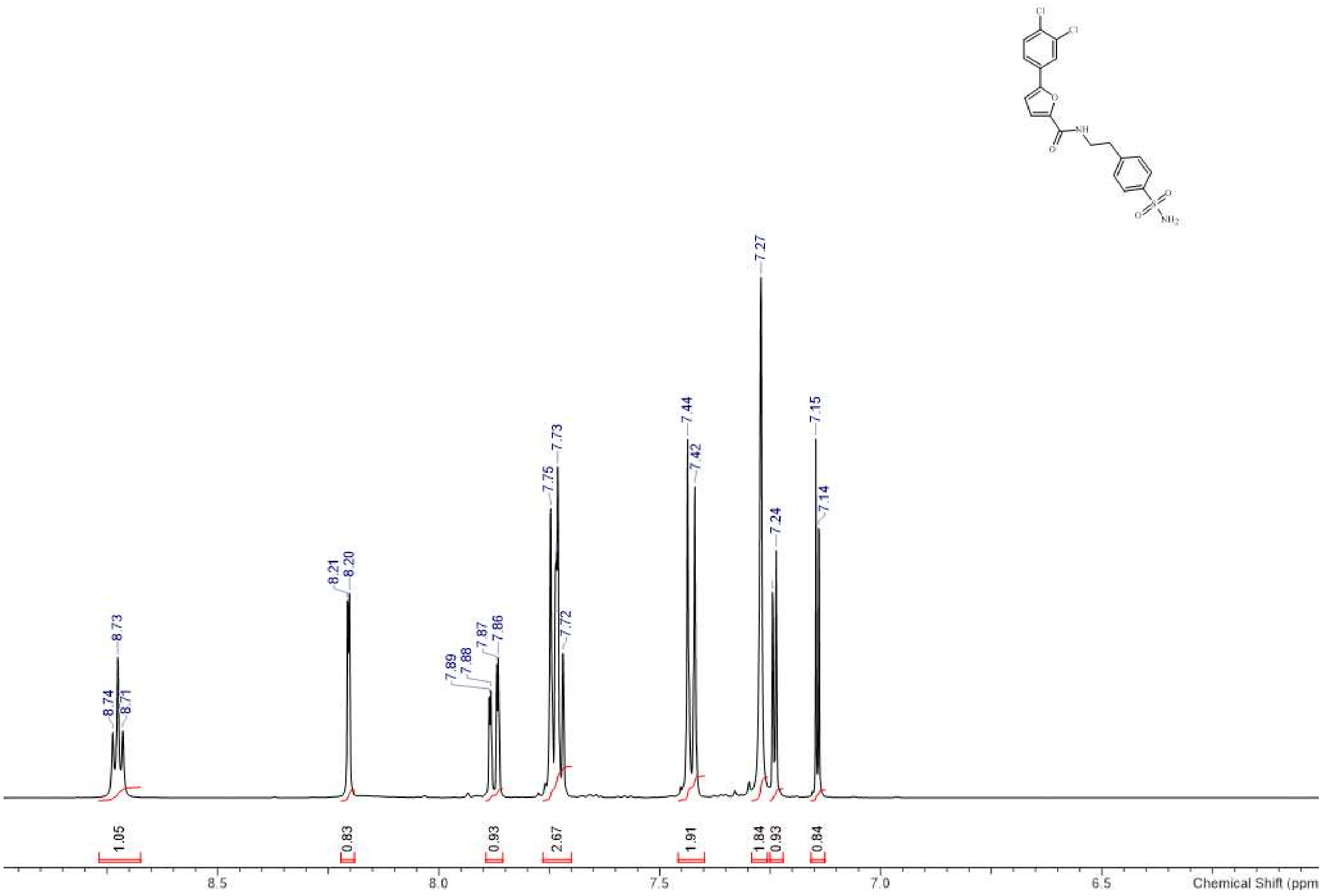

Figure S10: ^{1}H-NMR (500 MHz, DMSO-d_6) zoom of spectrum of 5-(3,4-dichlorophenyl)-*N*-(4-sulfamoylphenethyl)furan-2-carboxamide (5)

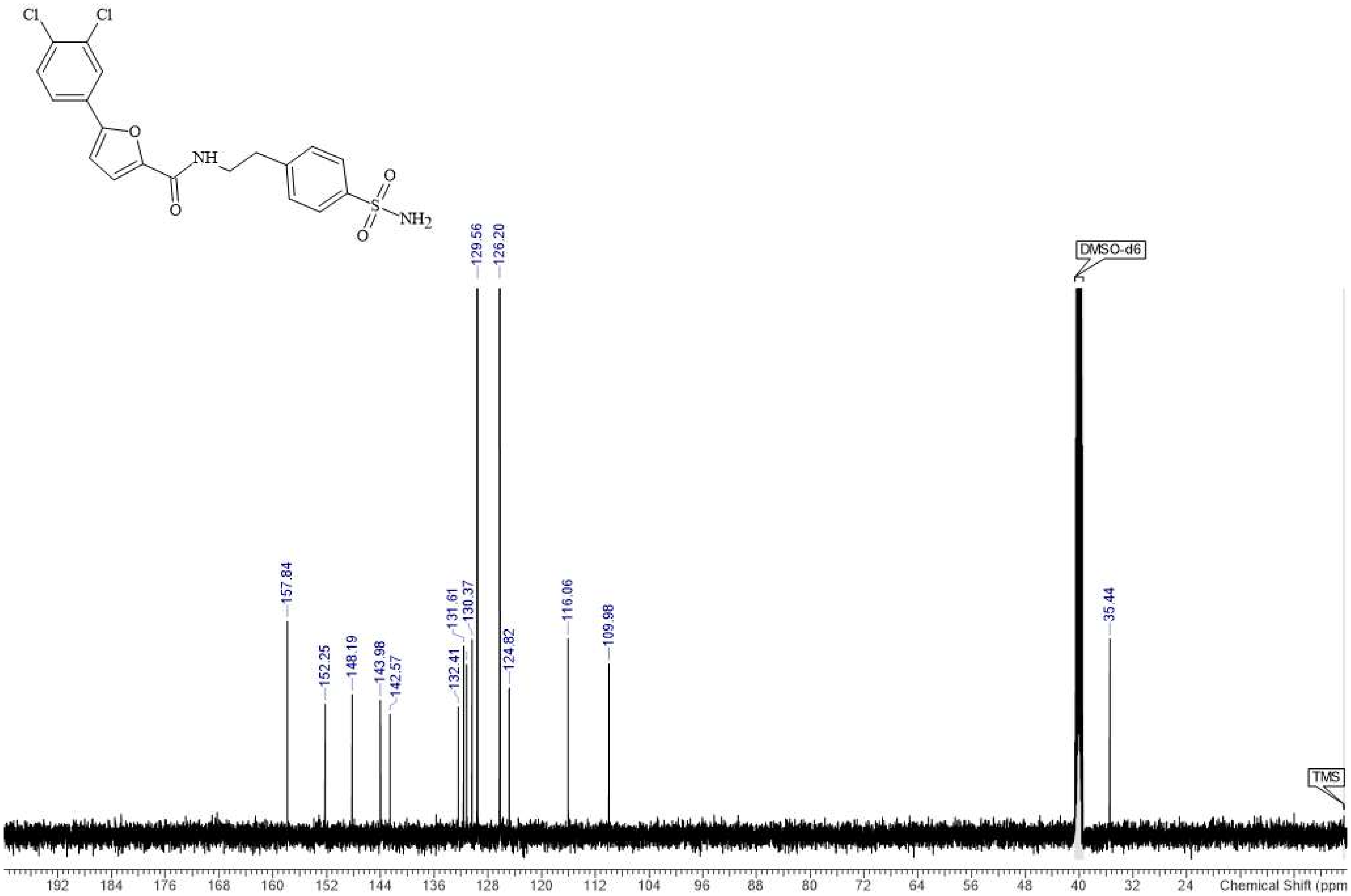

Figure S11: ^{13}C-NMR (126 MHz, DMSO-d_6) spectrum of 5-(3,4-dichlorophenyl)-*N*-(4-sulfamoylphenethyl)furan-2-carboxamide (5)

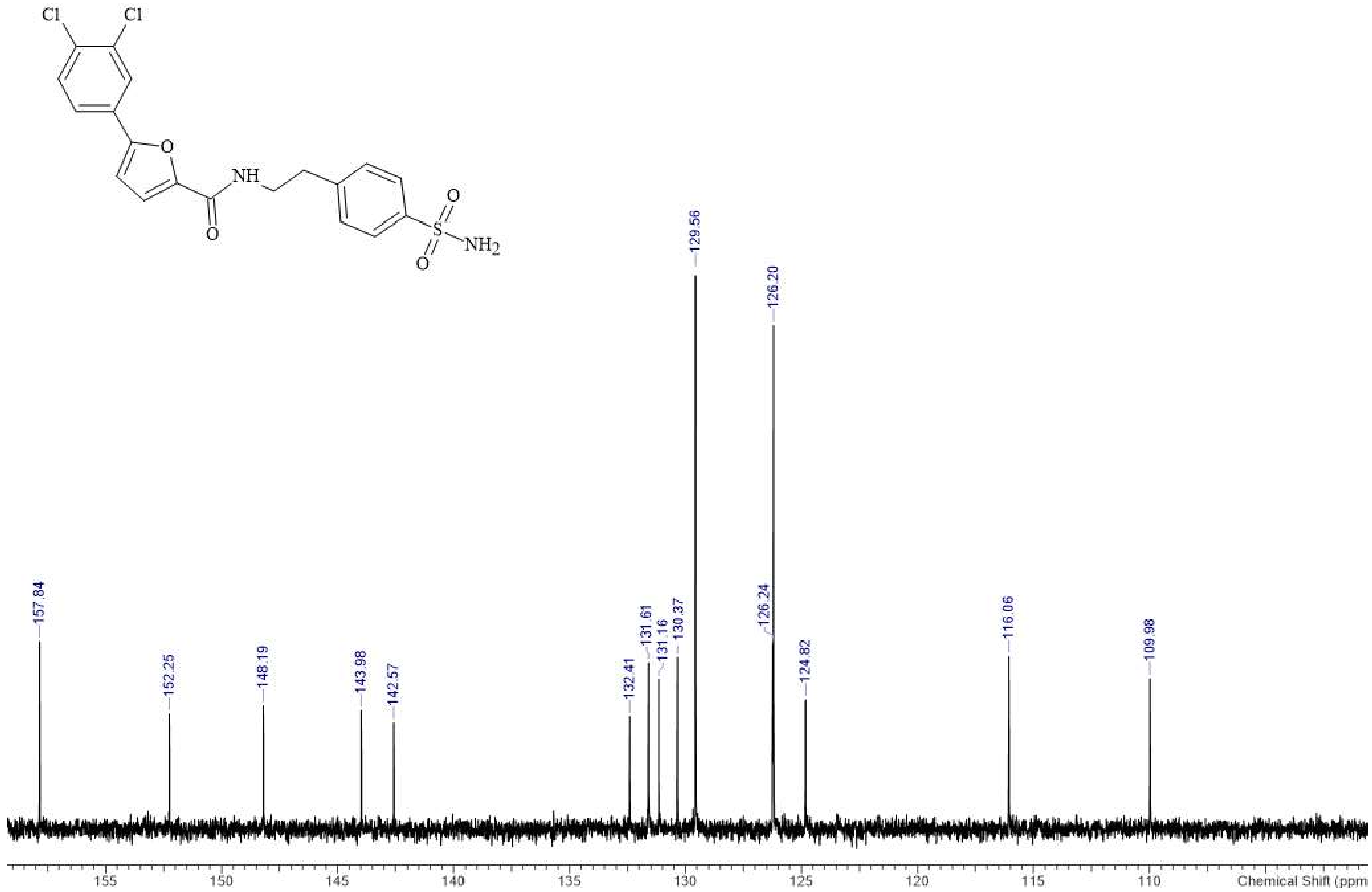

Figure S12: ^{13}C-NMR (126 MHz, DMSO-d6) zoom of spectrum of 5-(3,4-dichlorophenyl)-N-(4-sulfamoylphenethyl)furan-2-carboxamide (5)

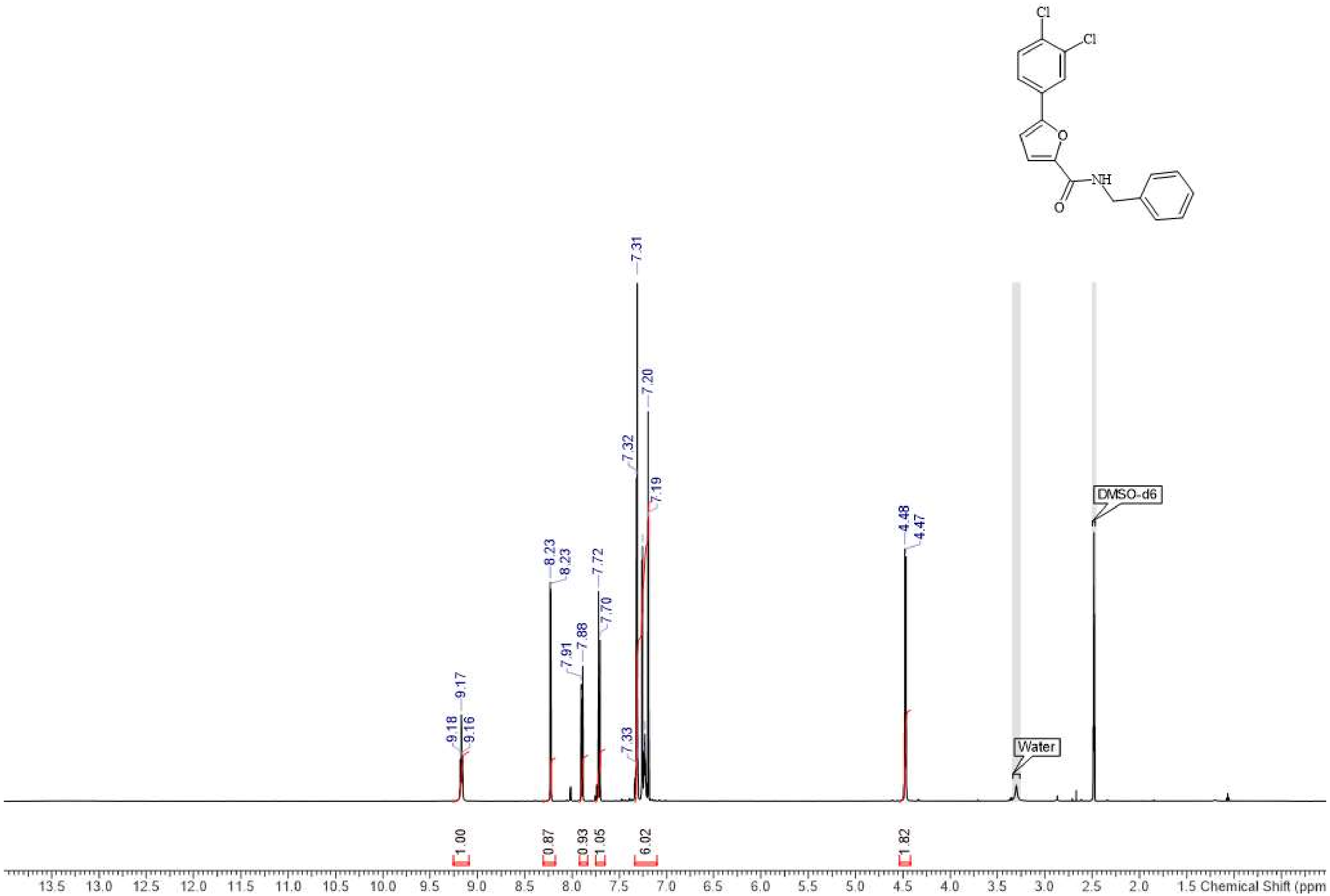

Figure S13: ^{1}H-NMR (500 MHz, DMSO-d_6) spectrum of N-benzyl-5-(3,4-dichlorophenyl)furan-2-carboxamide (6)

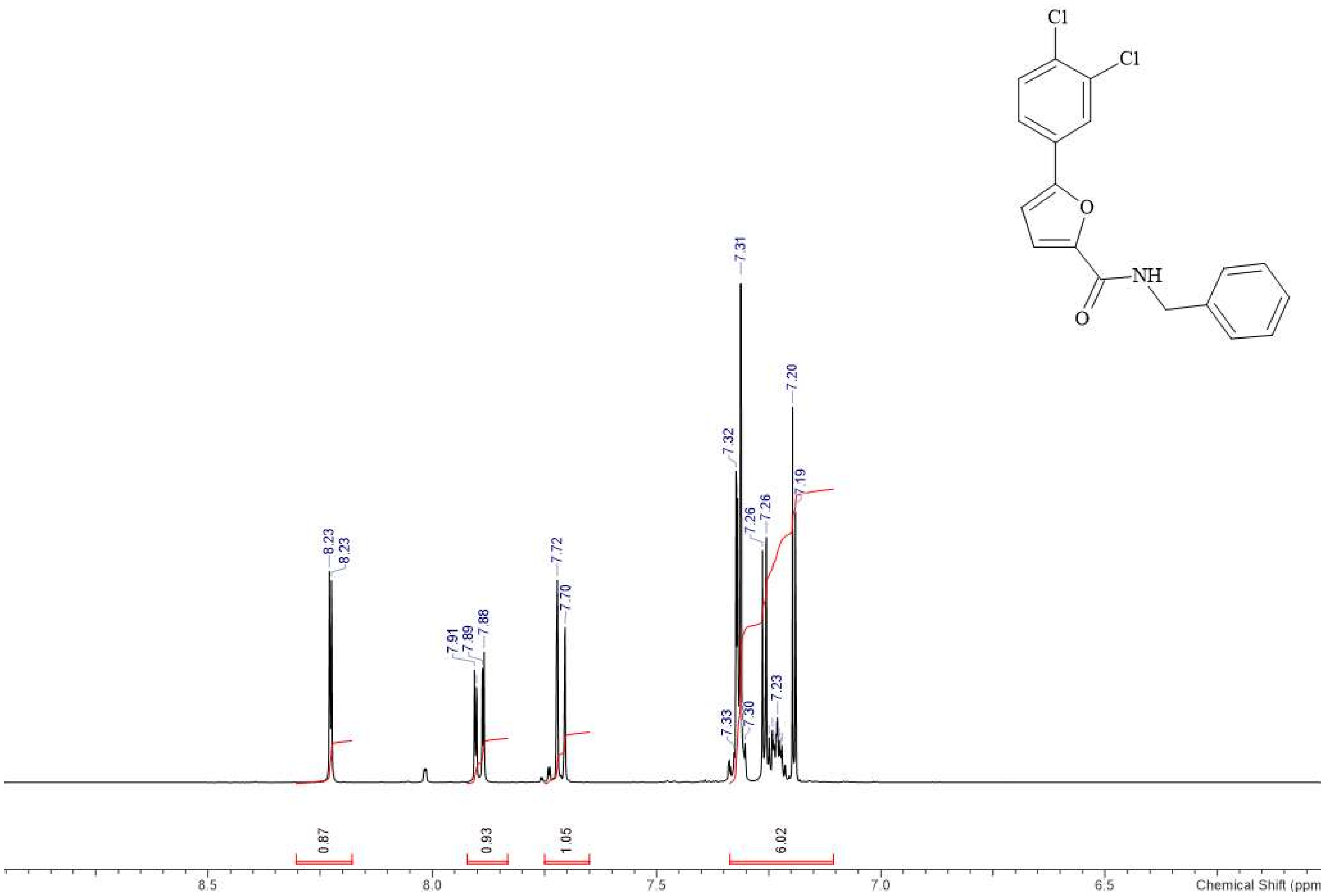

Figure S14: ^{1}H-NMR (500 MHz, DMSO-d_6) zoom of spectrum of N-benzyl-5-(3,4-dichlorophenyl)furan-2-carboxamide (6)

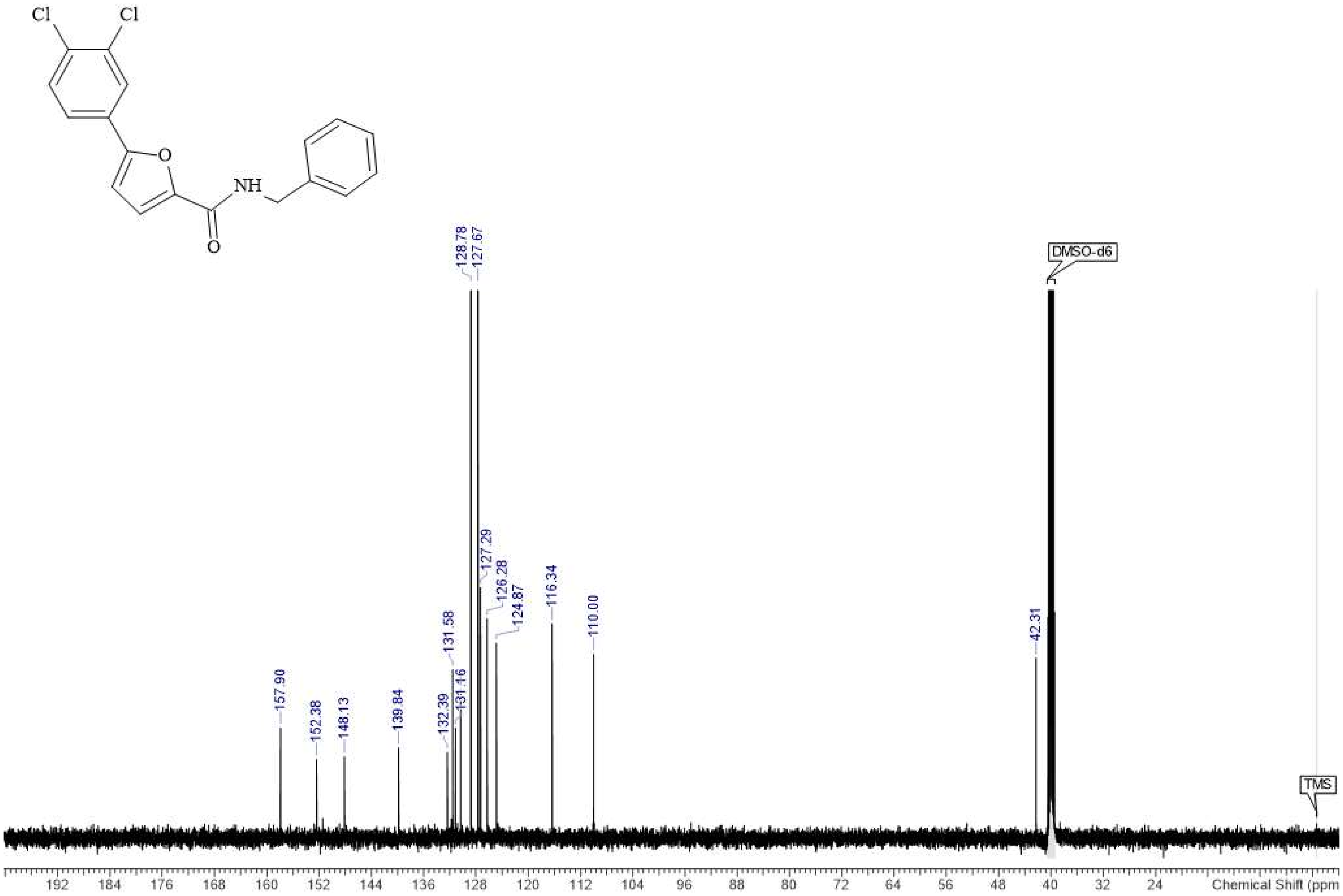

Figure S15: ^{13}C-NMR (126 MHz, DMSO-d$_6$) spectrum of N-benzyl-5-(3,4-dichlorophenyl)furan-2-carboxamide (6)

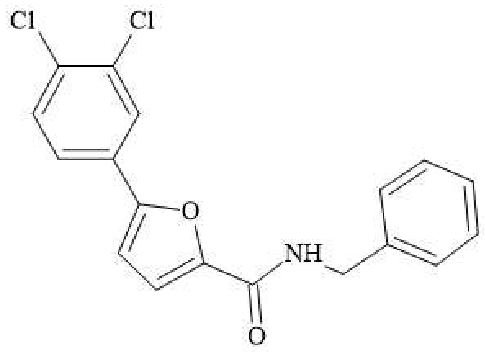

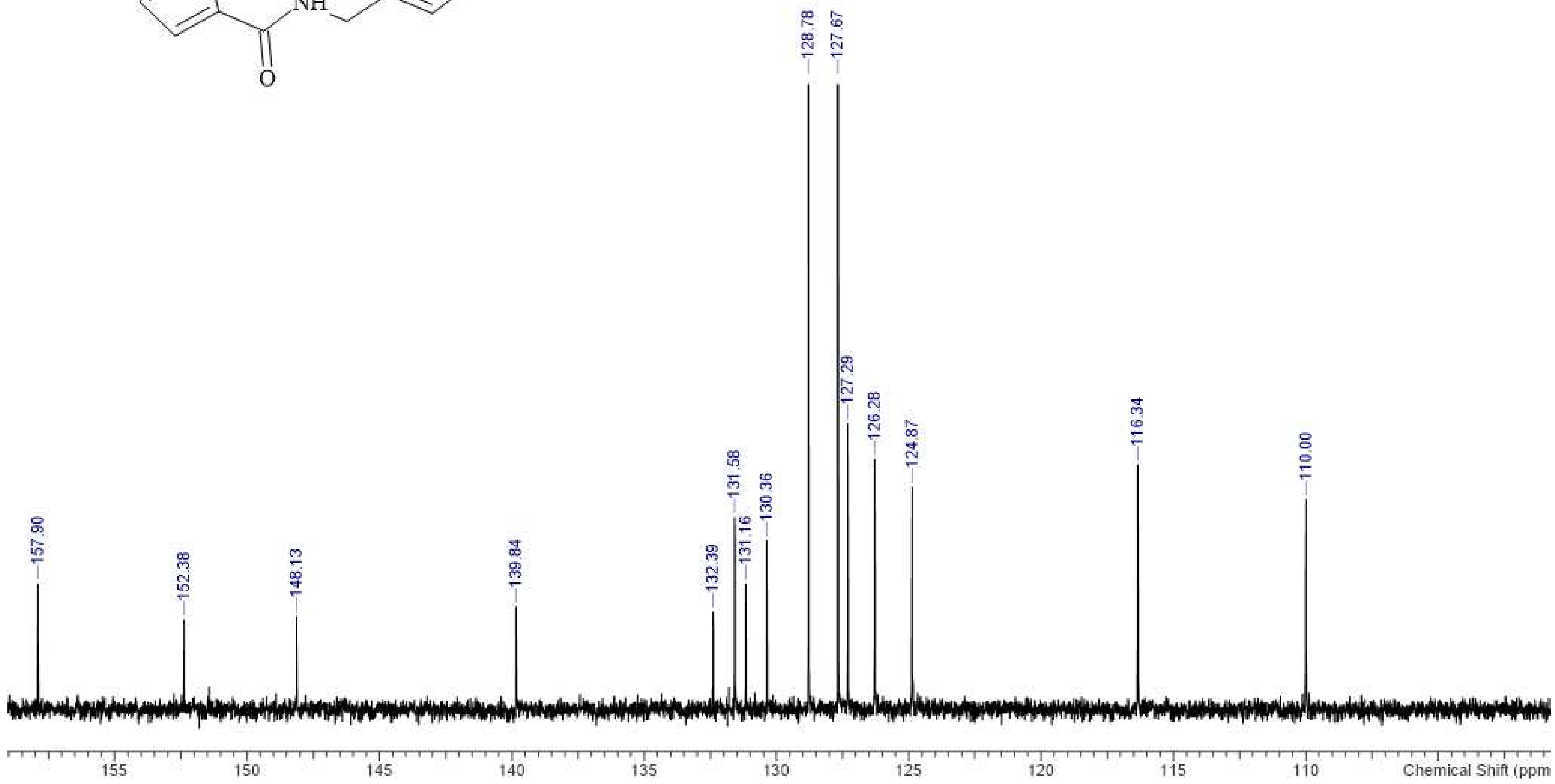

Figure S16: ^{13}C-NMR (126 MHz, DMSO-d$_6$) zoom of spectrum of N-benzyl-5-(3,4-dichlorophenyl)furan-2-carboxamide (6)

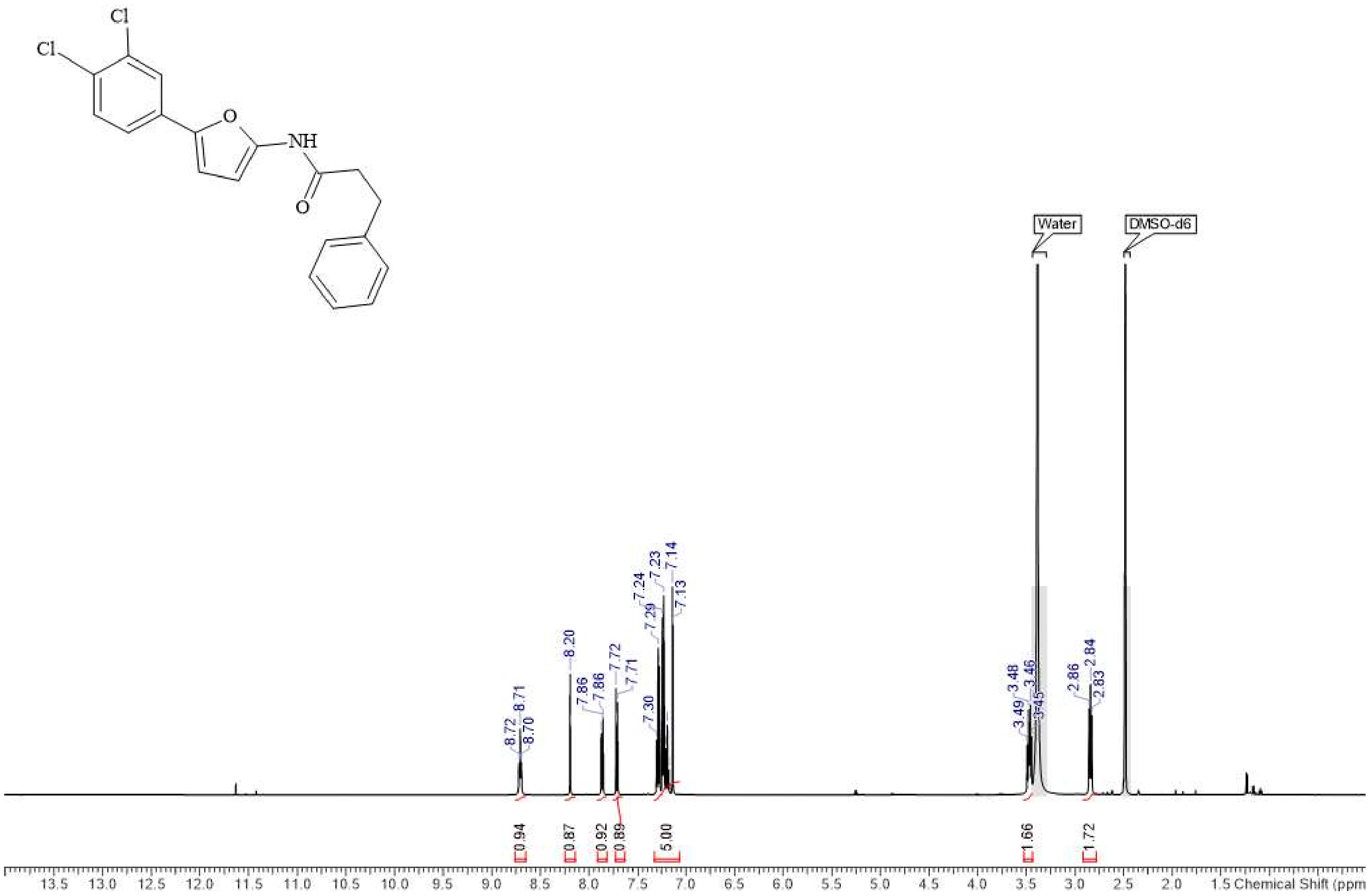

Figure S17: ^{1}H-NMR (500 MHz, DMSO-d_6) spectrum of 5-(3,4-dichlorophenyl)-*N*-(2-phenylethyl)furan-2-carboxamide (7)

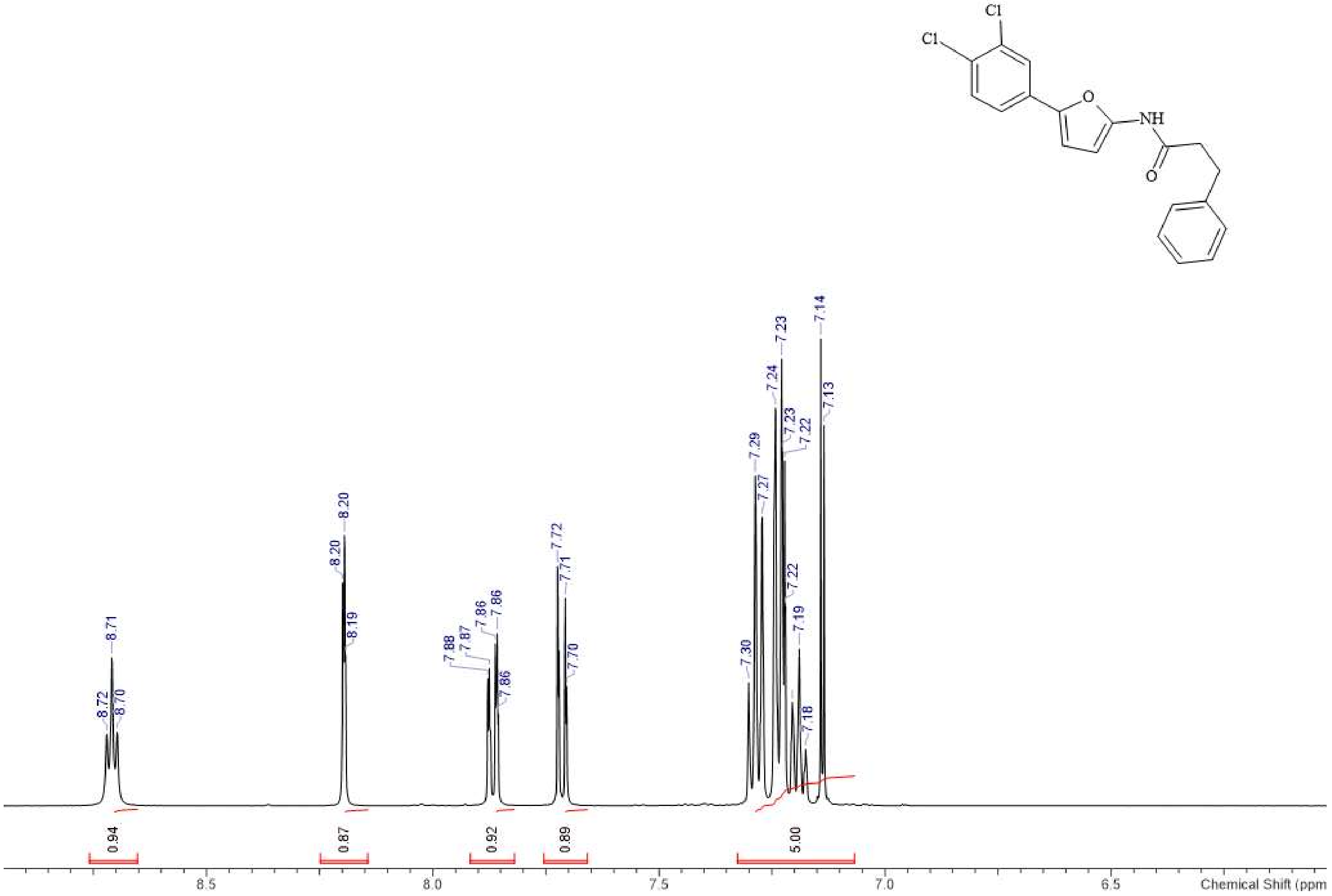

Figure S18: ^{1}H-NMR (500 MHz, DMSO-d_6) zoom of spectrum of 5-(3,4-dichlorophenyl)-*N*-(2-phenylethyl)furan-2-carboxamide (7)

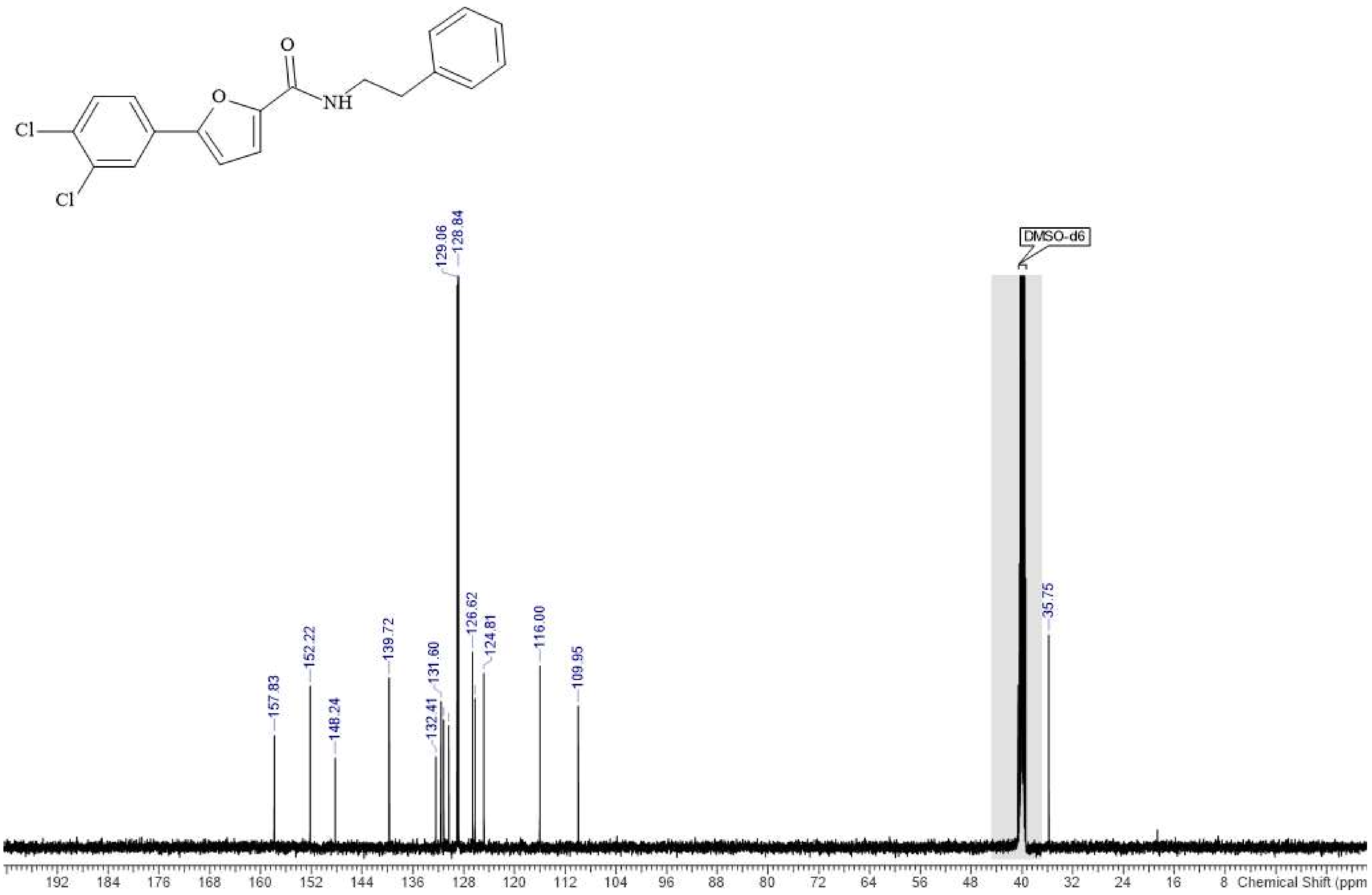

Figure S19: ^{13}C-NMR (126 MHz, DMSO-d_6) spectrum of 5-(3,4-dichlorophenyl)-*N*-(2-phenylethyl)furan-2-carboxamide (7)

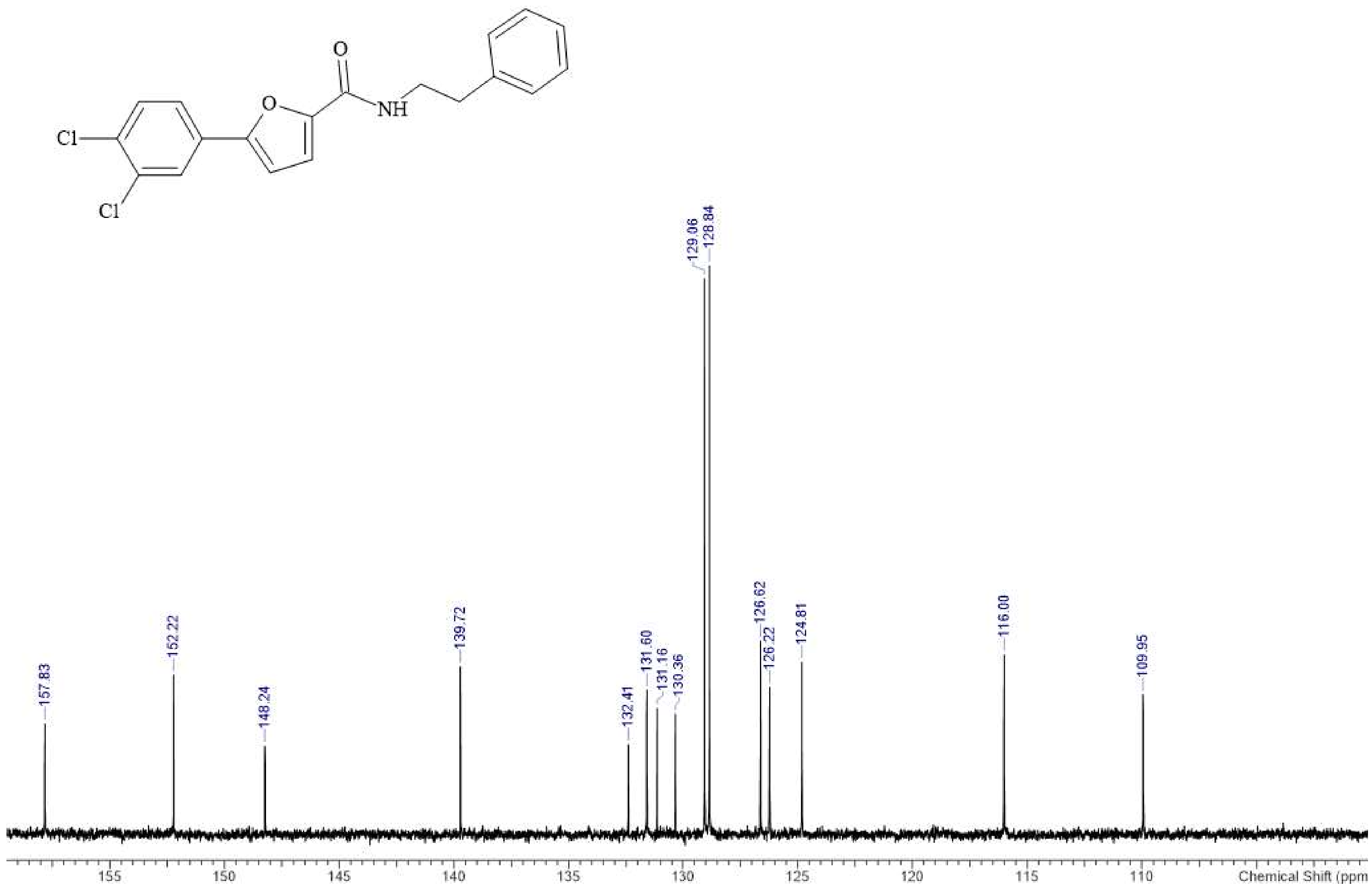

Figure S20: ^{13}C-NMR (126 MHz, DMSO-d_6) zoom of spectrum of 5-(3,4-dichlorophenyl)-*N*-(2-phenylethyl)furan-2-carboxamide (7)

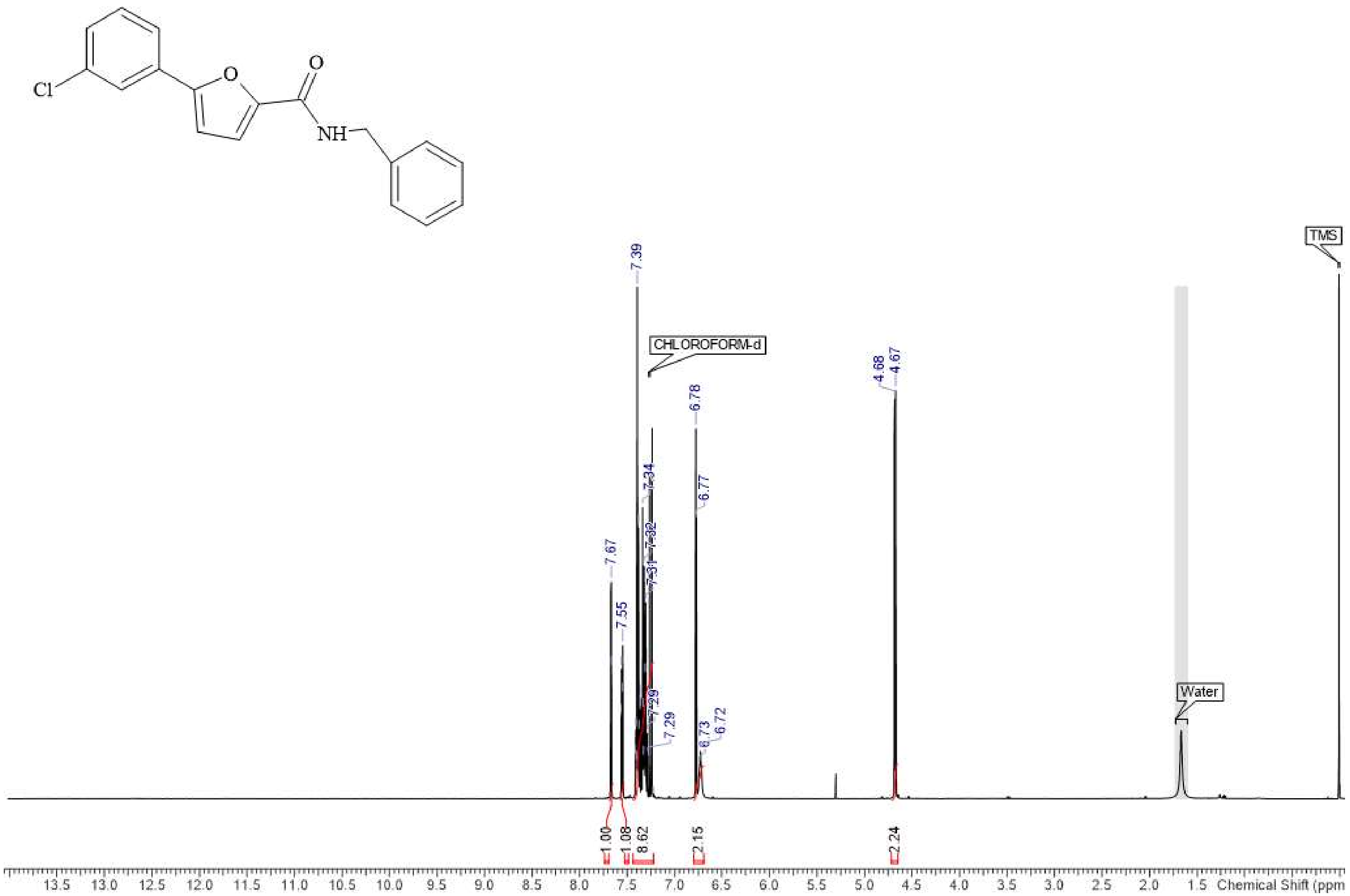

Figure S21: ^{1}H-NMR (500 MHz, $CDCl_3$) spectrum of *N*-benzyl-5-(3-chlorophenyl)furan-2-carboxamide (8)

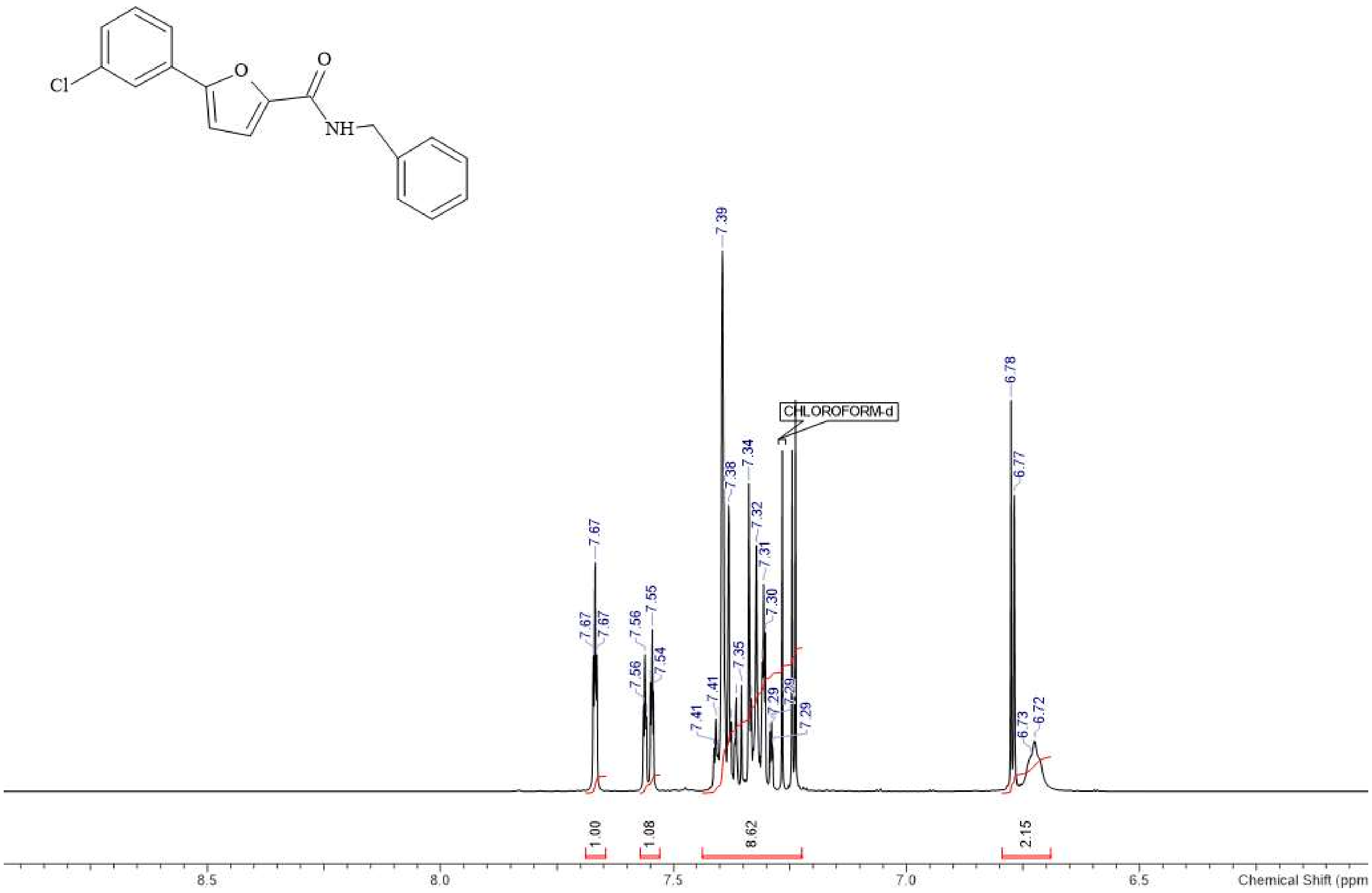

Figure S22: 1H-NMR (500 MHz, $CDCl_3$) zoom of spectrum of *N*-benzyl-5-(3-chlorophenyl)furan-2-carboxamide (8)

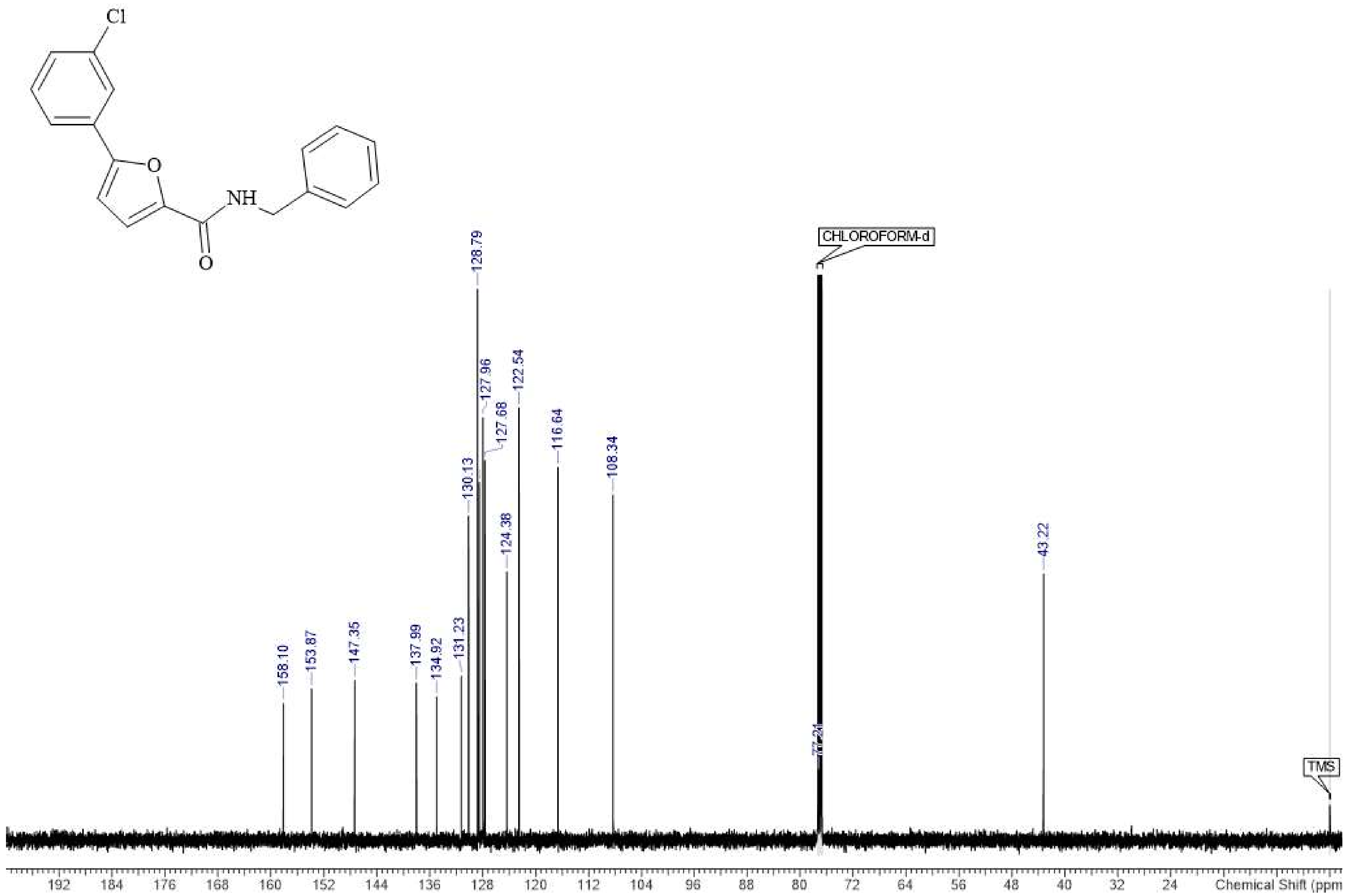

Figure S23: ^{13}C-NMR (126 MHz, $CDCl_3$) spectrum of *N*-benzyl-5-(3-chlorophenyl)furan-2-carboxamide (8)

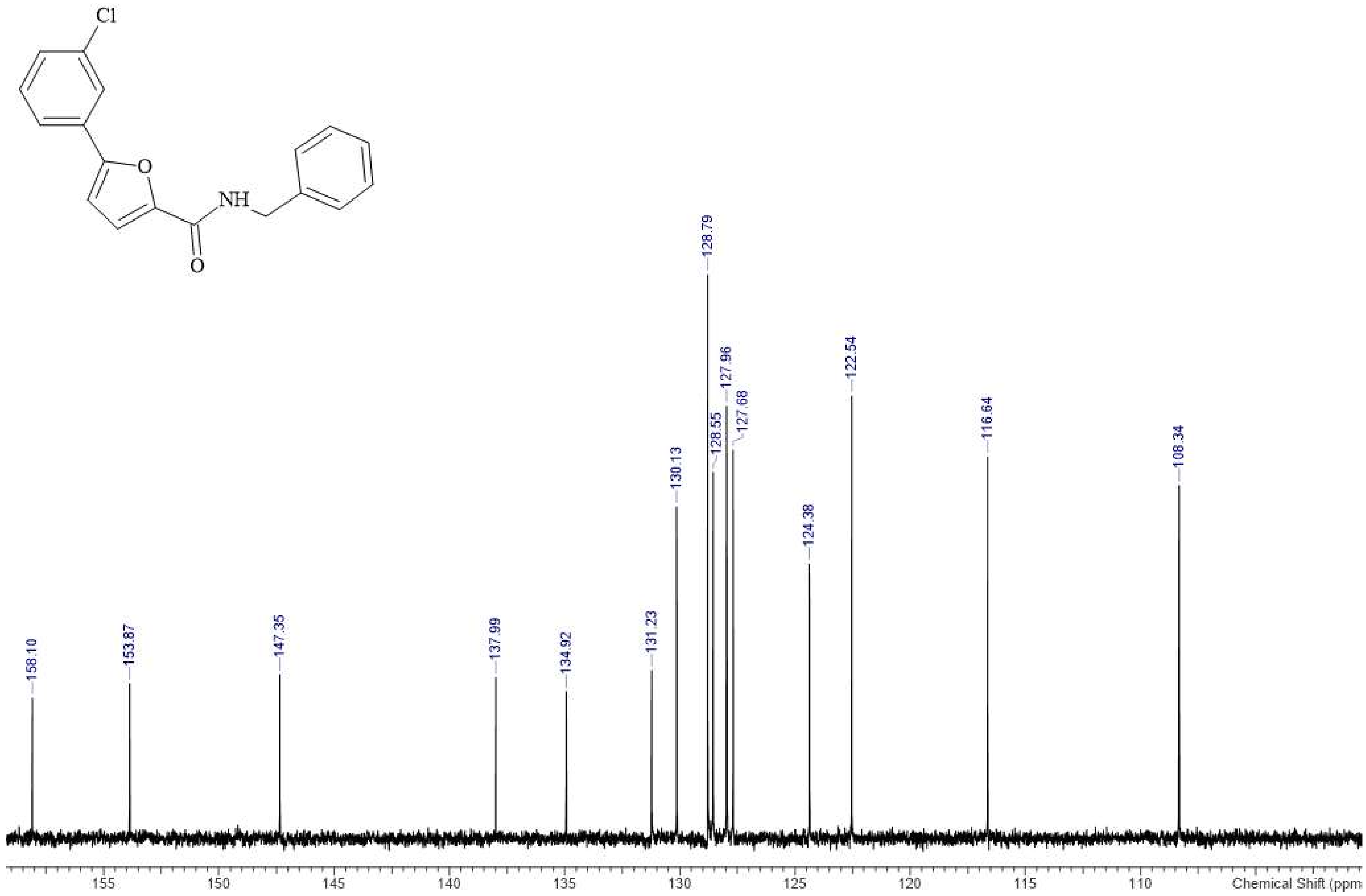

Figure S24: ^{13}C-NMR (126 MHz, $CDCl_3$) zoom of spectrum of *N*-benzyl-5-(3-chlorophenyl)furan-2-carboxamide (8)

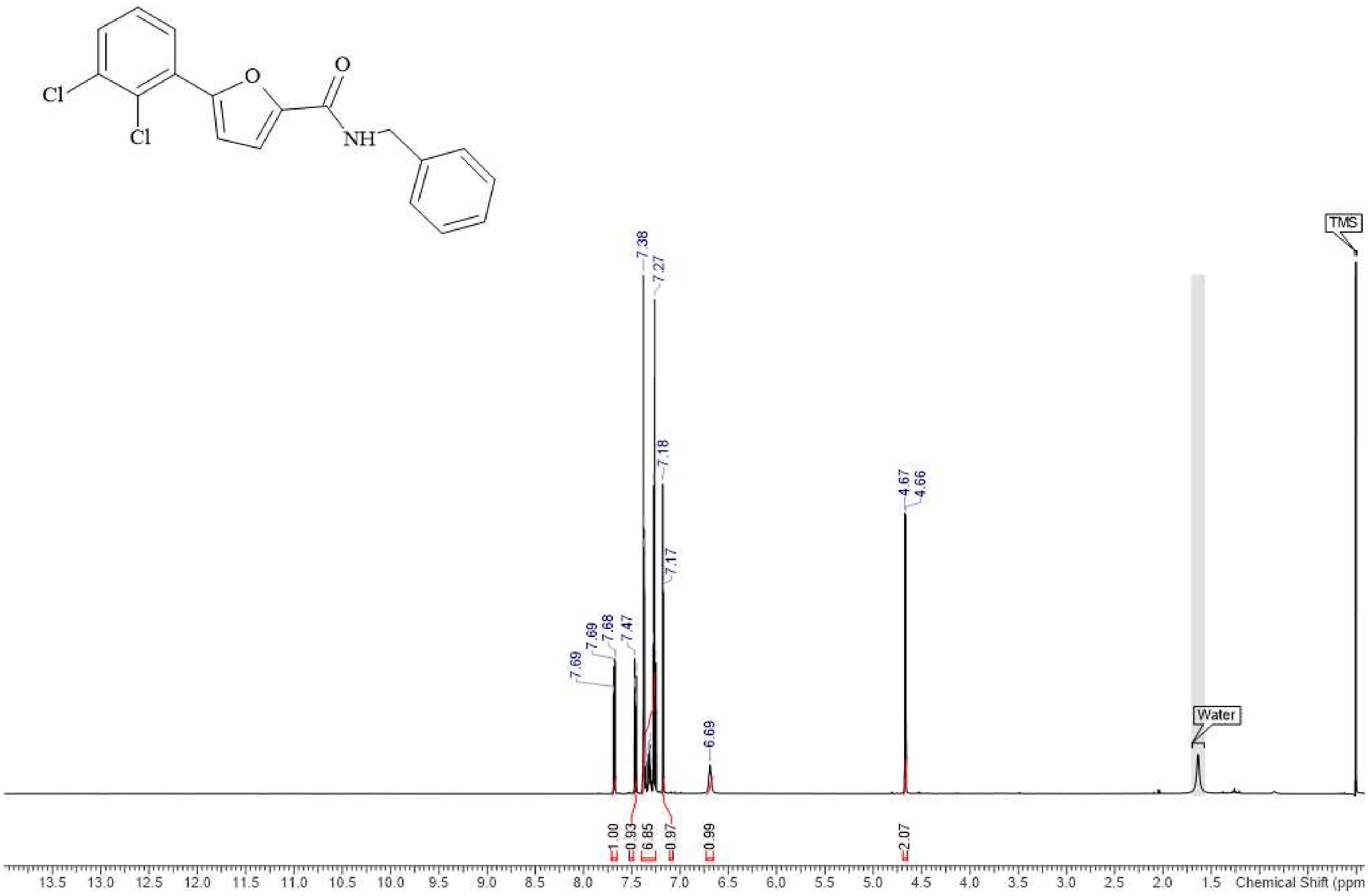

Figure S25: ^{1}H-NMR (500 MHz, $CDCl_3$) spectrum of *N*-benzyl-5-(2,3-dichlorophenyl)furan-2-carboxamide (9)

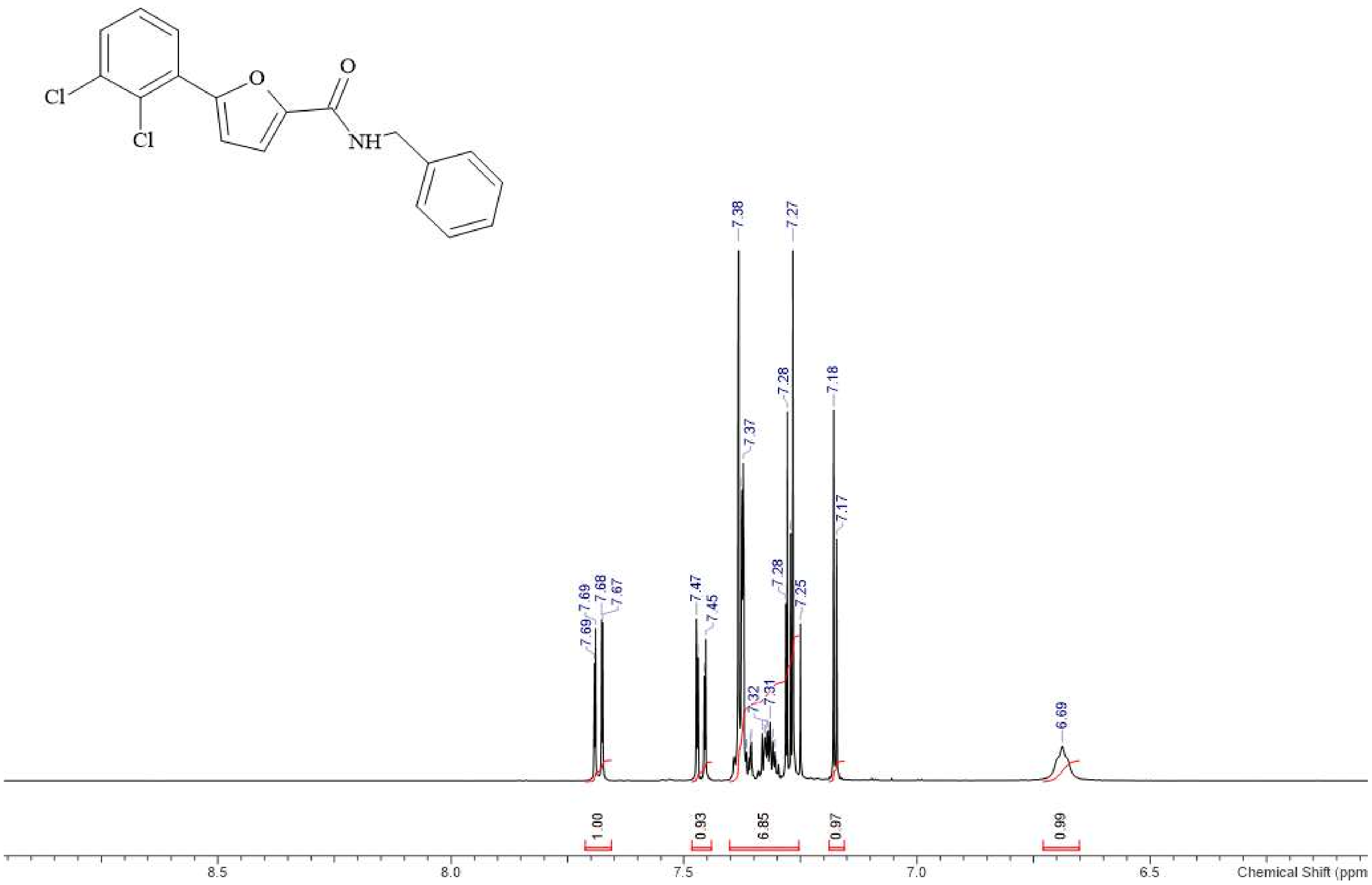

Figure S26: ^{1}H-NMR (500 MHz, $CDCl_3$) zoom of spectrum of *N*-benzyl-5-(2,3-dichlorophenyl)furan-2-carboxamide (9)

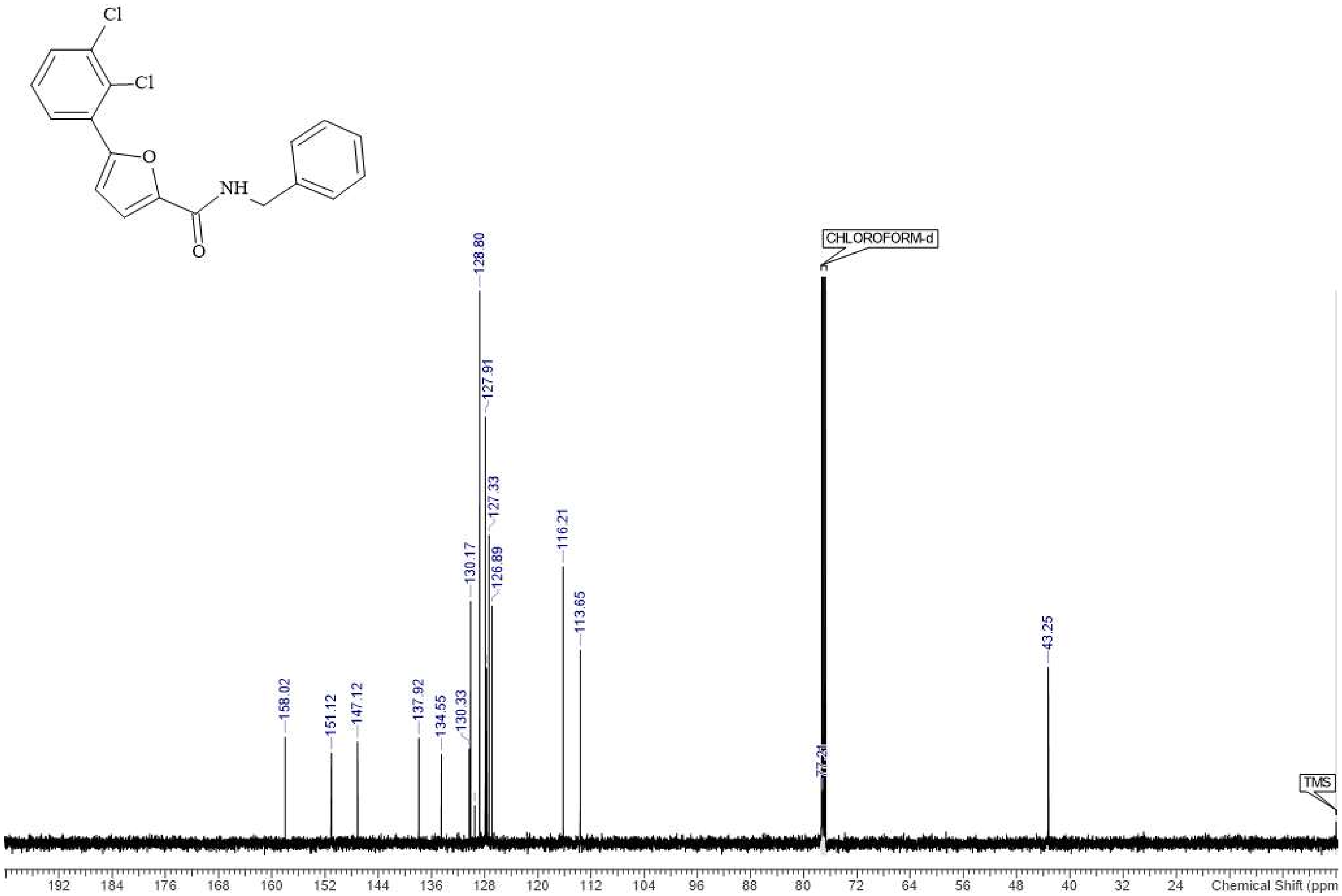

Figure S27: ^{13}C-NMR (126 MHz, $CDCl_3$) spectrum of *N*-Benzyl-5-(2,3-dichlorophenyl)furan-2-carboxamide (9)

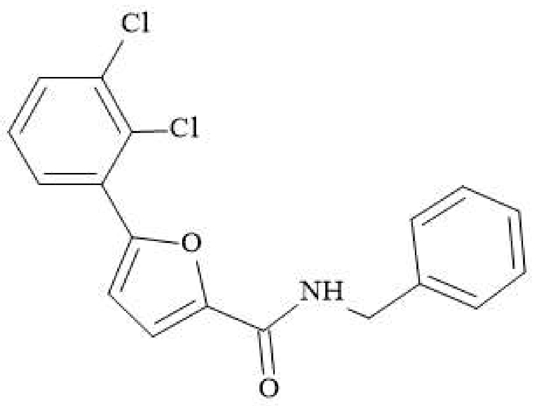

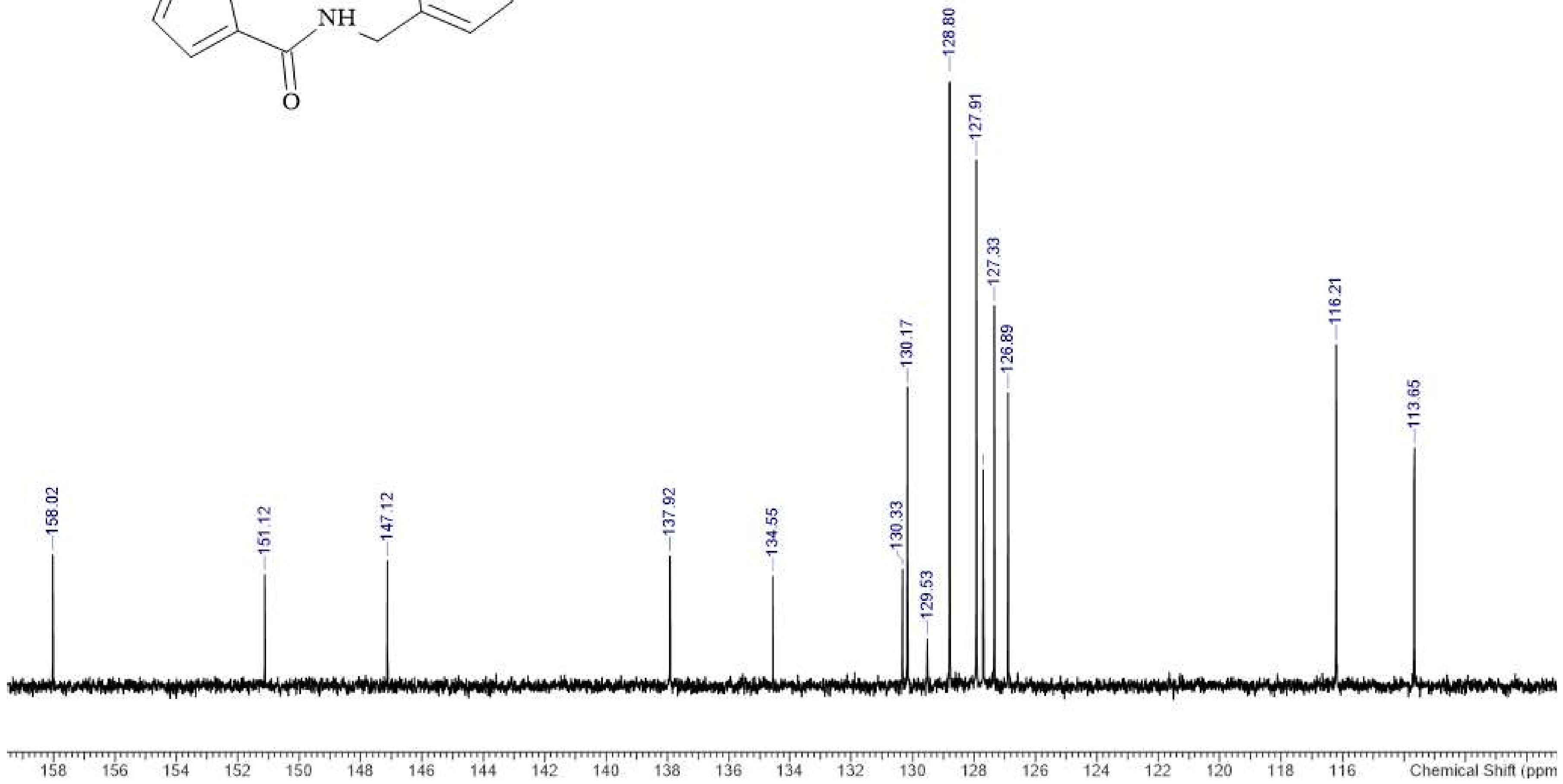

Figure S28: ^{13}C-NMR (126 MHz, $CDCl_3$) zoom of spectrum of *N*-Benzyl-5-(2,3-dichlorophenyl)furan-2-carboxamide (9)

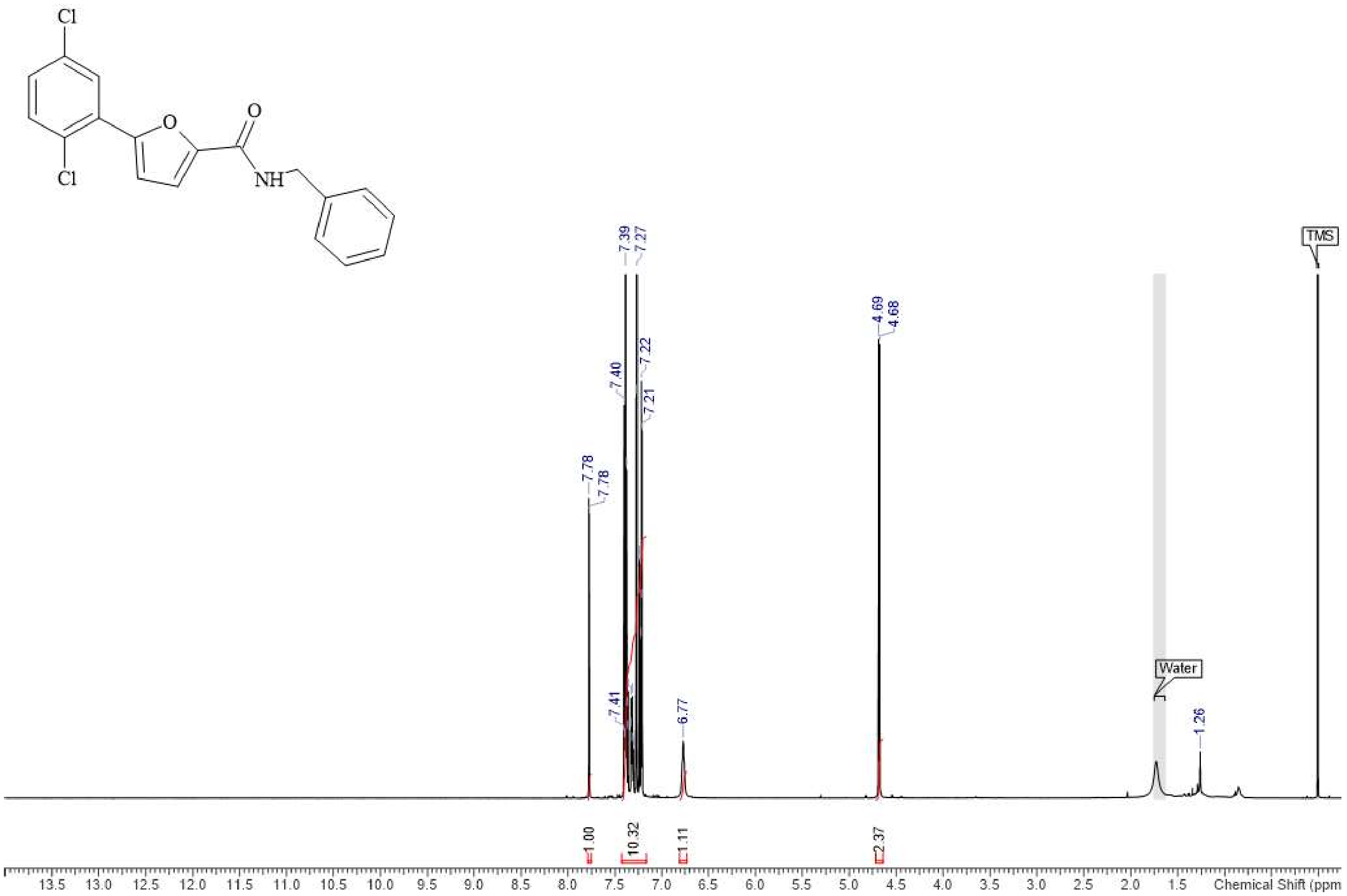

Figure S29: ^{1}H-NMR (500 MHz, CDCl$_3$) spectrum of *N*-benzyl-5-(2,5-dichlorophenyl)furan-2-carboxamide (10)

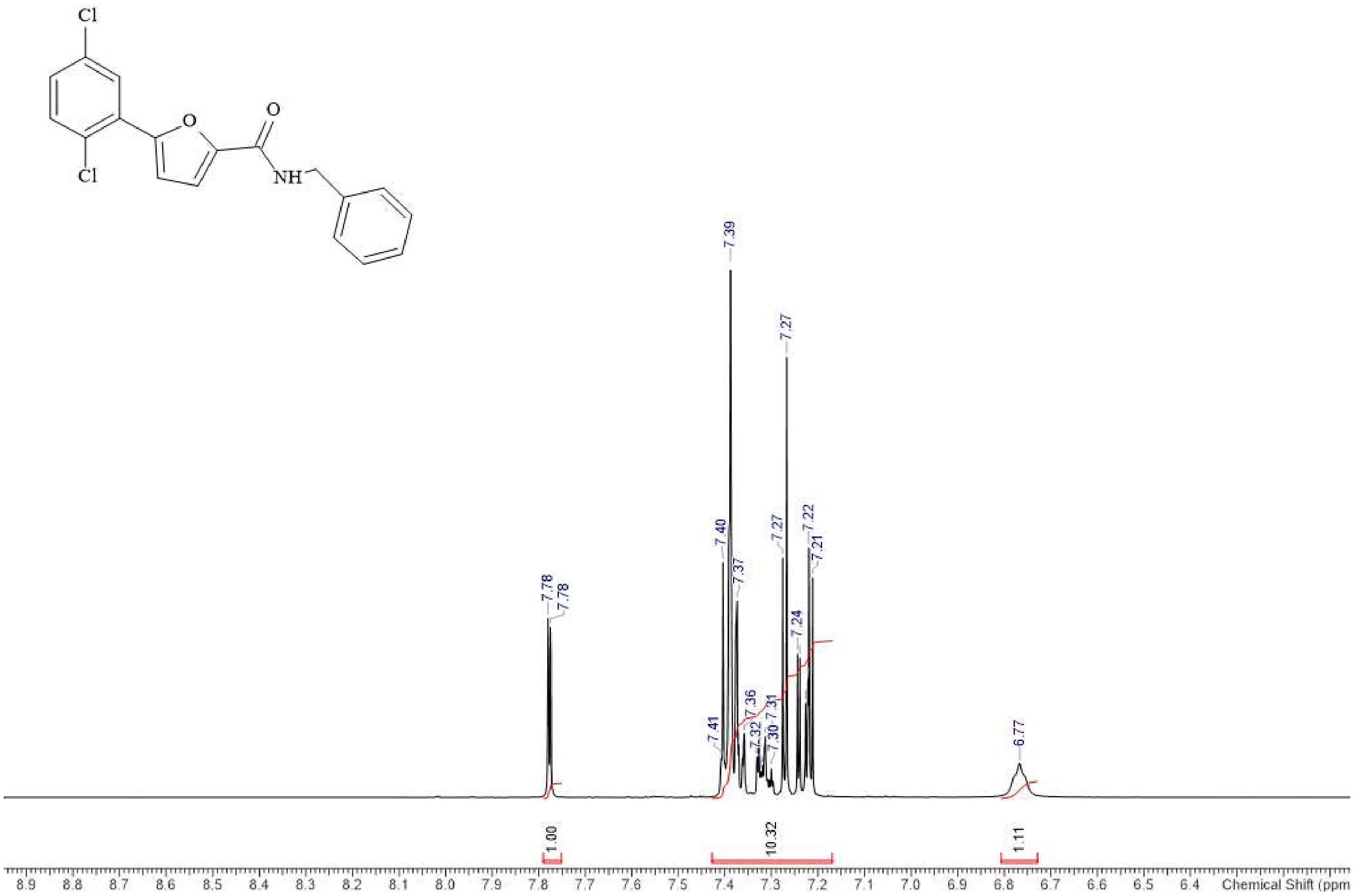

Figure S30: ^{1}H-NMR (500 MHz, $CDCl_3$) zoom of spectrum of *N*-benzyl-5-(2,5-dichlorophenyl)furan-2-carboxamide (10)

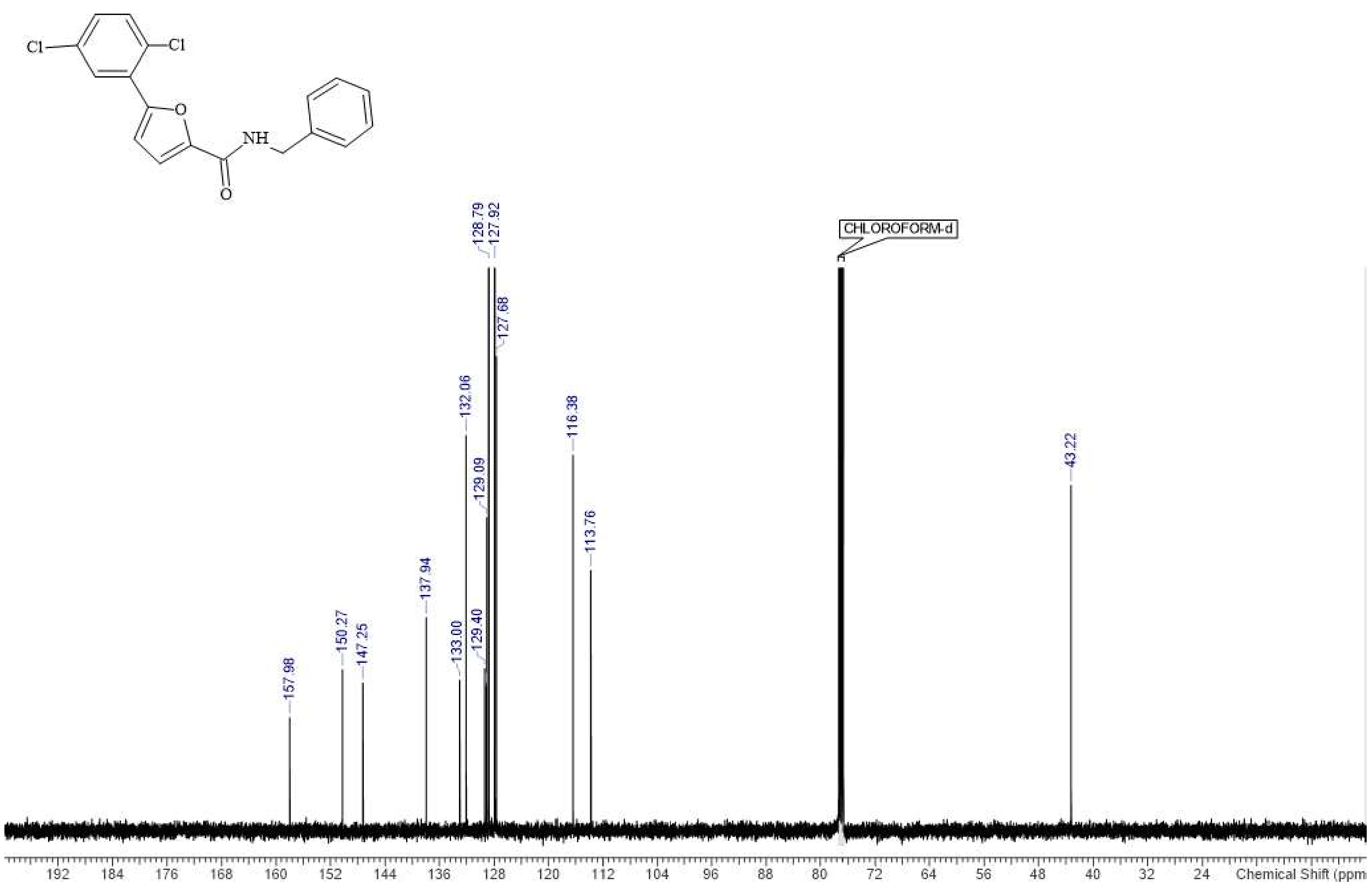

Figure S31: ^{13}C-NMR (126 MHz, $CDCl_3$) spectrum of *N*-benzyl-5-(2,5-dichlorophenyl)furan-2-carboxamide (10)

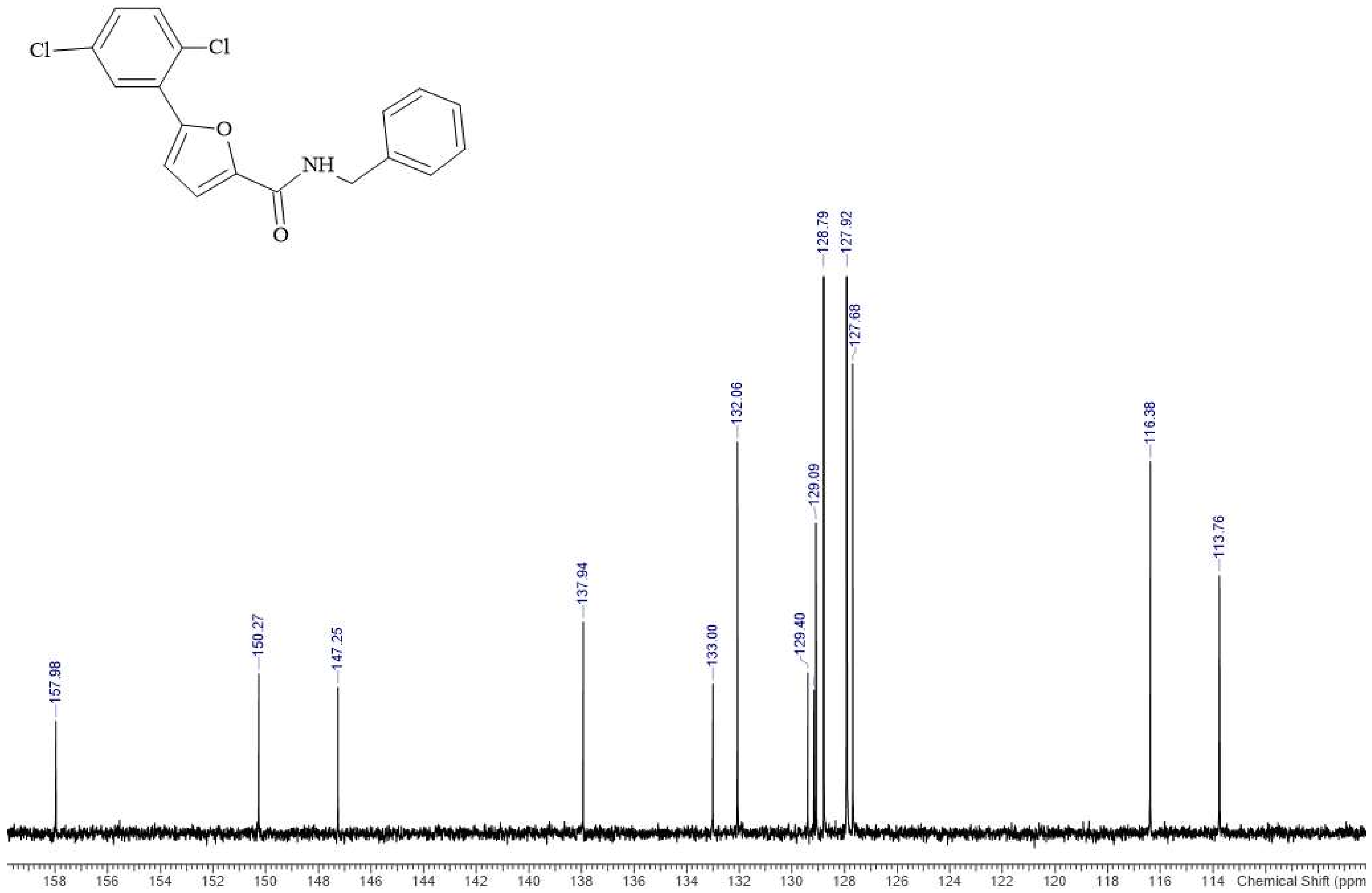

Figure S32: ^{13}C-NMR (126 MHz, $CDCl_3$) zoom of spectrum of *N*-benzyl-5-(2,5-dichlorophenyl)furan-2-carboxamide (10)

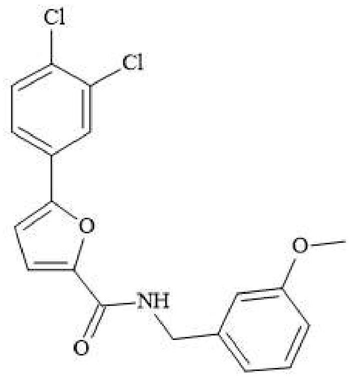

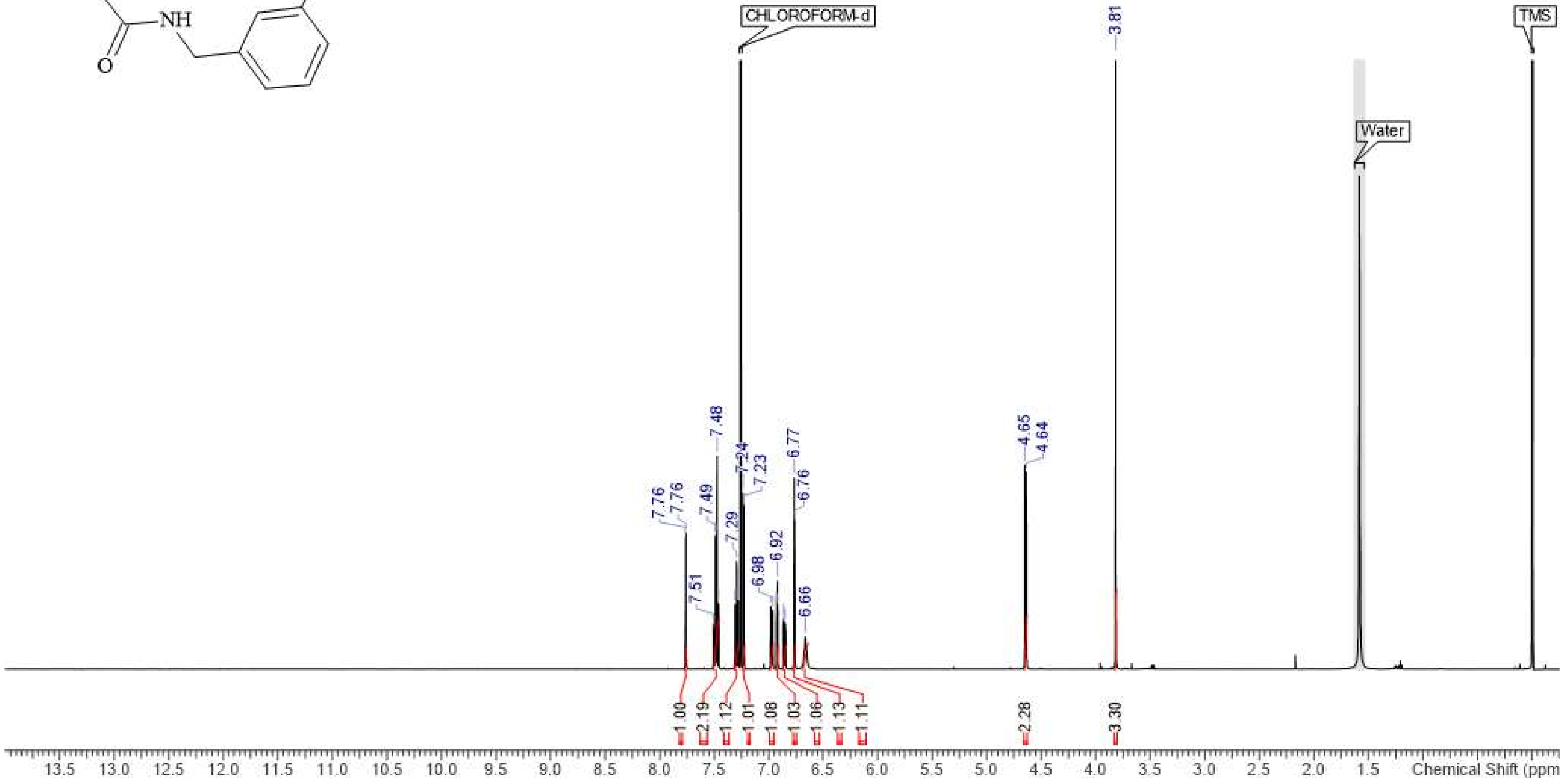

Figure S33: ^{1}H-NMR (500 MHz, $CDCl_3$) spectrum of 5-(3,4-dichlorophenyl)-*N*-(3-methoxyphenyl)furan-2-carboxamide (11)

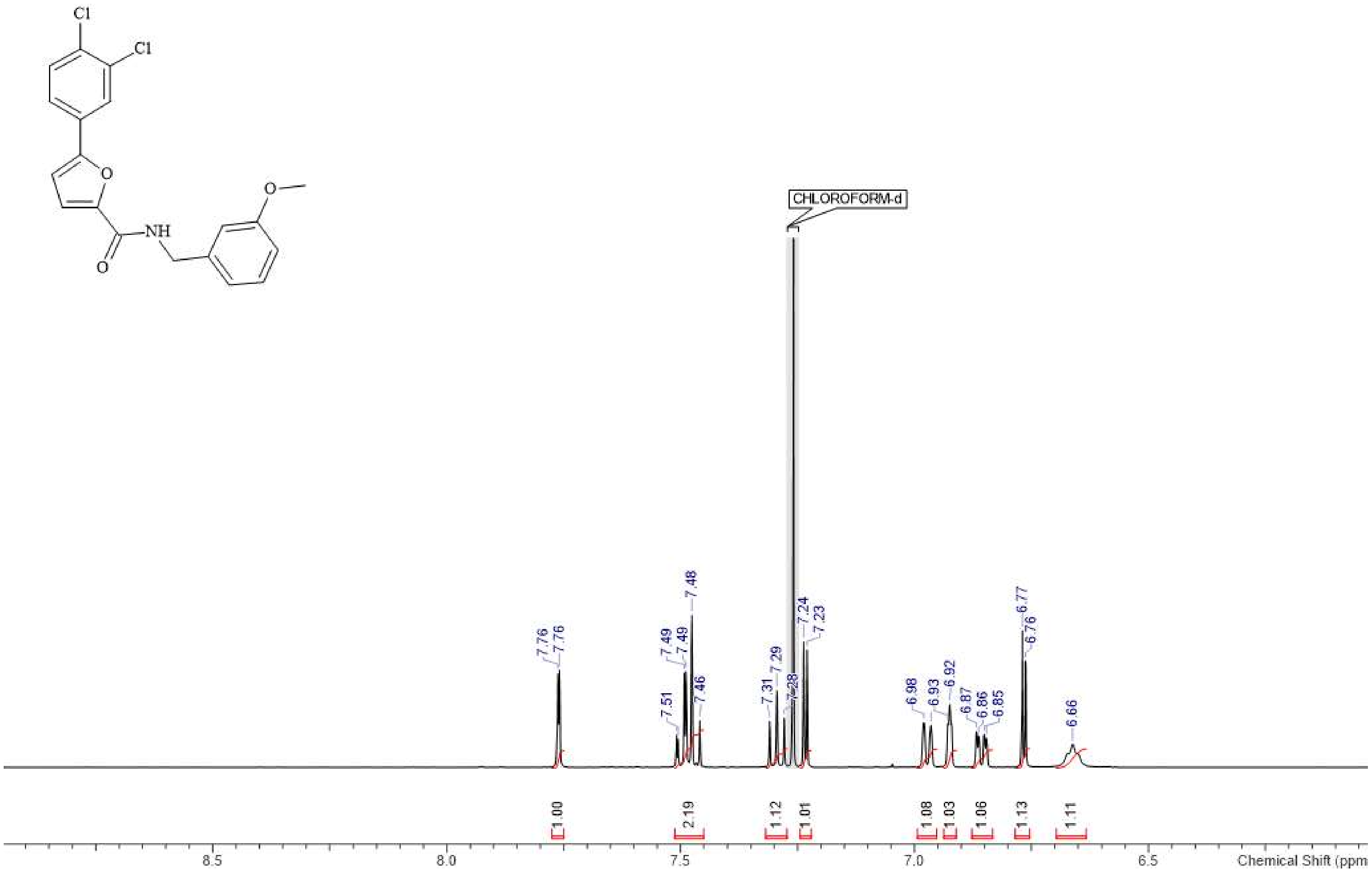

Figure S34: ^{1}H-NMR (500 MHz, $CDCl_3$) zoom of spectrum of 5-(3,4-dichlorophenyl)-*N*-(3-methoxyphenyl)furan-2-carboxamide (**11**)

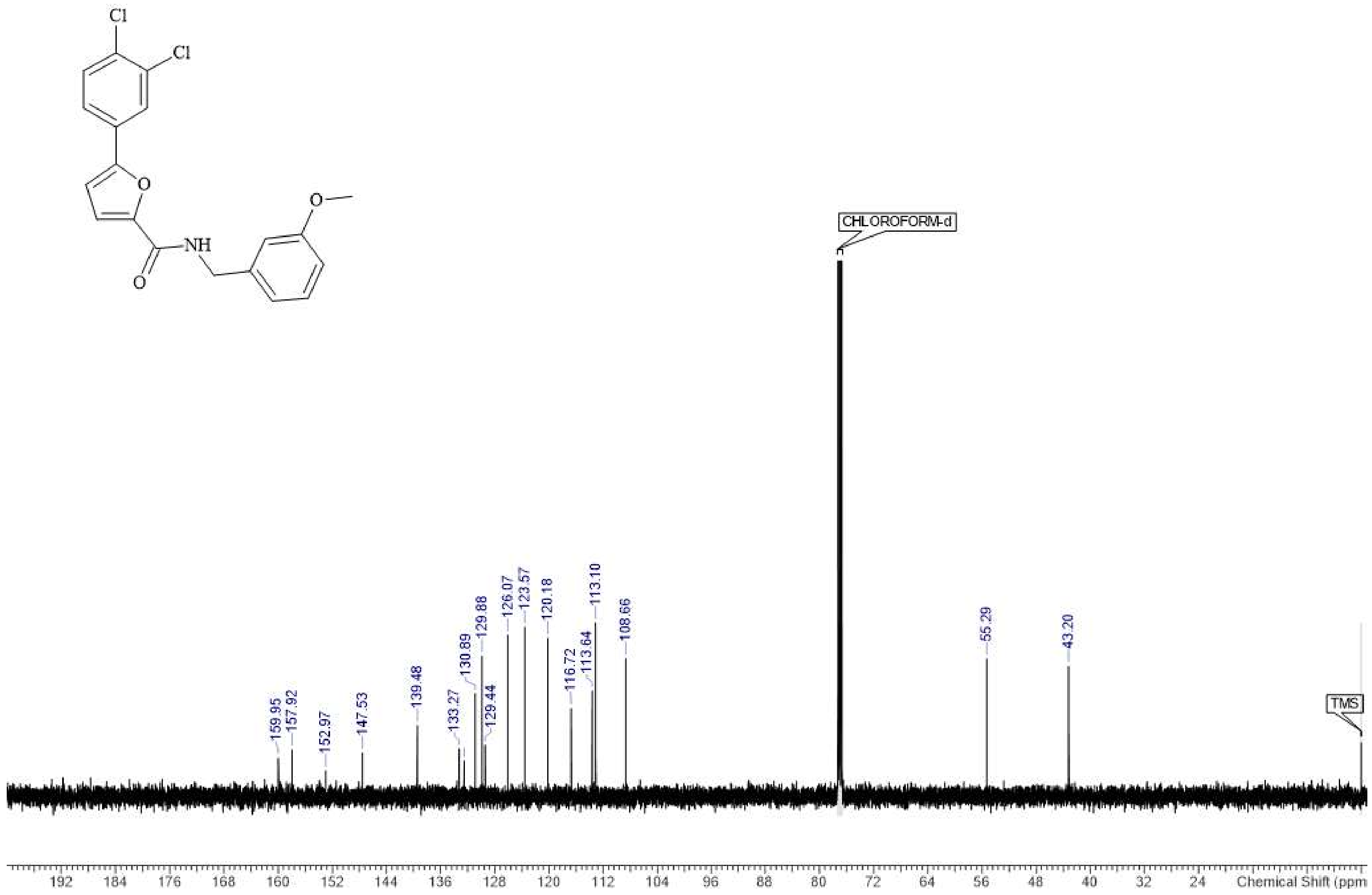

Figure S35: ^{13}C-NMR (126 MHz, $CDCl_3$) spectrum of 5-(3,4-dichlorophenyl)-*N*-(3-methoxyphenyl)furan-2-carboxamide (11)

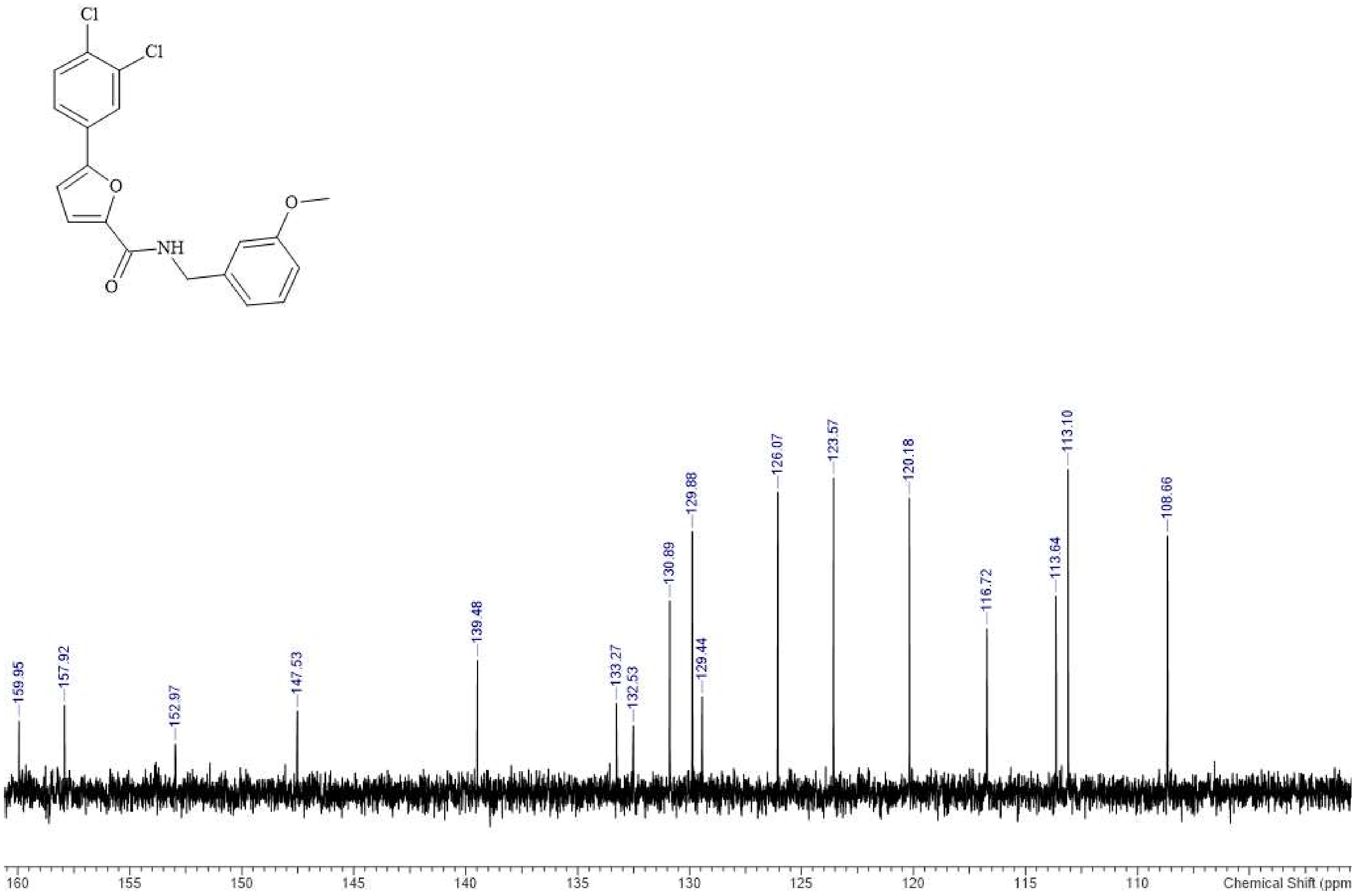

Figure S36: ^{13}C-NMR (126 MHz, $CDCl_3$) zoom of spectrum of 5-(3,4-dichlorophenyl)-*N*-(3-methoxyphenyl)furan-2-carboxamide (11)

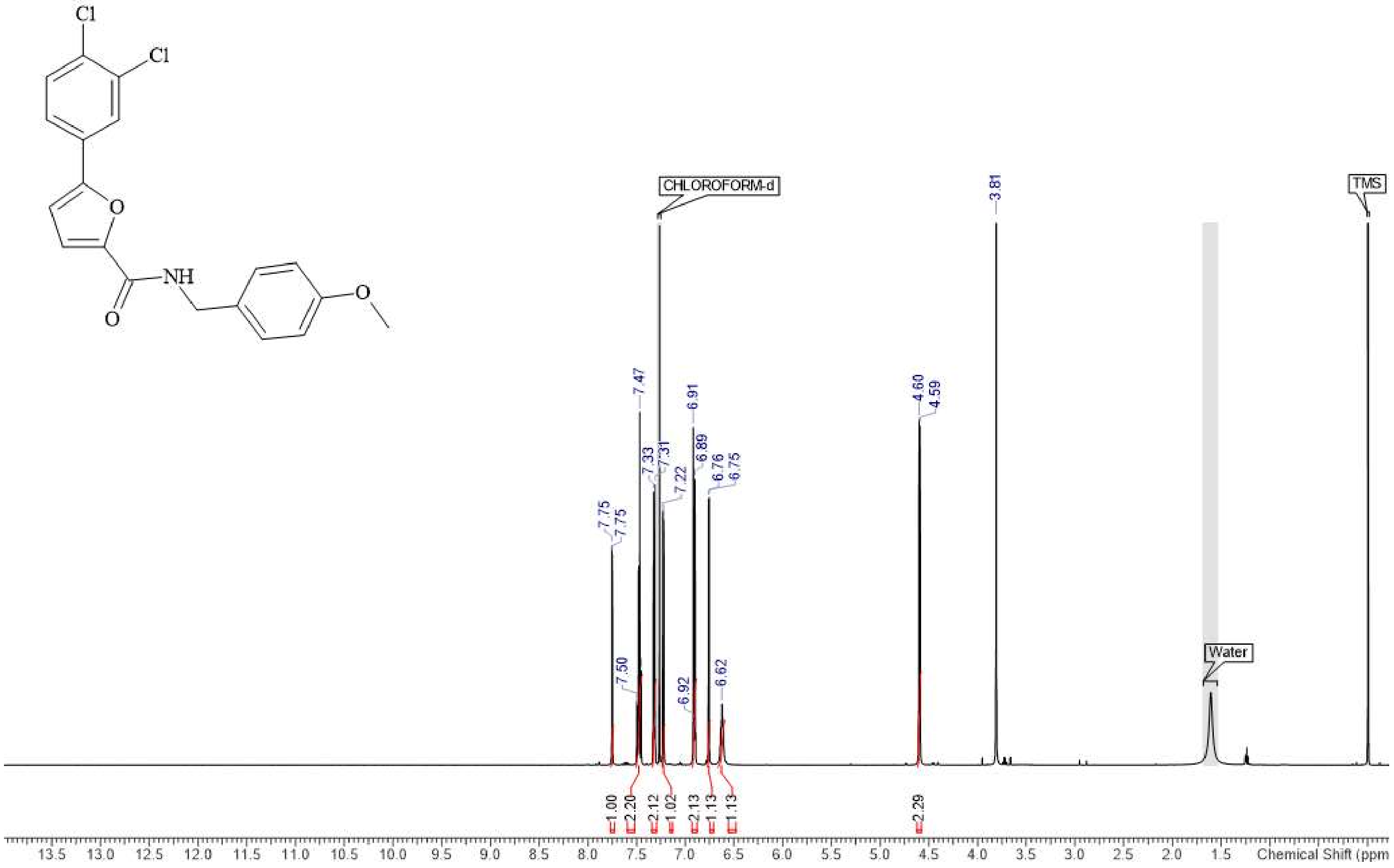

Figure S37: ^{1}H-NMR (500 MHz, $CDCl_3$) spectrum of 5-(3,4-dichlorophenyl)-*N*-(4-methoxybenzyl)furan-2-carboxamide (12)

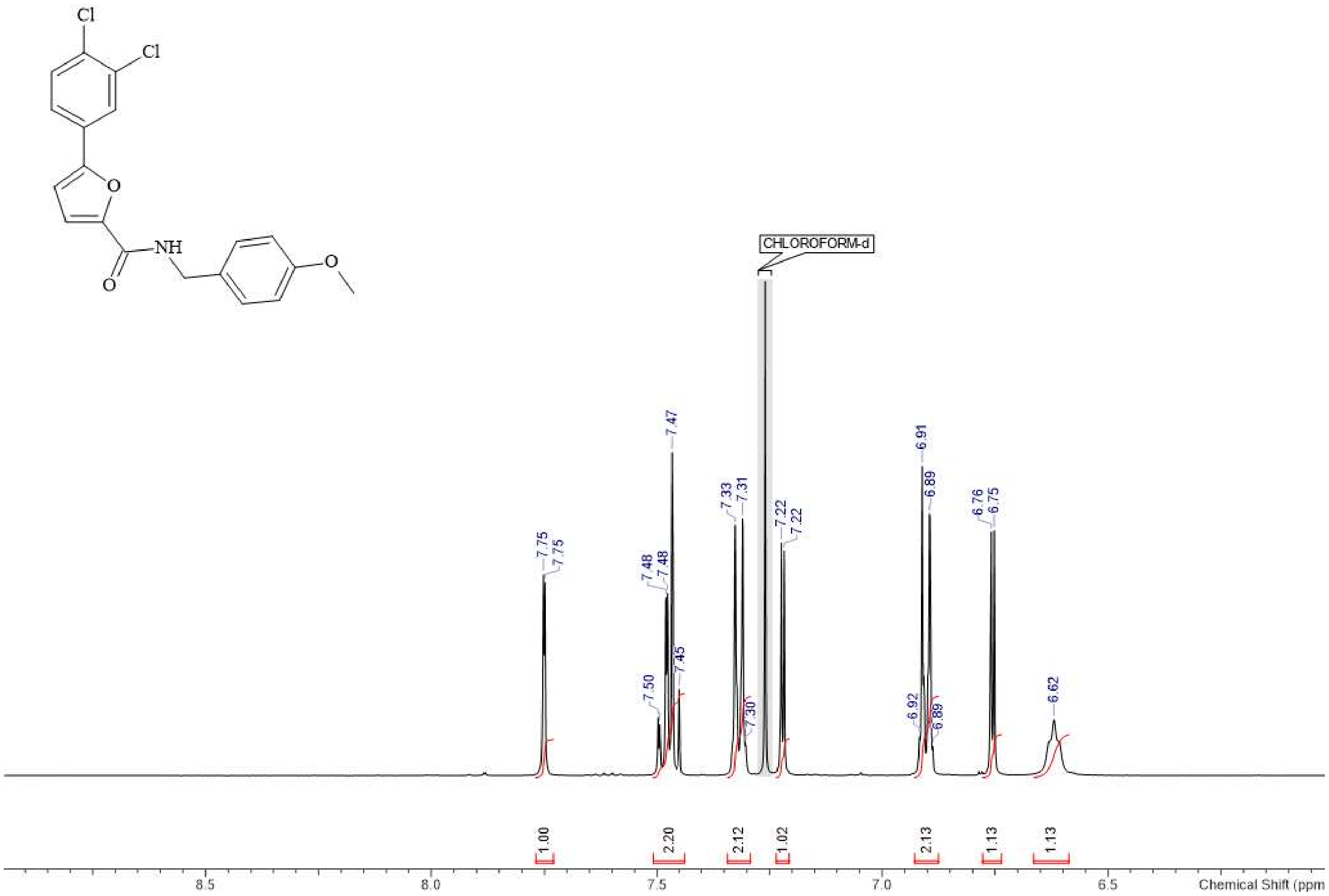

Figure S38: ^{1}H-NMR (500 MHz, $CDCl_3$) zoom of spectrum of 5-(3,4-dichlorophenyl)-*N*-(4-methoxybenzyl)furan-2-carboxamide (12)

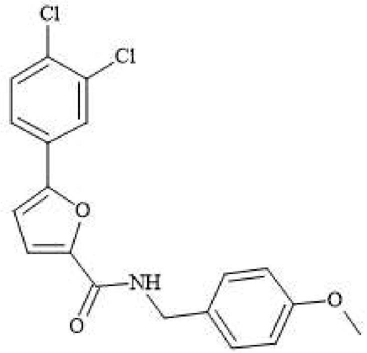

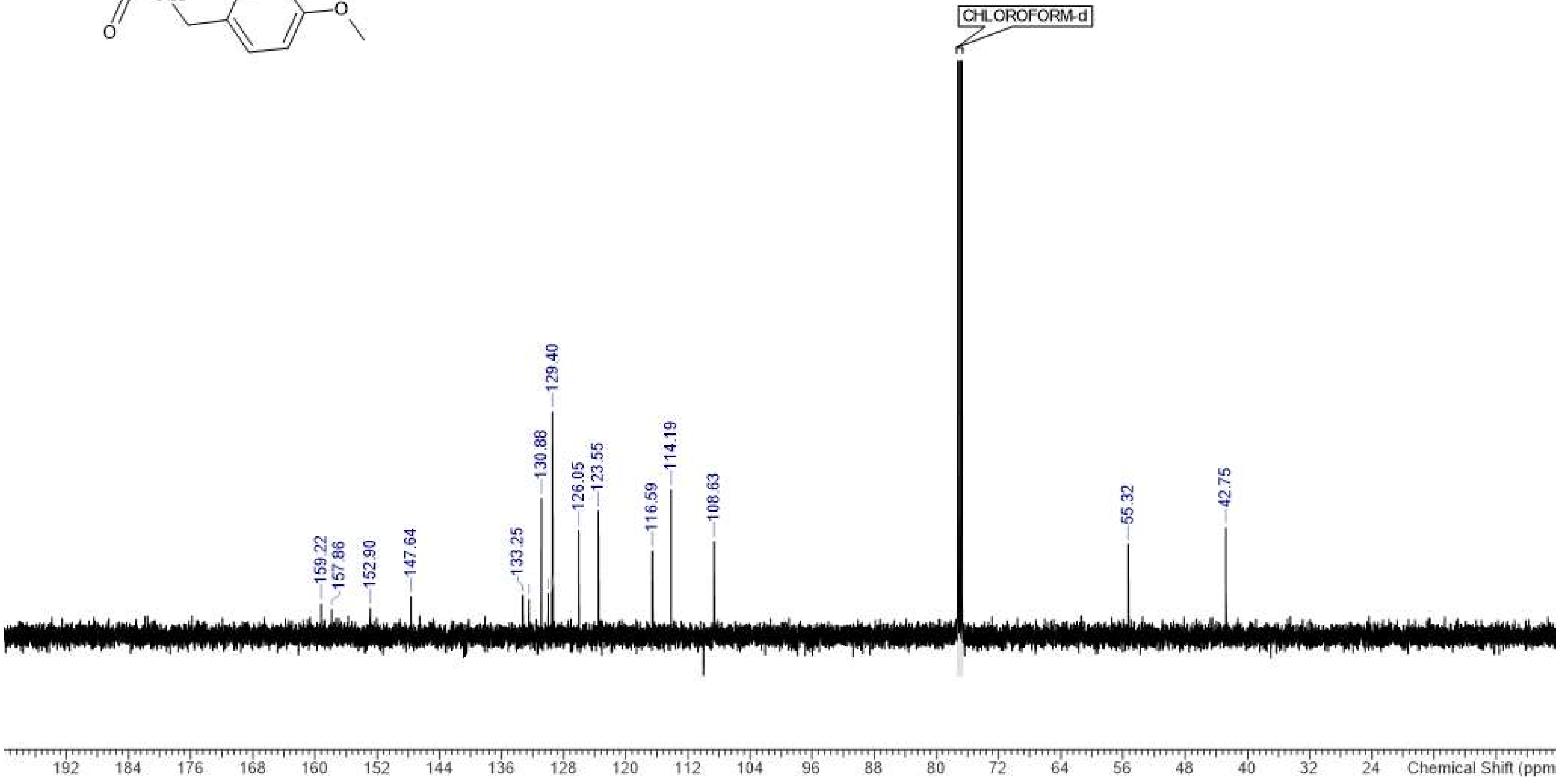

Figure S39: ^{13}C-NMR (126 MHz, $CDCl_3$) spectrum of 5-(3,4-dichlorophenyl)-*N*-(4-methoxybenzyl)furan-2-carboxamide (12)

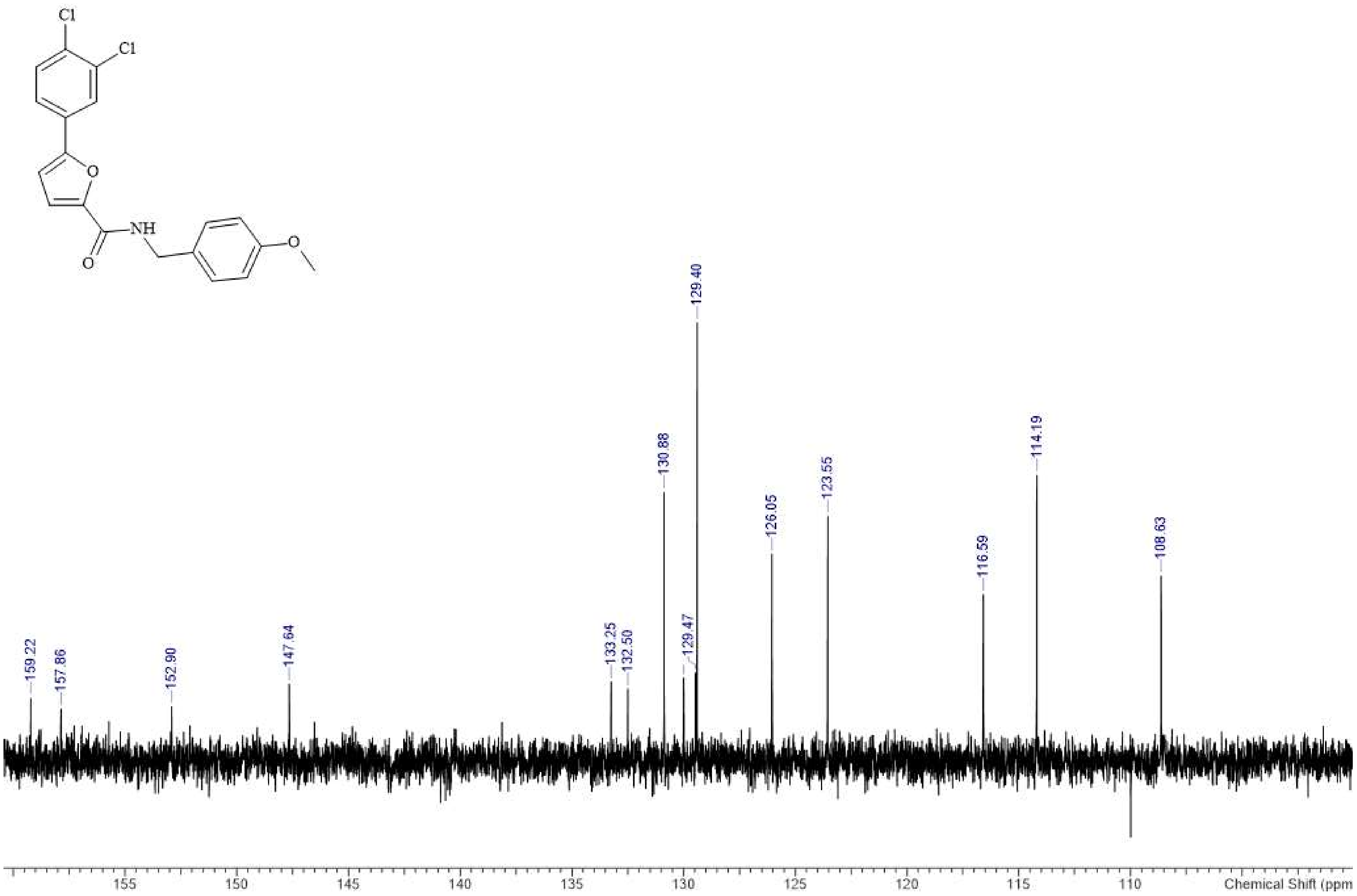

Figure S40: ^{13}C-NMR (126 MHz, $CDCl_3$) zoom of spectrum of 5-(3,4-dichlorophenyl)-*N*-(4-methoxybenzyl)furan-2-carboxamide (12)

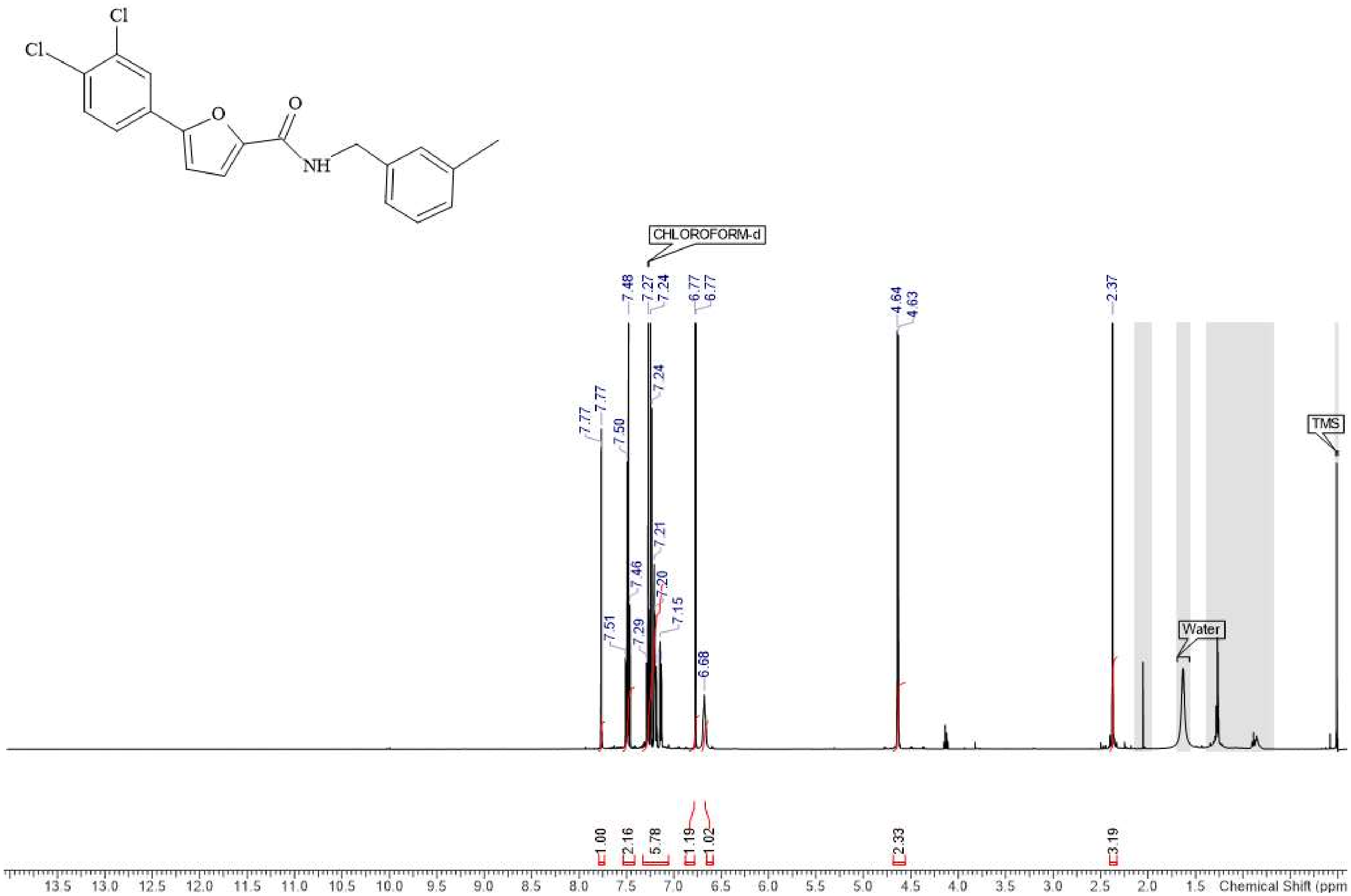

Figure S41: ^{1}H-NMR (500 MHz, $CDCl_3$) spectrum of 5-(3,4-dichlorophenyl)-*N*-(3-methylbenzyl)furan-2-carboxamide (13)

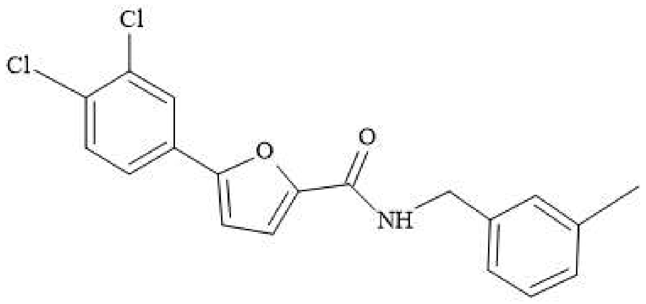

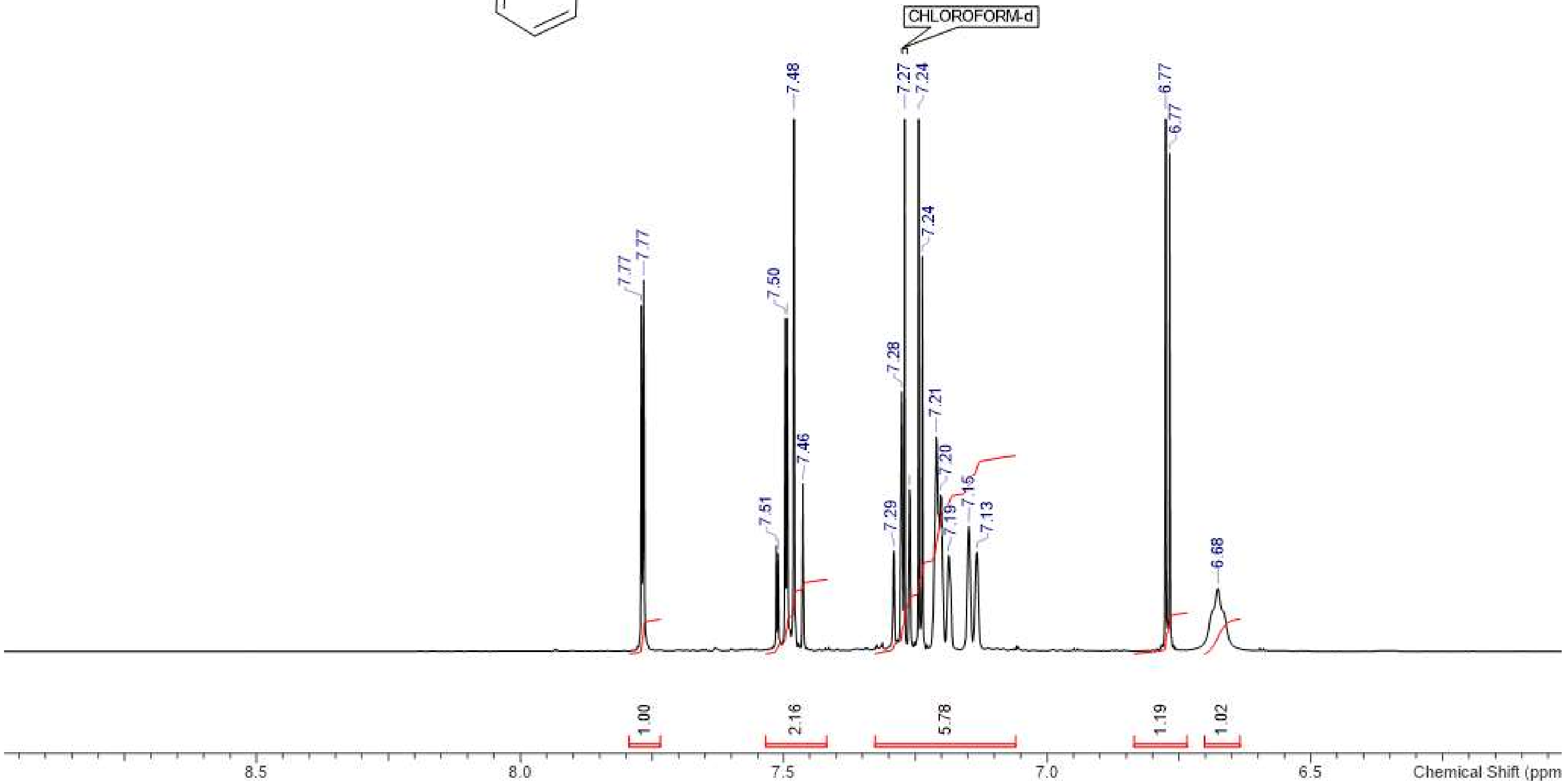

Figure S42: ^{1}H-NMR (500 MHz, $CDCl_3$) zoom of spectrum of 5-(3,4-dichlorophenyl)-*N*-(3-methylbenzyl)furan-2-carboxamide (13)

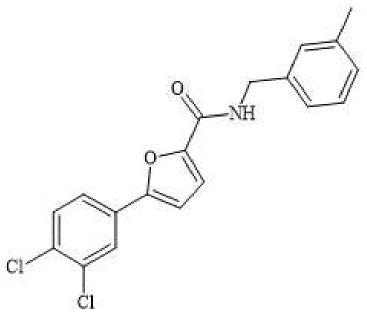

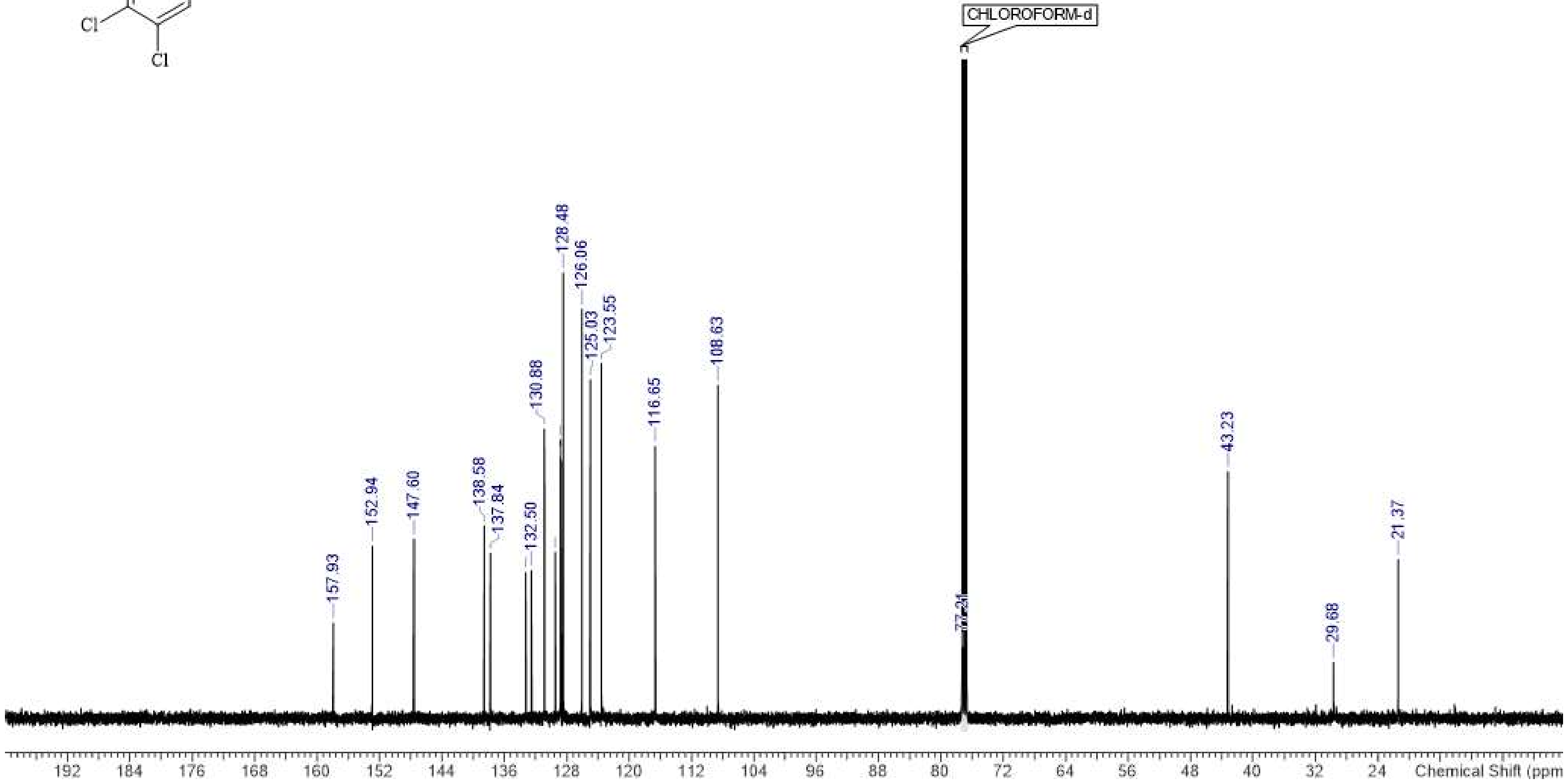

Figure S43: ^{13}C-NMR (126 MHz, CDCl$_3$) spectrum of 5-(3,4-dichlorophenyl)-*N*-(3-methylbenzyl)furan-2-carboxamide (13)

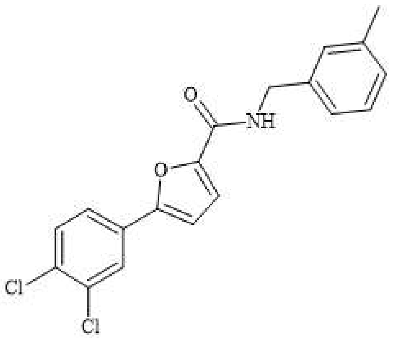

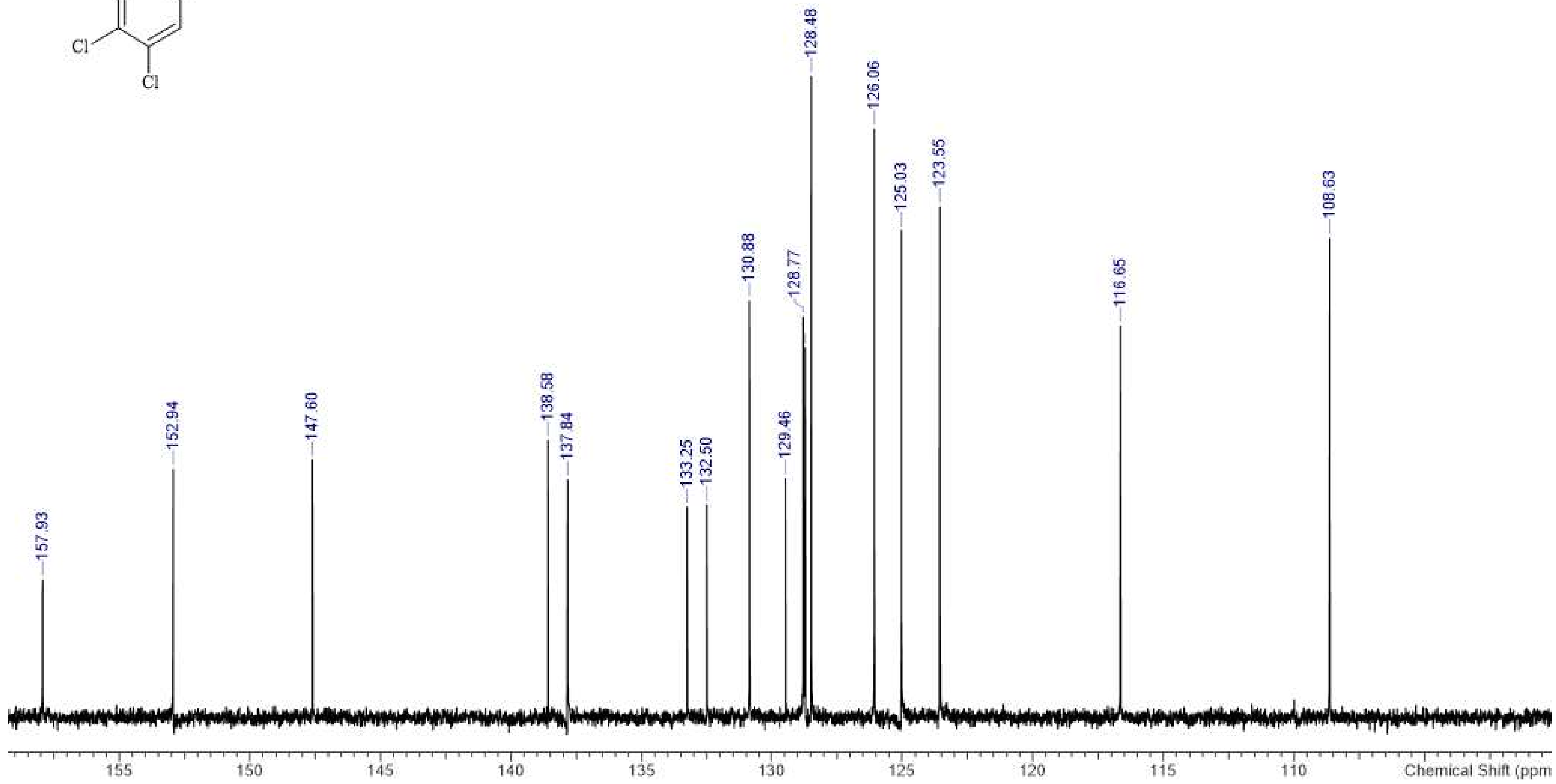

Figure S44: ^{13}C-NMR (126 MHz, $CDCl_3$) zoom of spectrum of 5-(3,4-dichlorophenyl)-*N*-(3-methylbenzyl)furan-2-carboxamide (13)

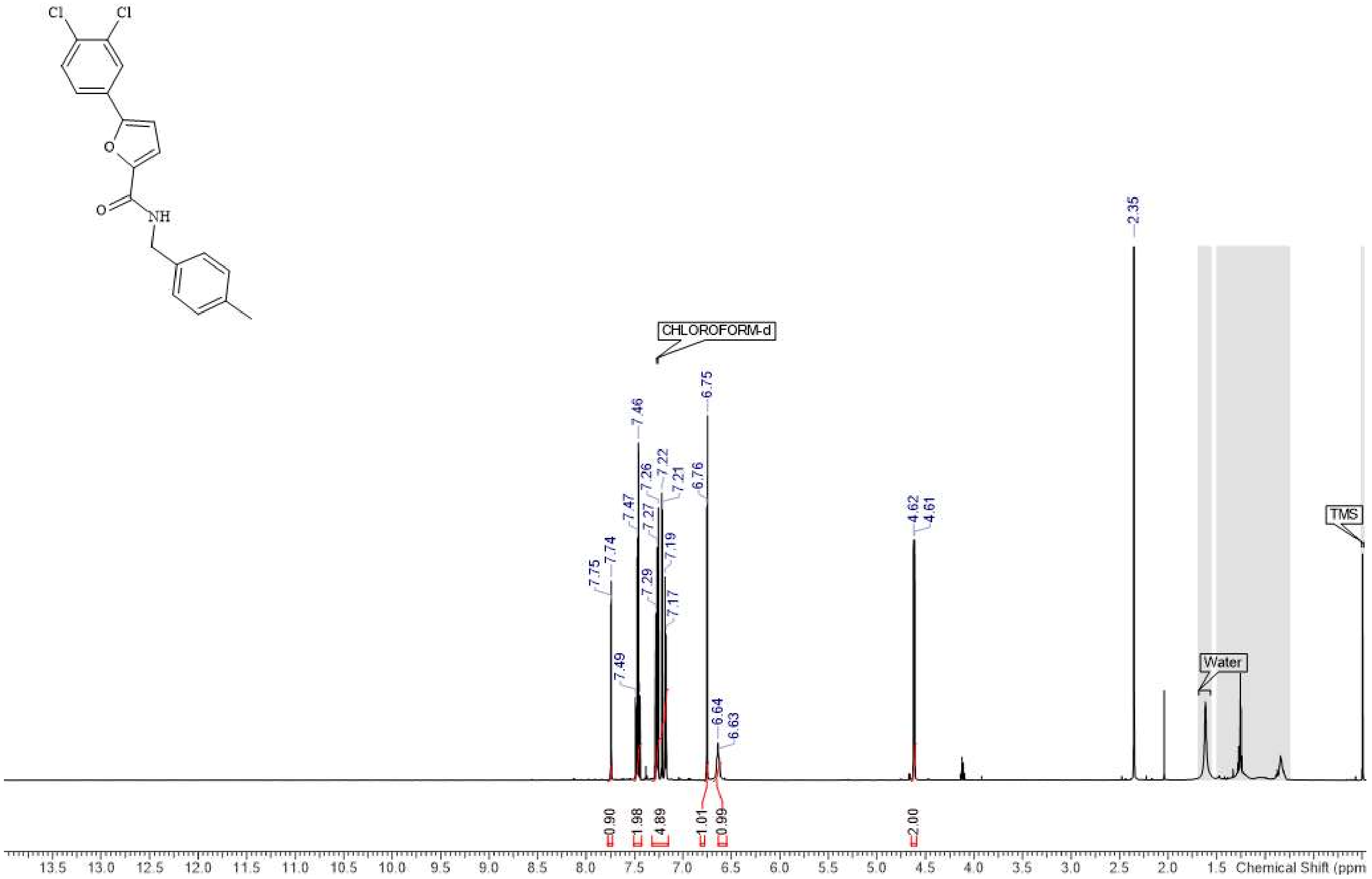

Figure S45: ^{1}H-NMR (500 MHz, $CDCl_3$) spectrum of 5-(3,4-dichlorophenyl)-*N*-(4-methylbenzyl)furan-2-carboxamide (14)

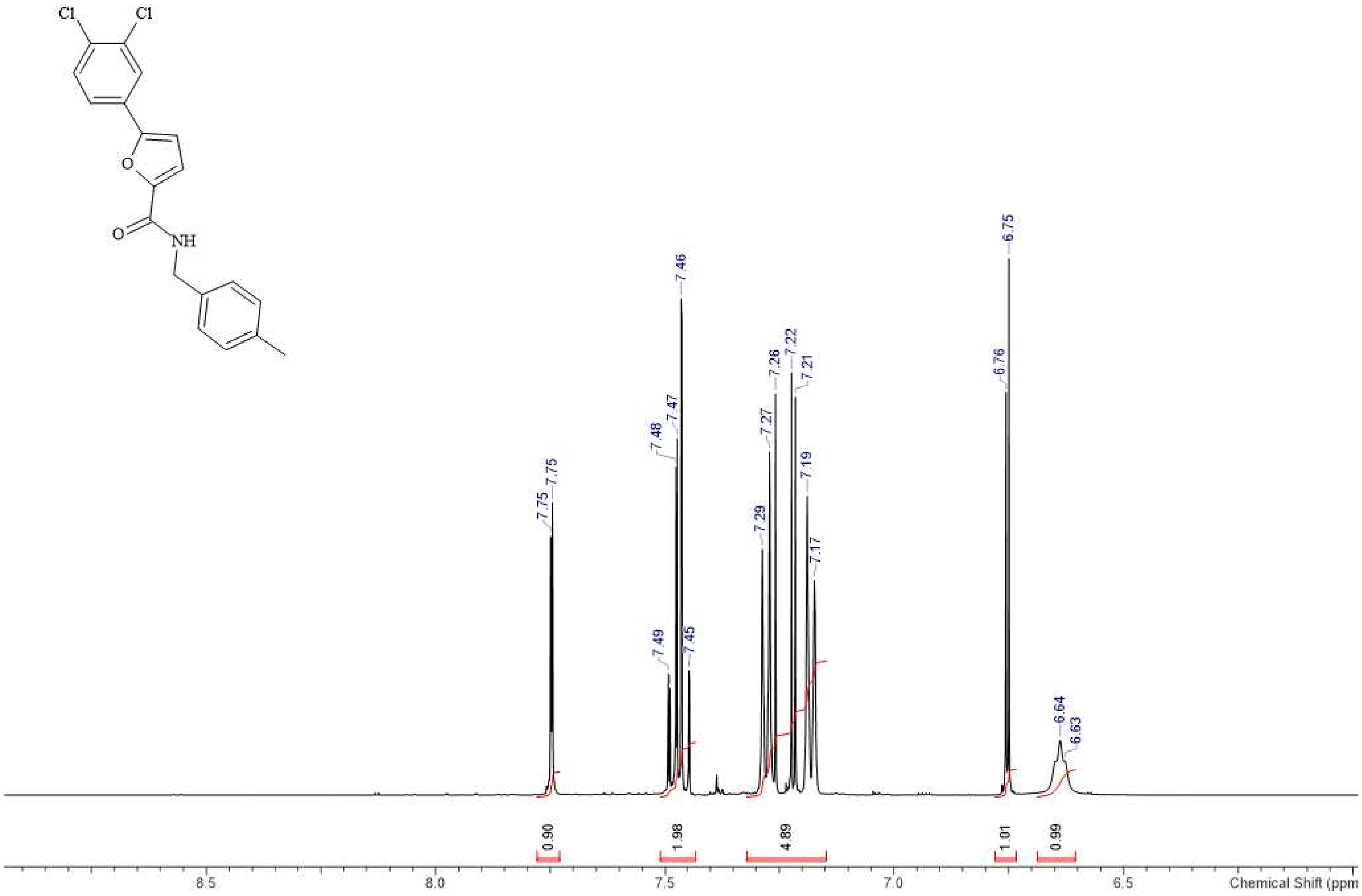

Figure S46: ^{1}H-NMR (500 MHz, CDCl$_3$) zoom of spectrum of 5-(3,4-dichlorophenyl)-*N*-(4-methylbenzyl)furan-2-carboxamide (14)

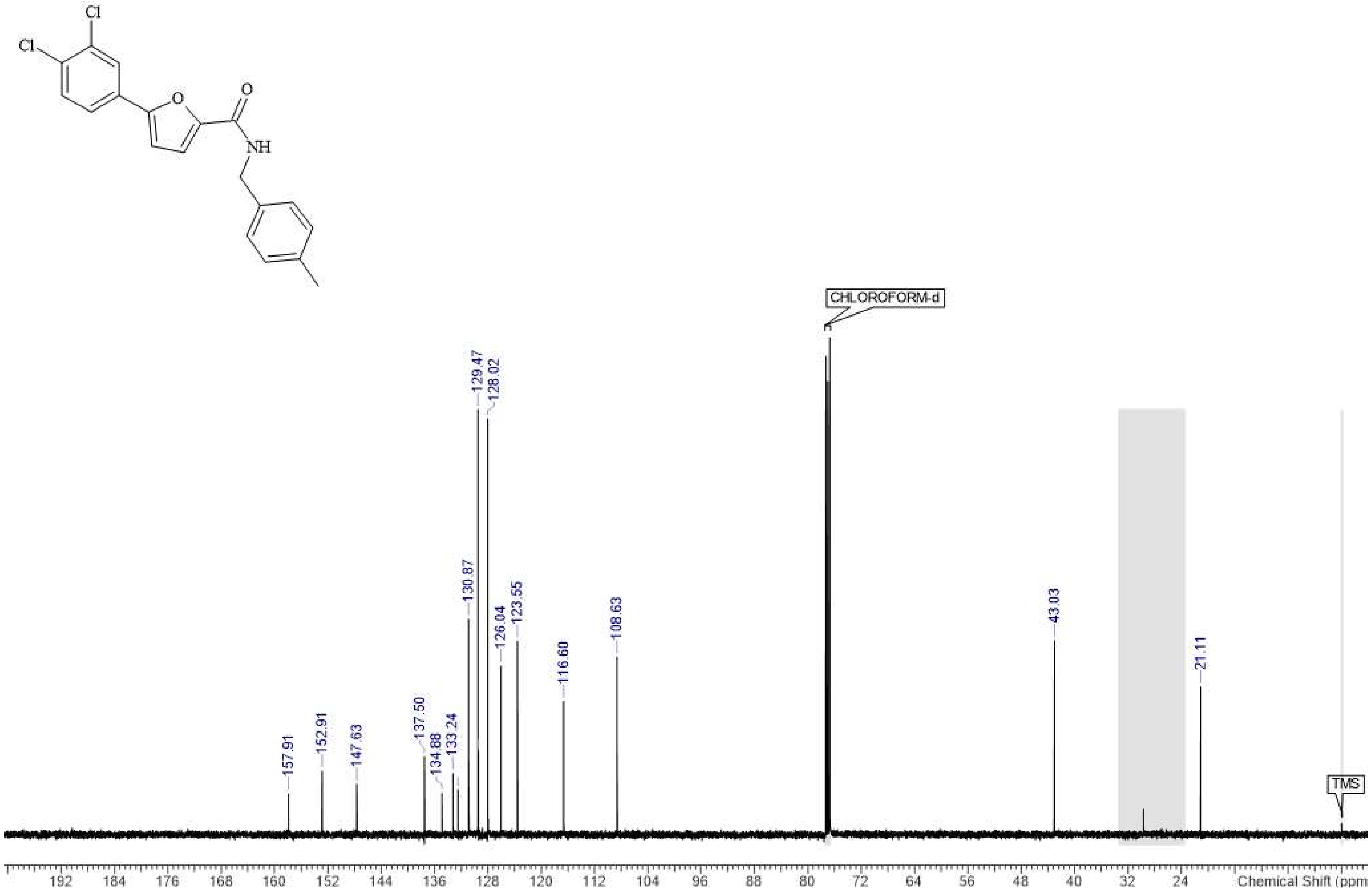

Figure S47: ^{13}C-NMR (126 MHz, $CDCl_3$) spectrum of 5-(3,4-dichlorophenyl)-*N*-(4-methylbenzyl)furan-2-carboxamide (14)

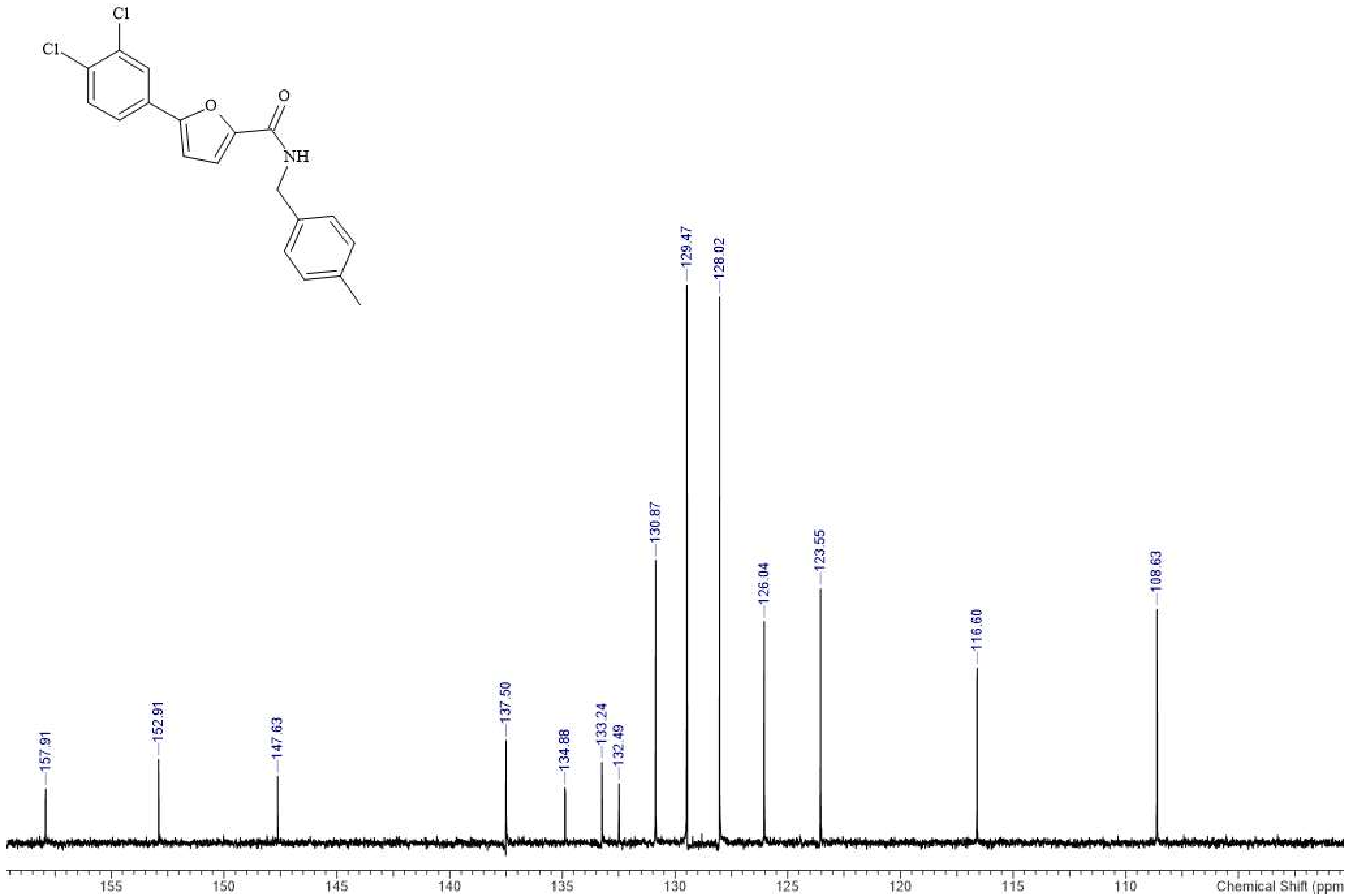

Figure S48: ^{13}C-NMR (126 MHz, $CDCl_3$) zoom of spectrum of 5-(3,4-dichlorophenyl)-*N*-(4-methylbenzyl)furan-2-carboxamide (14)
